# Paramedic analgesia comparing ketamine and morphine in trauma (PACKMaN): a randomised, double-blind, phase 3 trial

**DOI:** 10.1016/j.lanepe.2025.101265

**Published:** 2025-04-05

**Authors:** Michael A. Smyth, Hannah Noordali, Kath Starr, Joyce Yeung, Ranjit Lall, Felix Michelet, Gordon Fuller, Stavros Petrou, Alison Walker, Zoe Green, Rebecca McLaren, Elisha Miller, Duncan Buckley, Gavin D. Perkins

**Affiliations:** a University of Warwick, Gibbett Hill Road, Coventry CV4 7 AL, UK; b University Hospital Coventry and Warwickshire NHS Trust, Clifford Bridge Road, Coventry CV2 2DX, UK; c University Hospitals Birmingham NHS Foundation Trust, Mindelsohn Way, Birmingham B15 2GW, UK; d Population Health, School of Medicine and Population Health, University of Sheffield, 30 Regent Street, Sheffield S1 4DA, UK; e Nuffield Department of Primary Care Health Sciences, Radcliffe Observatory Quarter, University of Oxford, Woodstock Road, Oxford OX2 6GG, UK; f West Midlands Ambulance Service University NHS Foundation Trust, Waterfront Business Park, Dudley DY5 1LX, UK; g Yorkshire Ambulance Service NHS Foundation Trust, Wakefield 41 Business Park, Brindley Way, Wakefield, West Yorkshire WF2 0XQ, UK; h NIHR Academy, 21 Queen Street, Leeds, West Yorkshire LS1 2TW, UK

**Keywords:** Pain, Analgesia, Ketamine, Morphine, Ambulance, Paramedic, EMS, Prehospital

## Abstract

**Background:**

Paramedics frequently administer analgesic medications for pain following trauma. Morphine is the most commonly administered strong analgesic. However, it may not be the best option as it may lower blood pressure, depress respiration and there is a risk of dependency. Ketamine might be a better option due to speed of onset and favourable side-effect profile. We sought to compare clinical effectiveness of paramedic administered ketamine and morphine in patients with severe pain following trauma.

**Methods:**

PACKMaN was a double-blinded, randomised controlled, superiority trial, conducted in two regional ambulance services in the UK. Eligible patients were 16 years of age or over, had an acute injury, and articulated a pain score of 7 or greater on a 0–10 numeric rating score (NRS). We excluded pregnant patients, prisoners, those unable to articulate a pain score and anyone lacking capacity. The randomisation list prepared by the study programmer, utilised a permuted, unstratified, block randomisation system (variable size blocks) to achieve an overall ratio of 1:1 control (morphine): intervention (ketamine). Treatment packs were identical in appearance, apart from their unique sequential number. Individual participant randomisation occurred when the attending paramedic opened the treatment pack. The maximum available dose of morphine was 20 mg while the maximum available dose of ketamine was 30 mg. The treating paramedic administered the trial drug slowly, in regular small aliquots, via the intravenous (or intraosseous) route, titrating treatment until the patient reported adequate analgesia or requested that treatment stop due to undesired side effects. Timing of drug administration was not prespecified. The primary outcome was the Sum of Pain Intensity Difference (SPID) score on arrival to the hospital, calculated using patient reported NRS scores. Analysis was performed on an intention to treat basis. PACKMaN is registered with the International Clinical Trials Registry (ISRCTN14124474).

**Findings:**

PACKMaN recruited its first patient on 10/11/2021 and achieved its recruitment target on 16/05/2023. We randomised 449 participants: 219 (49%) received ketamine and 230 (51%) received morphine. The SPID score was 3.5 (SD 2.8) for ketamine and 3.4 (SD 3.0) for morphine. We found no significant difference in efficacy between drugs (adjusted mean difference 0.1, 95%CI −0.4 to 0.6, p = 0.74). There was no significant difference in the incidence of serious adverse events [4 (2%) ketamine; 8 (3%) morphine]. There were no treatment related deaths.

**Interpretation:**

Ketamine did not provide superior analgesia than morphine when used by paramedics to treat acute severe trauma pain. Unexpected adverse events occurred infrequently. Despite analgesia, many patients still experienced pain on arrival at hospital, highlighting the need for further research.

**Funding:**

PACKMaN was funded by the 10.13039/501100000272National Institute for Health and Care Research.

**Research in context**

**Evidence before this study**

We searched Medline and Embase (up to 28/01/2025) comparing ketamine with any other agent in the emergency setting. Searches were based upon key words including EMS, prehospital, paramedic, analgesia, pain relief, trauma, injury, ketamine and morphine. No language restrictions were placed. Our search identified 6 randomised trials (RCTs), 22 observational studies and 4 systematic reviews. Only 2 RCTs compared ketamine with morphine analgesia in the prehospital environment. Both indicate that ketamine is not inferior to morphine. The 22 observational studies suggest that ketamine is as effective or better than morphine while the 4 systematic reviews conclude that ketamine is at least as effective as morphine with fewer side effects.

**Added value of this study**

To the best of our knowledge this is the first blinded, randomised trial to compare the pre-hospital administration of ketamine and morphine by paramedics, for severe pain secondary to trauma. Our study found that ketamine produced a similar level of pain relief compared to morphine. Approximately one in every two patients treated experienced adverse events related to their injuries or the treatment received. The pattern of adverse events differed with morphine being more likely to precipitate desaturation (16%) and hypotension (10%) whereas ketamine was more likely to result in an adverse behavioural reaction (10%). Despite parenteral analgesia, over half the patients enrolled continued to experience moderate or severe pain at the time of arrival at hospital.

**Implications of all the available evidence**

Ketamine is safe in the hands of ALS paramedics and produces similar levels of pain relief to morphine. Despite parenteral analgesia, many patients are left in pain on arrival at hospital, highlighting the need for further research.

## Introduction

It has been reported that trauma accounts for 24% of UK ambulances service workload.^1^ Up to 70% of trauma calls involve patients experiencing pain.^2^ Pain is defined by the International Association for the Study of Pain (IASP) as “an unpleasant sensory and emotional experience associated with, or resembling that associated with, actual or potential tissue damage.”^3^ the World Health Organisation (WHO) assert that management of pain is a fundamental human right.^4^

Although ambulance staff are empowered to administer parenteral analgesic drugs, many prehospital patients report inadequate pain relief.^5^ There are several potential reasons for this. The prehospital interval is short and there may be insufficient time for drugs to reach their therapeutic effect. Training of paramedics may be inadequate, altered patient physiology and a limited formulary may all contribute to suboptimal prehospital pain relief.

The analgesic most frequently administered for acute severe traumatic pain, by advanced life support (ALS) paramedics in the United Kingdom (UK), is morphine.^6^ Morphine has several side effects including nausea, confusion, dizziness, drowsiness, respiratory depression, hypotension and arrhythmia that may limit its use.^7^^,^^8^ These side effects, and concerns related to potential longer-term dependence, may limit effective use by clinicians.^9^

Ketamine has been advocated as an ideal prehospital analgesic due to its favourable pharmacokinetics.^10^ It acts primarily by antagonising N-methyl D-aspartate (NMDA) receptors.^11^ It exerts its effect by “disconnecting” the thalamocortical and limbic systems, effectively dissociating the central nervous system (CNS) from outside stimuli (e.g., pain, sight, sound).^12^ Ketamine has a rapid onset, short duration of action and large therapeutic window, all of which make it an appealing option with a relatively low-risk profile.^13^ Furthermore, it has a distinct dose–response gradient in which small doses (<0.5 mg/kg) provide an analgesic effect while larger doses (>2 mg/kg) produce an anaesthetic effect.^14^

Although ketamine has been evaluated in the emergency department setting,^15^, ^16^, ^17^, ^18^, ^19^, ^20^, ^21^, ^22^, ^23^ it has not been widely studied in the prehospital context. Two randomised trials, in physician led pre-hospital systems, have compared ketamine and morphine.^24^^,^^25^ Both studies were non-inferiority studies and at high risk of bias due to their open-label design. This limits interpretation due to the subjective nature of their primary outcomes (pain scores).^26^ The relative lack of evidence, and the need to identify an effective prehospital analgesic prompted the UK National Institute for Health and Care Excellence (NICE) to highlight the need for quality evidence.^27^ We believe PACKMaN is the first double-blind, randomised trial to determine if ketamine is superior to morphine for the management of acute severe traumatic pain, when administered by ALS paramedics.

## Methods

### Study design

PACKMaN was a multi-centre, parallel, individually randomised, controlled, double-blind superiority trial comparing the clinical and cost-effectiveness of ketamine and morphine for severe pain following acute traumatic injury. This pragmatic phase III trial was conducted in two regional ambulance services in England. West Midlands Ambulance Service University NHS Foundation Trust (WMAS) serves a population of 5.6 million covering an area of 5000 square miles. It operates a fleet of over 600 ambulances from 15 ambulance hubs. Yorkshire Ambulance Service NHS Trust (YAS) serves a population of 5 million covering more than 6000 square miles. It operates a fleet of over 500 ambulances from 62 ambulance stations. The trial operated across 5 ambulance hubs in WMAS, and 41 ambulance stations in YAS.

Patient and public involvement (PPI) representatives were involved throughout this study. A PPI collaborator, with experience of trauma and chronic pain, helped to develop the study protocol advised on study conduct and agreed to co-produce materials to disseminate our findings. We also co-designed patient facing materials in collaboration with members of the “Clinical Research Ambassador Group” (CRAG) from University Hospital Birmingham NHS Foundation Trust. Two PPI members served on the Trial Steering Committee.

Ethical approval was granted by the West of Scotland Research Ethics Committee (20/WS/0126). PACKMaN recruited its first patient on 10/11/2021 and achieved its recruitment target on 16/05/2024. The trial protocol has previously been published.^28^

### Participants

Patients were eligible for inclusion if they had suffered an acute traumatic injury, were at least 16 years of age or over, reported a pain score of 7 or greater on a 0–10 numeric rating score (NRS) and, in the opinion of the attending paramedic, would normally require parenteral morphine for analgesia.

We excluded patients with known or suspected pregnancy, inability to articulate severity of pain using the 0–10 NRS, a lack of capacity due to a reason other than pain, known prisoner or they declined participation. In addition, patients who received parenteral ketamine or opioids (morphine, fentanyl) prior to randomisation, or who had a contraindication to either drug, were also excluded. As this was a pragmatic trial, we did not place any restrictions on, or collect details of, the use of non-pharmacologic pain management strategies such as splinting—paramedics could adopt whichever non-pharmacologic strategies they deemed appropriate.

Patients were screened by attending paramedics. Verbal assent to participation was sought by the attending paramedic prior to randomisation. Informed written consent was obtained at a later stage by trained research paramedics, either during the patient’s hospital stay, or following their discharge from hospital. Trial data and individual patient characteristics were extracted from data routinely collected in Ambulance Service patient records and uploaded by research paramedics into the trial database.

### Randomisation and masking

Trial drugs were manufactured and supplied by a pharmaceutical company licenced by the Medicines and Healthcare Products Regulatory Authority (MHRA) to manufacture drugs for research purposes (ModePharma Ltd, London, UK). Ketamine hydrochloride was supplied in 2 ml glass ampoules containing 15 mg in 1 ml of solution. Morphine sulphate was supplied in identical 2 ml glass ampoules containing 10 mg in 1 ml of solution. Trial drugs were packaged in sequentially numbered treatment packs by the pharmaceutical company according to a restricted randomisation list prepared by the study programmer (Stata SE 18.0), which ensured allocation concealment. A permuted, unstratified, block randomisation system (variable size blocks) was used with an overall ratio of 1:1 control: intervention.

Treatment packs were identical in appearance, apart from their unique sequential number. Packs were distributed to the participating ambulance services by the trial drug manufacturer to ensure equal proportions of ketamine (intervention) and morphine (comparator) across participating sites. Paramedics signed out trial packs from controlled drug stores on a shift-by-shift basis. If the treatment pack was not used during the course of a shift, it would be returned to the controlled drug store. Individual participant randomisation occurred when the attending paramedic opened the trial treatment pack. Allocation was concealed from all study personnel except the trial statistician.

### Procedures

After randomisation, the trial drugs were prepared by the attending paramedic by diluting the content of one ampoule with 9 ml of 0.9% sodium chloride in a 10 ml syringe to produce a 10 ml volume. This created a final concentration of 1  mg ml^−1^ morphine or 1.5  mg ml^−1^ ketamine. Administration of trial drug mirrored standard practice for administration of morphine. The trial drug was administered by slow intravenous (IV) or intraoseous (IO) injection, titrated to effect over 5 min (approximately 2 ml per minute), aiming to give the minimal effective dose. If the patient continued to report pain 5 min after receiving the first full syringe (10 ml), a second syringe was prepared and administered in a similar manner by the attending paramedic. A maximum of 20 mls of trial drug could be administered, equating to a maximum dose of either 20 mg morphine or 30 mg ketamine.

### Outcomes

The primary outcome was the Sum of Pain Intensity Difference (SPID) score, as advocated in the Initiative on Methods, Measurement, and Pain Assessment in Clinical Trials guidance.^29^ SPID is defined as difference in pain intensity over the assessment period.^30^ In our study, pain intensity was measured using a 0–10 NRS, documented by the attending paramedic at regular intervals. We did not specify how frequently pain scores should be documented, as this was a pragmatic trial mirroring routine practice. The time measurement was the interval in hours between each NRS score. Secondary outcomes, including the time points at which they were measured, are reported in Table 1.

**Table 1:** Secondary outcomes and timepoints for measurement.

Secondary outcome	Time point			
	Baseline/prehospital	After hospital arrival	3 months	6 months
Vital signs	✓			
TOTPAR	✓			
Side effects & adverse events	✓	✓	✓	✓
Time to minimal improvement	✓			
Time to much improvement	✓			
Time to very much improvement	✓			
Time to peak analgesia	✓			
Duration of analgesia	✓			
Proportion of patients with pain score below 4/10	✓			
Patient Global Impression of Change	✓			
Rescue analgesia	✓			
Resource use	✓	✓	✓	✓
Quality of Life—EQ-5D-5L			✓	✓
Questionnaire—BPI-SF			✓	✓
Questionnaire—CSRI			✓	✓

TOTPAR—total pain relief,^29^ EQ-5D-5L—European quality of life 5 dimensions with 5 levels questionnaire,^31^ BPI-SF—brief pain inventory short form,^32^ CSRI—clinimetric self-reported instrument.

Trial data were extracted from routinely collected electronic patient care records by research paramedics and uploaded to the trial database using a predefined data entry template. Ambulance services amended their respective electronic patient care records to collect trial specific outcomes, such as Global Impression of Change. No supplemental data collection instrument was used by participating paramedics.

Research paramedics contacted participants who consented to long-term follow-up. Quality of life (EQ-5D-5L) and chronic pain (BPI-SF) assessments were completed by research paramedics, either face to face, or virtually on request of the patient.

A cost effectiveness analysis was undertaken and will be published as a separate publication. Cost effectiveness was assessed from a UK National Health Service (NHS) and personal social services (PSS) perspective and from a societal perspective. Costs (£ 2021–2022 prices) were collected prospectively over a 6-month follow-up period. A bivariate regression of costs and quality-adjusted life-years (QALYs) was conducted to estimate the incremental cost per QALY gained and the incremental net monetary benefit of ketamine in comparison to morphine.

### Statistical analysis

A recent meta-analysis of studies reporting the minimally clinically important difference (MCID) in pain scores concluded that an 8 mm–40 mm reduction in 100 mm visual analogue scale (VAS) was the MCID for patients experiencing pain.^33^ Our study utilises NRS rather than VAS to quantify pain intensity. VAS are frequently converted to NRS, with each 10 mm on the VAS be equated to 1-point on the NRS, and decimals being rounded to the nearest integer.^34^ A meta-analysis by Wewege et al. concluded that converting VAS to NRS does not introduce bias.^35^ This would suggest that the MCID in our study would require a 1 to 4 points reduction on the NRS. Adopting a 1-point reduction in NRS would require a larger sample size, and therefore provide a more precise estimate, than seeking to identify a 4-point reduction in NRS.^33^

Our sample size calculation assumes a standard deviation of 3.0,^18^^,^^19^^,^^25^ 1:1 randomisation, a power of 90%, significance level of 5% and a withdrawal/non-response rate of 15%.^23^^,^^24^^,^^36^ We calculated our trial would require a sample of 446 participants, recruiting 223 to each arm of the study, to detect a 1-point difference (0–10 NRS) in effectiveness between morphine and ketamine.

All analyses were conducted using the intention to treat principle. The morphine group was the reference group for the analysis. The primary analysis was the adjusted analysis. All planned analyses are detailed in the Statistical Analysis Plan (Supplemental data file, pg 3) and Health Economic Analysis Plan (Supplemental data file, pg 87).

The primary outcome was analysed for all randomised participants who received their allocated intervention prior to hospital arrival and had baseline, and at least one subsequent pain score, before arriving at hospital. The trial ended when the patient arrived at the hospital. We did not collect pain scores or details of pain relief administered after hospital arrival as this was not the remit of the trial. We used a linear regression model to calculate the SPID score.

The secondary outcomes, total pain relief (TOTPAR),^37^ time to achieving minimal, much and very much improvement, proportion of patients achieving a pain score below 4/10 and duration of analgesia also required baseline and one subsequent pain score prior to arrival at hospital. Safety outcomes were assessed for all participants who received a trial drug. The prespecified covariates for adjusted analysis were age (<60; ≥60 years), sex, weight (cut point on the 33.33% and 66.67% percentile) and parenteral IV paracetamol prior to randomisation (as a dichotomy split by yes or no).

Continuous secondary outcomes were analysed in the same manner as the primary outcome. Cox’s proportional hazard model was employed to estimate time to event outcomes and duration of analgesia. Kaplan–Meier plots were used to summarise time to event outcomes (time to minimal improvement, much improvement, very much improvement, peak analgesia and duration of analgesia). Safety outcomes were assessed via a logistic regression model, with odds ratios presented. Other binary outcomes were analysed in the same manner. Participant global impression of change was analysed using ordered logistic model. Participant vital signs were analysed using a linear mixed effect model. The model used outcome data collected throughout the ambulance journey with fixed effects of treatment group and prespecified adjustment covariates. Correlation between measures was modelled using an autoregressive structure of order 1.^38^ For the vital sign Glasgow Coma Scale an ordered logistic mixed effect model was used, with the same fixed effects as for the linear mixed effect model. All secondary analyses are presented with 95% CI and p-values.

A pre-planned post hoc subgroup analysis by age, sex, and parenteral IV paracetamol prior to randomisation was performed. This analysis was limited to the primary outcome and was unadjusted. To test whether the treatment effect was different between the subgroups we fitted linear regression models for the primary outcome with the inclusion of an interaction term. Prespecified sensitivity analyses included a complier average causal effect (CACE) analysis for participants that did not comply with the study drug infusion protocol. We planned to use Pocock’s win-ratio method if there were more than 5% death affecting assessment of the primary outcome. No imputation was planned due to the nature of the primary outcome calculation, in that the primary outcome could be obtained even if there were missing timepoint/data points by extrapolating and using data which were available (ambulance crews are required to document at least 2 sets of vital signs whenever they administer analgesics). The Data Monitoring Committee (DMC) and Trial Steering Committee (TSC) met twice a year to assess safety data and trial progress. No formal interim analysis was planned.

Post-hoc analyses, outside of the scope of the Statistical and Health Economic Analysis Plan, were conducted to aid in the conclusion of the study. These included comparing the incidence rate of participants achieving minimal, much, and very much improvement^37^ across treatment arms, using Cox’s proportional hazard model. We also sought to compare the incidence rate for use of Midazolam and Naloxone across treatment arms.

Finally, the frequency of side effects reported during the trial suggested participating paramedics may have been able to identify which drug was administered in those patients who displayed prominent side effects characteristic of either drug. We used Fisher’s exact test to assess the adequacy of blinding, by sampling approximately 5% of cases and asking the treating paramedic to identify which drug they thought they had administered.

There was no adjustment for multiplicity, however secondary outcomes should be interpreted as exploratory. Data were analysed using Stata SE 18.0. The trial was registered with the International Standard Randomised Controlled Trial Number registry (ISRCTN14124474) and the European Union Drug Regulating Authorities Clinical Trials Database (EudraCT 2020-000154-10).

### Role of the funding source

PACKMaN was funded by the National Institute of Health and Care Research Health Technology Assessment Programme (128086). The funder played no role in the design of the study, data collection, statistical analysis, interpretation of findings or reporting of results.

## Results

A total of 449 participants were randomised into the trial between 10th November 2021 and 16th May 2023: 219 (49%) to the ketamine group and 230 (51%) to the morphine group. The mean age of the included population was 63.6 years (SD 21.7). Baseline characteristics (age, sex, ethnicity, weight, initial pain score, mechanism of injury, systolic blood pressure, diastolic blood pressure, heart rate, respiration rate, oxygen saturations, Glasgow coma scale, type of injury sustained, and body part injured) were similar across the two treatment groups (Table 2). The mean cumulative dose of ketamine administered was 18.8 mg (SD 7.9 mg), while mean dose of morphine administered was 12.8 mg (SD 5.1 mg). The mean dose per kilogram was 0.24 mg/kg (SD 0.11 mg/kg) for ketamine and 0.17 mg/kg (SD 0.08 mg/kg) for morphine.

**Table 2:** Participant baseline characteristics.

	Ketamine (n = 219)	Morphine (n = 230)
**Age (years), Mean (SD)**	64.0 (21.3)	63.2 (22.1)
**Sex**^b^**, N (%)**		
Male	102 (47%)	106 (46%)
Female	117 (53%)	124 (54%)
**Ethnicity**^a^^,^^b^**, N (%)**		
White	98 (45%)	108 (47%)
Black	0	0
Mixed	0	0
Any other ethnic group	2 (1%)	0
Asian	0	1 (<1%)
Ethnicity not documented	119/218 (54%)	119/230 (52%)
**Weight (kg), Mean (SD)**	75.2 (17.5)	77.6 (19.1)
**Initial pain score (0–10)**^a^**, Mean (SD)**	8.8 (1.3)	8.9 (1.2)
**Mechanism of injury**^a^**, N (%)**		
Blunt Trauma	215 (98%)	227 (99%)
Penetrating Trauma	3 (1%)	2 (1%)
Burn	1 (<1%)	0
**Injuries sustained, N (%)**		
Fracture/dislocation	193 (88%)	210 (91%)
Soft tissue injury	72 (33%)	69 (30%)
Wound/laceration	23 (11%)	34 (15%)
**Body part/region injured, N (%)**		
Head	16 (7%)	21 (9%)
Neck	9 (4%)	15 (7%)
Chest & back	44 (20%)	43 (19%)
Abdomen	6 (3%)	6 (3%)
Pelvis	68 (31%)	62 (27%)
Upper limbs	43 (20%)	60 (26%)
Lower limbs	153 (70%)	149 (65%)
**Vital signs**		
Blood pressure Systolic (mmHg), Mean (SD)	145.6 (24.0)	147.8 (27.8)
Blood pressure Diastolic (mmHg), Mean (SD)	82.0 (15.2)	84.6 (16.2)
Heart rate (bpm), Mean (SD)	81.8 (16.3)	83.4 (16.2)
Respiratory rate (bpm)^a^, Mean (SD)	19.2 (3.4)	19.5 (4.3)
Oxygen saturations (%)^a^, Mean (SD)	97.3 (2.5)	96.8 (3.4)
Glasgow coma score (GCS)^a^ N (%)		
15	214 (99%)	227 (100%)
14	2 (1%)	0
13	0	0
12	1 (<1%)	1 (<1%)
11 or lower	0	0

SD—standard deviation, kg—kilogram, mmHg—millimetres of mercury, bpm—beats per minute, GCS—Glasgow Coma Scale.

a Absolute numbers.

b Self-defined by patient.

**Fig. 1:**
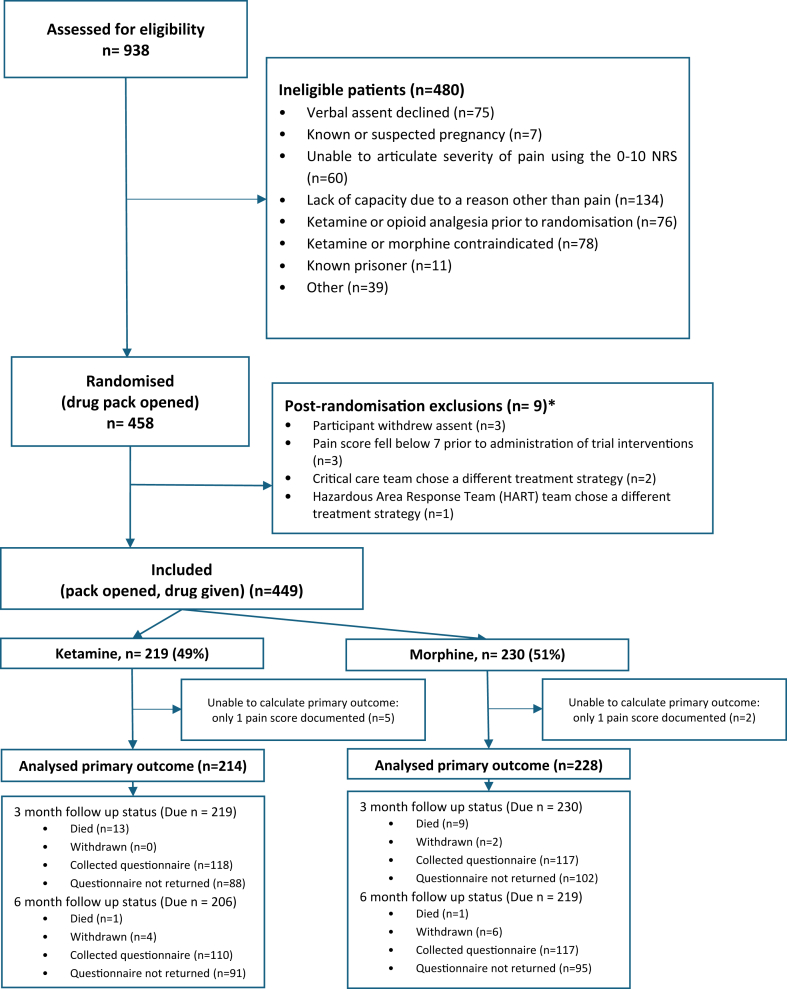
: Trial profile. ∗Participant where drug pack was opened but participant was excluded from trial prior to IMP being administered.

Throughout the timeline of the trial, a total of 938 participants were assessed for eligibility of which 480 (51%) were deemed ineligible. Nine (1%) participants were excluded after randomisation where the drug pack had been opened, but no trial drug was administered (see Fig. 1). For these 9 post randomisation exclusions, 3 withdrew assent, 3 became ineligible due to a spontaneous reduction pain score to below the require NRS threshold of 7/10, and in 3 cases a different (non-trial trained) care team took over care of the individual.

The request to stop recruitment was issued once the target of 446 patients (excluding post randomisation exclusions) was reached. Three additional participants were subsequently registered on the trial database as they had been recruited to the trial before the “stop recruitment” request was issued, but their data had not been entered into the trial database before the “stop recruitment” request was issued. This brought our total randomisations to 449. Of the total randomised, 221 (49%) participants were recruited from YAS and 228 (51%) were recruited from WMAS. The primary outcome was obtained for 98% of participants (214/219, 228/230). Three and six-month follow-up rates were 236 (53%), and 228 (51%) respectively. This level of missingness at follow up was expected and is not believed to have an impact on the findings of this trial. Reasons for loss to follow-up are presented in Fig. 1.

### Effectiveness outcomes

For the primary outcome, we found that ketamine was not superior to morphine. The SPID score was, 3.5 (SD 2.8) for the ketamine group and 3.4 (SD 3.0) for the morphine group (adjusted mean difference 0.1, 95% CI −0.4 to 0.6, p = 0.74; Table 3), suggesting that the overall analgesic effects were similar from randomisation through to handover to hospital care. The likelihood pain would be “very much improved” (more than 56% reduction in pain) was higher for ketamine compared to morphine (Table 3). The onset of action for ketamine was shorter than for morphine (Table 3), whilst the duration of action was shorter for ketamine than for morphine (Table 3 and Supplemental data file, Fig. 1, pg2). The other measures of analgesic efficacy were broadly similar between groups.

**Table 3:** Primary and secondary outcomes.

	N	Ketamine (N = 219)	N	Morphine (N = 230)	Adjusted estimate (95% CI); p-value
**Primary outcome**					
SPID, mean (SD)	214	3.5 (2.8)	228	3.4 (3.0)	MD, 0.1 (−0.4 to 0.6); 0.74
**Secondary outcomes**					
TOTPAR, mean (SD)	214	1.4 (1.1)	228	1.4 (1.2)	MD, 0.03 (−0.2 to 0.2); 0.75
Achieved minimal improvement analgesia, N (%)	214	195 (91%)	228	208 (91%)	OR, 1.0 (0.5–2.0); 0.96
Minutes to minimal improvement^a^, median (IQR)	195	6 (3–15)	208	8 (4–14)	HR, 1.1 (0.9–1.3); 0.32
Achieved “much improvement”, N (%)	214	155 (72%)	228	147 (64%)	OR, 1.5 (0.99–2.2); 0.06
Minutes to “much improvement”^a^, median (IQR)	155	11 (6–25)	147	15 (7–31)	HR, 1.3 (1.1–1.7); 0.01
Achieved “very much improvement”, N (%)	214	122 (57%)	228	105 (46%)	OR, 1.6 (1.1–2.3); 0.02
Minutes to “very much improvement”^a^, median (IQR)	122	15 (6–33)	105	23 (10–37)	HR, 1.4 (1.1–1.8); 0.01
Minutes to peak analgesia^a^, median (IQR)	204	18 (8–36)	217	25 (10–43)	HR, 1.2 (0.96–1.4); 0.12
Duration (minutes) of analgesia^a^, median (IQR)	214	35 (22–51)	228	42 (26–57)	HR, 1.3 (1.0–1.5); 0.01
Required rescue analgesia, N (%)	219	1 (<1%)	230	0	–
Final pain score < 4/10, N (%)	218	89 (41%)	228	86 (38%)	OR, 1.1 (0.8–1.6); 0.59
Minutes from arrival to drug administration, median (IQR)	218	31.5 (23–41)	228	30.5 (23–42)	MD, −0.5 (−4.3 to 3.4); 0.82
Minutes from drug administration to hospital arrival, median (IQR)	219	50 (36–63)	230	51 (35–67)	MD, −1.8 (−6.4 to 2.8); 0.45
**Global impression of change**^b^**N**	216		225		OR, 1.0 (0.7–1.3); 0.78
Very much improved N (%)		63 (29%)		64 (28%)	
Much improved N (%)		83 (38%)		82 (36%)	
Minimally improved N (%)		51 (24%)		62 (28%)	
No change N (%)		16 (7%)		17 (8%)	
Minimally worse N (%)		1 (<1%)		0	
Much worse N (%)		1 (<1%)		0	
Very much worse N (%)		1 (<1%)		0	
**Vital signs**^c^					
Systolic Blood Pressure (mmHg), mean (SD)	216	149.2 (25.0)	225	143.1 (26.4)	MD, 4.6 (0.9–8.2); 0.01
Diastolic Blood Pressure (mmHg), mean (SD)	216	83.2 (15.0)	225	81.2 (15.1)	MD, 1.4 (−0.6 to 3.4); 0.18
Pulse rate (bpm), mean (SD)	216	80.2 (15.5)	224	81.7 (16.1)	MD, −1.6 (−4.1 to 0.8); 0.20
Respiratory rate (bpm), mean (SD)	214	18.3 (2.7)	225	18.6 (3.7)	MD, −0.1 (−0.7 to 0.4); 0.65
Oxygen saturations (%), mean (SD)	215	97.2 (2.6)	225	96.5 (3.3)	MD, 0.6 (0.2–0.9); <0.01
Glasgow coma score (GCS), mean (SD)	209	14.9 (0.4)	219	15 (0.2)	OR, 0.7 (0.2–2.5); 0.56
**Hospital stay**					
ED length of stay (minutes), mean (SD)	191	561.4 (355.8)	189	572.6 (371.8)	MD, −14.3 (−86.6 to 57.8); 0.70
Admitted–hospital, N (%)	206	133 (65%)	210	129 (61%)	OR, 1.1 (0.7–1.8); 0.59
Admitted–critical care, N (%)	133	4 (3%)	129	1 (1%)	OR, 6.7 (0.7–67.5); 0.11
**CT scan usage (N)**	190		191		
Patients scanned, N (%)		63 (33%)		77 (40%)	OR, 0.7 (0.5–1.1); 0.14
Scans performed (N)		178		181	
Scans per patient, mean (SD)		2.8 (2.8)		2.4 (2.2)	MD, 0.4 (−0.4 to 1.2); 0.33
**Chronic pain**					
BPI-SF (3 months), (N)	118		118		
Overall pain severity, mean (SD)	114	4.3 (2.3)	116	4.3 (2.4)	MD, −0.01 (−0.6 to 0.6); 0.98
Worst pain, mean (SD)	114	5.7 (2.9)	116	5.8 (3.1)	MD, −0.1 (−0.9 to 0.7); 0.82
Least pain, mean (SD)	114	3.0 (2.3)	112	2.9 (2.2)	MD, 0.1 (−0.5 to 0.7); 0.75
Average pain, mean (SD)	113	4.5 (2.4)	111	4.5 (2.4)	MD, 0.1 (−0.6 to 0.7); 0.86
Pain now, mean (SD)	114	3.9 (2.8)	111	3.7 (2.8)	MD, 0.2 (−0.5 to 0.9); 0.60
Pain interference, mean (SD)	115	4.4 (2.8)	113	4.9 (3.0)	MD, −0.5 (−1.2 to 0.3); 0.21
BPI-SF (6 months), (N)	110		118		
Overall pain severity, mean (SD)	106	4.0 (2.6)	114	4.0 (2.4)	MD, 0.1 (−0.6 to 0.7); 0.77
Worst pain, mean (SD)	106	5.2 (3.2)	114	5.4 (3.2)	MD, −0.1 (−1.0 to 0.7); 0.75
Least pain, mean (SD)	106	3.0 (2.5)	114	2.8 (2.3)	MD, 0.2 (−0.4 to 0.8); 0.52
Average pain, mean (SD)	105	4.3 (2.8)	113	4.3 (2.7)	MD, 0.1 (−0.6 to 0.8); 0.80
Pain now, mean (SD)	106	3.5 (2.8)	114	3.4 (2.6)	MD, 0.2 (−0.5 to 0.9); 0.53
Pain interference, mean (SD)	104	4.2 (2.9)	115	4.0 (2.9)	MD, 0.2 (−0.6 to 1.0); 0.61

SPID—Sum of Pain Intensity Difference, TOTPAR—Total Pain Relief. For the SPID and TOTPAR a higher score indicates a better outcome. SD—standard deviation, MD–mean difference, HR–hazard ratio, IQR—inter quartile range, OR–odds ratio. BPI-SF—Brief Pain Inventory—Short Form (scale of 1–11 where the higher score indicates more pain, overall pain severity is an average of these four questions, pain interference is an average of other questions on the BPI form about how pain interferes with everyday life).

Rescue analgesia–administration of open label ketamine or morphine before hospital arrival.

a Totals shown excludes censored participants and participants with missing data.

b Proportional odds assumption is not violated.

c These outcomes have been modelled using a mixed effect model.

There was no significant difference between drugs in proportion of participants with a final pain score below 4 or the reported global impression of change. There was no significant difference between drugs with respect to chronic pain at either 3 months, or 6 months post randomisation.

### Emergency department (ED) outcomes

There was no significant difference between drugs with respect to length of emergency department (ED) stay, hospital admission, critical care admission or number of computed tomography (CT) scans required (Table 3).

### Physiology

There were minor differences between drugs with respect to the physiologic response to ketamine and morphine. Although we identified statistically significant differences in systolic blood pressure (adjusted mean difference 4.6, 95% CI 0.9–8.2, p = 0.01) and oxygen saturations (adjusted mean difference 0.6, 95% CI 0.2–0.9, p = 0.004), these may not have been clinically important differences (Table 3). All other vital signs showed no significant difference between the ketamine and morphine groups.

### Safety

The frequency of serious adverse events was low in both groups–4 (2%) occurred in the ketamine group and 8 (3%) occurred in the morphine group. Whilst the overall rates of adverse events were similar–106 (48%) occurred in the ketamine group and 109 (47%) occurred in the morphine group, the patterns differed. Desaturation (adjusted odds ratio 0.4, 95% CI 0.2–0.7, p = 0.004, Table 4) and hypotension (adjusted odds ratio 0.2, 95% CI 0.1–0.6, p = 0.003, Table 4) occurred more frequently in the morphine group, whilst adverse behavioural reactions were more frequent in the ketamine group (adjusted odds ratio 8.6, 95% CI 2.5–29.6, p = 0.001, Table 4).

**Table 4:** Safety outcomes.

	Ketamine (n = 219) [N (%)]	Morphine (n = 230) [N (%)]	Adjusted estimate (95% CI); p-value
Serious adverse events	4 (2%)	8 (3%)	OR, 0.5 (0.1–1.7); 0.26
Experienced adverse event	106 (48%)	109 (47%)	OR, 1.0 (0.7–1.5); 0.84
Airway			
Vomiting	16 (7%)	27 (12%)	OR, 0.6 (0.3–1.2); 0.16
Aspiration	0	1 (<1%)	–
Advanced airway management	1 (<1%)	0	–
Respiratory			
Desaturation	15 (7%)	37 (16%)	OR, 0.4 (0.2–0.7); <0.01
Need for ventilatory support	1 (<1%)	0	–
Cardiac			
Arrhythmia	3 (1%)	4 (2%)	OR, 0.7 (0.1–3.7); 0.71
Hypotension	6 (3%)	23 (10%)	OR, 0.2 (0.1–0.6); <0.01
Hypertension	17 (8%)	10 (4%)	OR, 2.0 (0.9–4.5); 0.11
Neurologic			
Sedation	23 (11%)	13 (6%)	OR, 2.0 (1.0–4.2); 0.05
Excitatory movements	2 (1%)	1 (<1%)	OR, 2.3 (0.2–26.1); 0.51
Adverse behavioural reactions	22 (10%)	3 (1%)	OR, 8.6 (2.5–29.6); <0.01
Other			
Allergic reaction	1 (<1%)	4 (2%)	OR, 0.3 (0.0–2.5); 0.25
Nausea	34 (16%)	53 (23%)	OR, 0.6 (0.4–1.0); 0.06
Naloxone administered	2 (1%)	4 (2%)	OR, 0.5 (0.1–2.8); 0.44
Midazolam administered	7 (3%)	0	–

OR–odds ratio.

Percentages are shown for all randomised participants who received their allocated treatment.

There were insufficient data for any pre-planned sensitivity analyses as all participants complied with the study drug infusion protocol, additionally, the threshold of deaths impacting 5% of participants primary outcome calculation was not achieved. No significant interaction was found between treatment group and age, sex, or alternative parenteral analgesia following our subgroup analysis on the primary outcome (Supplemental data file, Table S1, pg1).

### Blinding

Thirty-two paramedics were invited to identify which drug they believed was administered. Of those 32, 28 (88%) responded. A little over half, 17 (61%), were able to correctly identify which drug had been administered. Paramedics correctly identified 10/15 (66%) of cases where ketamine had been administered and 7/13 (54%) of cases where morphine had been administered, Fisher’s exact test, used to compare the proportion of correct guesses across ketamine and morphine, suggested there was no difference across the two groups (p = 0.74).

## Discussion

The main finding of this double-blind randomised controlled trial is that sub-dissociative ketamine, administered by paramedics, prior to hospital arrival, for the treatment of acute severe pain following trauma, did not provide superior analgesia to that of morphine. Both drugs provided similar levels of pain relief from randomisation through to arrival at hospital. However, patients who received ketamine were more likely to experience “very much improvement” in pain intensity^37^ than patients who received morphine. Time to both “much improvement (44% reduction) and “very much improvement (56% reduction)” was shorter for ketamine than it was for morphine. Conversely, patients who received morphine reported longer lasting analgesic effects compared to patients who received ketamine.

Treatment was generally well tolerated, with a low rate of serious adverse events. Side effect/adverse event profiles differed, with those randomised to morphine more likely to experience hypotension and desaturation while those randomised to ketamine were more likely to experience acute behavioural reactions. These side effects are consistent with the known pharmacodynamic profiles of both ketamine and morphine.

Two previous non-inferiority trials have compared sub-dissociative ketamine and morphine for the prehospital management of acute severe pain following trauma.^24^^,^^25^ Tran et al.^24^ conducted an unblinded cluster-randomized trial among physicians in rural Vietnam. Trauma patients were randomised to either intravenous ketamine (0.2–0.3 mg/kg) or intramuscular morphine (10 mg in adults, 5 mg in children). The rural nature of the trial meant that most patients were transported to the physician, rather than the physician attending the scene. Hence the prehospital interval was considerably longer than in our study–mean time from injury to hospital admission was 3.6 h (95% CI 3.3–4.0 h). Pain intensity was measured using a visual analogue scale (VAS). The final reduction in VAS pain intensity was −3.5 in the ketamine group versus −3.1 in the morphine group. The authors concluded that ketamine yielded an analgesic effect equal to that provided by morphine.^25^

Similarly, Le Cornec et al.^25^ conducted an unblinded randomised trial among physicians in France. Trauma patients with moderate or severe pain (NRS > 4) were randomised to either 2–3 mg (dependent upon weight) of intravenous morphine every 5 min or 20 mg of intravenous ketamine over 2 min followed by a further 10 mg every 5 min. The final reduction in NRS pain intensity was −3.7 (95% CI −4.2 to −3.2) in the ketamine group versus −3.8 (95% CI −4.2 to −3.4) in the morphine group (difference, 0.1 [95% CI −0.7 to 0.9]). The authors concluded that ketamine was a suitable alternative to morphine for patients with acute traumatic pain. Key limitations of both of these studies arise from the unblinded design which combined with the subjective nature of pain scoring risks introducing information bias.^26^

Ketamine is increasingly popular among prehospital critical care teams.^14^^,^^39^ Its primary indications include analgesia for severe pain unresponsive to morphine, procedural sedation and emergency anaestheia.^39^^,^^40^ Although it’s use is becoming more widespread, it is not yet included in clinical guidelines permitting routine use by ALS paramedics in the United Kingdom,^6^ where it has been suggested that its use requires anaesthetic level monitoring, and at least 2 clinicians trained in advanced airway management.^41^

In this trial, patients were subject to routine monitoring, without the need for end tidal carbon dioxide (EtCO2) monitoring, or the presence of 2 clinicians competent to perform endotracheal intubation (ETI). Despite a lower level of monitoring and lack of advanced airway (ETI) capability, patients in our study did not experience an increased incidence of adverse effects. On the contrary, patients who received ketamine experienced a lower incidence of respiratory and cardiovascular side effects compared to those who received morphine. This finding suggests ketamine may be a safer analgesic option for patients with severe pain following trauma, particularly those patients who are cardiovascularly unstable. Furthermore, it may not be necessary to mandate ETI capability or routine anaesthetic level monitoring as a prerequisite to provide sub-dissociative analgesia with ketamine. Relaxing the need for ETI capability and anaesthetic level monitoring will remove an existing barrier to introducing ketamine to ALS ambulances in the UK.

Although a substantial proportion of patients in our trial reported an improvement (reduction) in pain intensity, our results also demonstrate that the majority of patients still had moderate or significant pain on arrival at hospital. Among patients who received ketamine 59% (129/218) still had a pain score of 4/10 or higher on arrival at hospital, whilst among patient’s receiving morphine 62% (142/228) still reported a pain score of 4/10 or higher on arrival at hospital. This may have important implications for longer-term patient outcomes.

The International Association for the Study of Pain define chronic pain as that which persists, or recurs, for more than 3 months.^42^ It is common following trauma, with a reported incidence of 15%–30%, increasing to 62% in patients suffering major trauma.^43^, ^44^, ^45^ Evidence indicates that there is a direct relationship between acute pain severity and risk of subsequently developing chronic pain.^9^ Furthermore, there is also evidence to suggest that effective management of acute pain may reduce the risk of chronic pain and other adverse health outcomes.^46^^,^^47^ Military personnel injured in recent conflicts demonstrate a link between acute pain management and both depression and post-traumatic stress disorder (PTSD), where early aggressive pain management exerts a protective effect on the development of depression (OR 0.40 (95%CI 0.17–0.94) and PTSD OR 0.47 (95%CI 0.34–0.66)).^11^^,^^12^ Our findings indicate that many patients remain in moderate or severe pain on arrival at hospital. There remains a need to improve prehospital pain management; to identify management options that ensure pain intensity is reduced to minimal on arrival at hospital.

Our trial is not without limitations. Because we required patients to provide verbal assent to participate, we were only able to enrol patients who had capacity to understand what was being asked of them. Consequently, it is possible that we were unable to recruit the most severely injured patients, limiting the generalisability of our findings. Furthermore, we were unable to obtain injury severity scores (ISS) to determine if there was any significant difference in treatment response between patients with more minor injuries and those who were more severely injured. We did plan to use entry to the Trauma and Audit Research Network (TARN) as a proxy measure to dichotomise these groups, however a cyberattack on the NHS infrastructure in 2023 severely disrupted collection of this data at one of our sites. As a consequence, we were unable to obtain this data for a significant proportion of enrolled patients and therefore unable to undertake this sensitivity analysis in a reliable manner. Finally, although our post-hoc analysis suggested blinding was adequate overall, it is possible that some paramedics became unblinded as to which drug had been administered when predictable side effects associated with each drug emerged. It is unclear how this might have impacted reporting or how we could have mitigated against this possibility.

### Conclusion

In this blinded randomised trial comparing sub-dissociative ketamine and morphine administered by paramedics we found that ketamine was not superior to morphine for the management of acute severe pain following trauma. However, ketamine was more likely to provide both “much improvement” and “very much improvement” in pain intensity and will achieve these effects more rapidly than morphine. Furthermore, morphine was more likely to precipitate both hypotension and desaturation, while ketamine was more likely to result in altered behaviour.

Although our study was powered as a superiority trial, we believe the findings suggest equivalence for morphine and ketamine. Our results further suggest ketamine is safe for use by ALS paramedics and is likely to be a valuable addition to the standard ALS paramedic formulary. Finally, despite most patients reporting a reduction in pain intensity, many patients still suffered moderate or severe pain on arrival at hospital. Further research is therefore required to identify both the optimal drug, and management approach, for patients experiencing acute severe traumatic pain in the prehospital environment.

## Contributors

MAS proposed the original idea and together with GDP conceptualised the study. MAS and GDP secured funding. ZG, AW, EM and RM acquired the data. HN and KS provided administrative oversight. FM and RL verified the data and carried out the statistical analysis. All authors had access to the full dataset. MAS and GDP produced the first draft the manuscript, which was then critically reviewed by HN, JY, RL, FM, GF, SP, AW, ZG, RM, EM and KS. All authors take responsibility for the decision to submit the manuscript and approved the final submitted version.

## Data sharing statement

Data from the PACKMaN study will be available upon request from the Warwick Clinical Trials Unit (WCTU) Data Sharing Committee 6 months after the publication of the funder report. All applications will be assessed by according to WCTU Standard Operating Procedures available at: https://warwick.ac.uk/fac/sci/med/research/ctu/ctuintranet/qa/sop/sop153_sharingdata_v4.0.pdf. An application form to access data can be obtained from WCTUDataAccess@warwick.ac.uk, which should be submitted to the corresponding author in the first instance. Any data shared will be anonymised–no identifiable data will be available. There is no fixed end-date for data availability.

## Declaration of interests

GDP received project funding from the NIHR and is a member of an NIHR funding committee. SP received support as an NIHR Senior Investigator (award number NF-SI-0616-202402) and from the NIHR Applied Research Collaboration (ARC) Oxford and Thames Valley. MAS, HN, JY, RL, FM, GF, AW, ZG, RM, EM and KS have no conflicts to declare.
