# Supplementary material for: Paramedic analgesia comparing ketamine and morphine in trauma (PACKMaN): a randomised, double-blind, phase 3 trial
Source: Lancet Reg Health Eur. 2025 Apr 5;53:101265. doi: 10.1016/j.lanepe.2025.101265 (PMC12002782; doi:10.1016/j.lanepe.2025.101265)
Supplement: Supplemental data file [file mmc1.pdf]

PACKMaN Supplemental data file

Table S1 Sub-group analysis

Subgroups	Ketamine N; mean(sd)	Morphine N; mean(sd)	Effect estimates (95% CI)	Interaction effect (95% CI); p-value
**Age**				
Age <65	85; 2.9 (2.83)	88; 2.6 (2.25)	0.24 (-0.61 to 1.09)	-0.28 (-1.37 to 0.81); 0.619
Age >65	129; 3.9 (2.78)	140; 3.9 (3.22)	-0.04 (-0.72 to 0.64)	
**Gender**				
female	114; 3.3 (3.02)	122; 3.5 (2.97)	-0.22 (-0.96 to 0.53)	0.59 (-0.5 to 1.67); 0.291
male	100; 3.7 (2.62)	106; 3.3 (2.94)	0.37 (-0.43 to 1.16)	
**Alternative parenteral analgesia administered**				
No	182; 3.5 (2.7)	200; 3.5 (2.91)	0.04 (-0.54 to 0.63)	0.19 (-1.4 to 1.77); 0.816
Yes	32; 3.3 (3.56)	28; 3 (3.27)	0.23 (-1.25 to 1.7)	

Figure S1 Time to effect

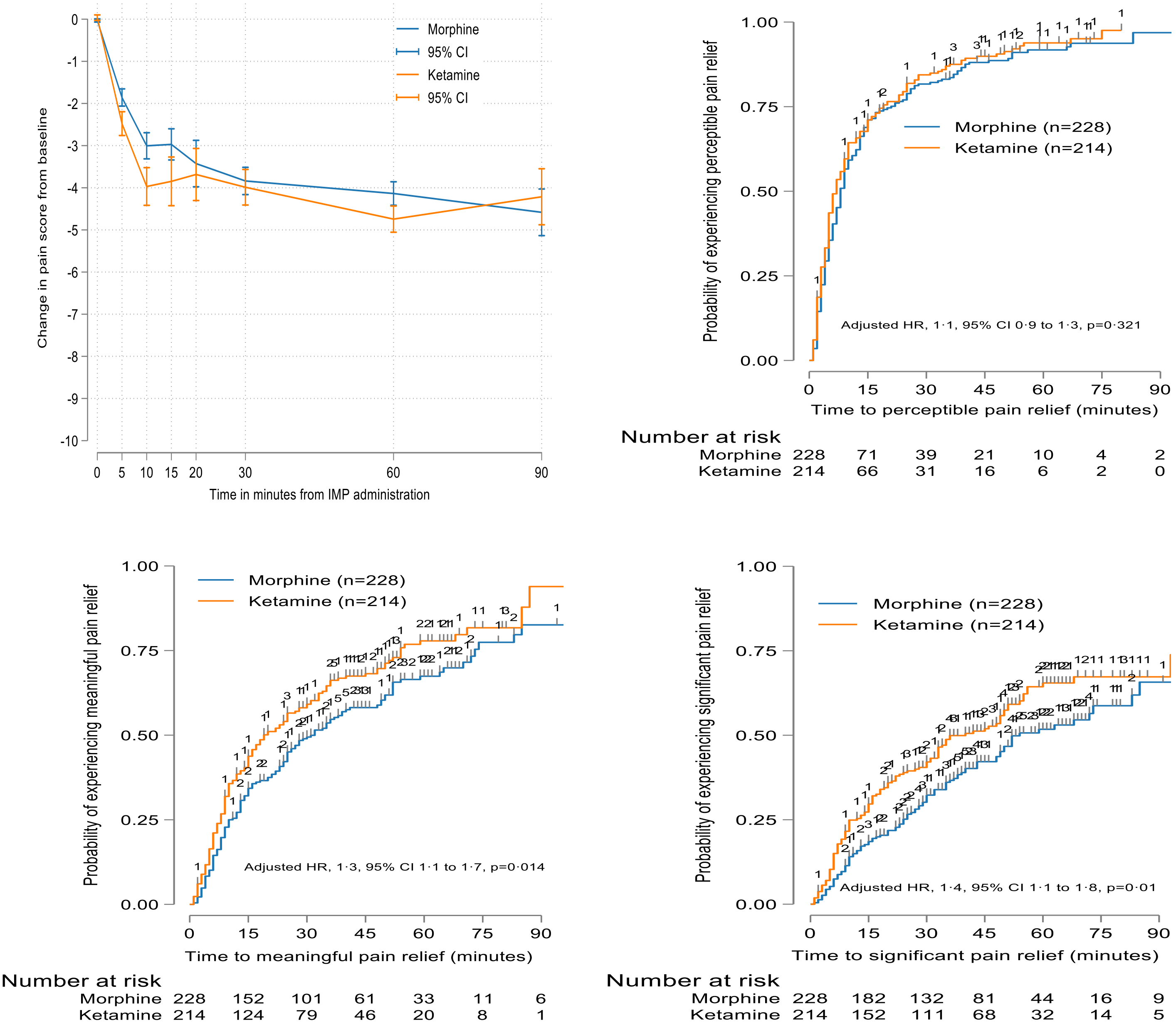

NB - all graphs have been plotted up to 90 minutes, this means that some data is not included in the graphs.

Paramedic Analgesia Comparing Ketamine and MorphiNe in trauma: PACKMaN

STATISTICAL & HEALTH ECONOMIC ANALYSIS PLAN

EudraCT Number: *(if applicable)*	2020-000154-10
ISRCTN: *(if applicable)*	14124474
Funding Body:	National Institute for Health Research
Ethics Approval:	West of Scotland REC 1, approved 01/09/2020
MHRA Approval: *(if applicable)*	13/10/2020
SAP Version:	2.0
Date:	15/02/2024
Protocol version:	8.0, dated 17/01/2024

Contents

1. ADMINISTRATIVE INFORMATION 5

Section 1: Summary of PACKMAN 7

1.1 General principles for the primary statistical and health economic analysis 7

1.2 Trial Design 7

1.3 Objectives 7

1.4 Target population 7

1.5 Outcome measures 8

Section 2: Monitoring of the PACKMAN Trial (operational and statistical) 10

2.1 Operational monitoring 10

2.1.1 Recruitment by ambulance service 10

2.1.2 Recruitment of patients 10

2.1.2 Withdrawals & follow up rates 10

2.1.3 Protocol violations and deviations 10

2.1.4 Safety data 11

2.1.5 Unblinding 11

2.1.6 Paramedic experience 11

2.2 Statistical monitoring during the trial 11

2.2.1 Randomisation 11

2.2.2 Adequacy of blinding 12

2.2.3 Sample size 12

2.2.4 Non-compliance 12

Section 3: Datasets used from the PACKMAN database 14

3.1 Intention to treat 14

3.2 Analysis datasets 14

Section 4: Analysis of the primary outcome and the Estimand Framework for PACKMAN (in relation to the primary outcome) 15

4.1 Primary outcome and the Estimand framework 15

4.2 Primary outcome (summaries and statistical analysis) 16

4.3 Primary outcome (sensitivity analyses) 17

4.4 Primary outcome (imputation analysis) 18

Section 5: Secondary Outcomes and other data collection 20

5.1 Secondary Outcomes and data collection 20

5.2 Tertiary variables and data collection 22

5.3 Patient Characteristics 23
5.4 Analysis consideration for secondary outcomes/tertiary variables 24
5.4.1 Summary statistics 24
5.4.2 Analysis strategies 24
5.5 Sub-groups Analysis 28
5.6 Secondary analysis 29
Section 6: Introduction to Health Economic Analysis 30
6.1 Purpose of the health economic analysis plan 30
6.2 Type of economic evaluation 30
6.3 Perspective 30
6.4 Time Horizon 30
6.5 Discounting 30
6.6 Intention to treat 30
6.7 Missing data 30
Section 7: Health Economic Outcomes 31
7.1 Primary health economic outcome 31
7.1.1 Quality adjusted life years (QALYs) 31
7.2 Resource Use and Costing 32
7.2.1 Direct intervention resource use and costs 32
7.2.2 Healthcare and social care resource use 32
7.2.3 Wider costs 33
Section 8: Health Economic Analysis 35
8.1 Descriptive analysis 35
8.2 Addressing missing data with multiple imputation 35
8.3 Single end point analysis: incremental costs and incremental QALYs 35
8.4 Cost-effectiveness analysis and characterising uncertainty 35
8.4.1 Characterizing uncertainty for decision makers 36
8.4.2. Sensitivity analyses 36
8.5 Decision modelling 36
8.6 Value of information analysis 37

1. ADMINISTRATIVE INFORMATION

Title: Paramedic Analgesia Comparing Ketamine and MorphiNe in trauma: PACKMaN

EudraCT Number: 2020-000154-10

IRAS Number: 266748 / 1003404

ISRCTN Number: ISRCTN14124474

CPMS Portfolio ID: 46938

SAP Version: 2*.0, 15/02/2024*

Roles and responsibility:

Signatures of:

**Role**	**Name**	**Date**	**Signature**
Author of SAP	Felix Michelet	14/06/2023	
Senior statistician	Ranjit Lall	14.06.2023	
Chief Investigator	Gavin Perkins	8/6/23	
Chief investigator	Mike Smyth	13/06/2023	
DMC Chair	Siobhan Creanor	25/05/2023	
Health Economist	Kamran Khan	23/05/2023	

Senior Health Economist	Stavros Petrou	07/06/2023	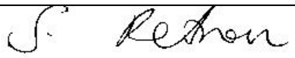

Section 1: Summary of PACKMAN

1.1 General principles for the primary statistical and health economic analysis

Given the trial is funded by the National Institute for Health Research, we will adopt principles that best meet the requirements of United Kingdom decision makers. The methods of economic evaluation will therefore be guided by the National Institute for Health and Care Excellence (NICE) guide to the methods of technology appraisal[1].

1.2 Trial Design

This is a multi-centre, randomised, double blinded trial comparing the clinical and cost-effectiveness of ketamine and morphine for severe pain in acute traumatic injury. It is a pragmatic, phase III trial working with two large NHS ambulance trusts with an internal pilot. Participants will be followed up for 6 months. Patients will be divided into each arm with an allocation ratio of 1:1, receiving either morphine or ketamine.

The treatment intervention, ketamine, will be supplied in ampoules containing 15mg in 1ml. The control intervention, morphine, will be supplied in ampoules containing 10mg in 1 ml. The ampoules will be labelled as trial related investigational medicinal product (IMP) and will be identical regardless of whether it contains ketamine or morphine. This is so that the paramedic is not able to identify which treatment they are administering.

1.3 Objectives

In the PACKMaN study, the study objective will be to test the hypothesis that paramedic administered ketamine provides more effective pain relief than morphine, for patients reporting severe pain following trauma. The Sum of Pain Intensity Difference (SPID) assessed using a 0-10 numeric rating scale and time of observation will be measured to compare morphine to ketamine.

The secondary objectives of the PACKMaN trial are to assess the effects of paramedic administered ketamine or morphine on overall pain relief / patient experience, tolerability, resource used, longer term outcomes and cost effectiveness.

1.4 Target population

Patients that meet the following criteria are the desired population for analysis:

Inclusion criteria

1. Age ≥16
2. Patient reports a pain score ≥7/10 on a 0-10 NRS following acute traumatic injury
3. Intravenous (IV) or intraosseous (IO) access obtained
4. Determined by a paramedic to require IV morphine or equivalent

Exclusion criteria

1. Known or suspected pregnancy
2. Unable to articulate severity of pain using the 0-10 NRS
3. Lack of capacity due to a reason other than pain
4. Intravenous or intraosseous (IV/IO) ketamine or opioid analgesia immediately prior to randomisation*
5. Known contraindication to either ketamine or morphine as per the SmPC**
6. Patient declines participation
7. Known prisoner

* This criterion is intended to exclude only those patients' administered ketamine or opioids via IV/IO route immediately prior to randomisation.

**SmPC is the abbreviation for Summary of Product Characteristics

1.5 Outcome measures

Primary Outcome

Effectiveness of pain relief from randomisation to arrival at hospital as measured by Sum of Pain Intensity Difference (SPID) score (using a 0-10 numerical rating scale)

Secondary Outcomes

Effectiveness of pain relief and overall patient experience from randomisation to arrival at hospital

- Total Pain Relief (TOTPAR) score
- Time to perceptible analgesia
- Time to meaningful analgesia
- Time to peak analgesia
- Duration of analgesia
- Requirement for rescue analgesia
- Proportion of patients with a pain intensity score below 4/10 (0-10 numerical rating scale (NRS)) on arrival at hospital
- Vital signs (oxygen saturation, blood pressure, heart rate, respiration rate, Glasgow Coma Scale)
- Patient Global Impression of Change on arrival at hospital

Incidence of side effects and adverse events

- Airway: vomiting, aspiration, advanced airway management
- Respiratory: desaturation, need for ventilatory support
- Cardiovascular: arrhythmia, hypotension and hypertension
- Neurologic: sedation, excitatory movements, adverse behavioural reactions

- Other: nausea, allergic reaction

Resource use

- Ambulance job cycle time (scene arrival to arrival at hospital)
- Number of ambulance resources (technicians, paramedics, doctors and vehicles) in attendance
- Cumulative IMP doses administered
- CT scan use
- Hospital or ICU admission
- Length of stay ED, ICU, Hospital

Longer term outcomes

- Chronic pain using BPI-SF at 3 & 6 months from randomisation
- Health-related quality of life EQ-5D-5L and CSRI at 3 and 6 months from randomisation
- Cost-effectiveness expressed in terms of incremental cost per quality-adjusted life year (QALY) gained using EQ-5D 5L and CSRI (at 3- and 6-months post randomisation)

Safety

Patient adverse events listed in the secondary outcomes will be summarised and reported, these however will be exempt from reporting unless deemed serious, as they will be collected on the case report form. Other adverse events which are not related to the acute traumatic injury or are complications resulting from the IMP administration to 30 days post IMP will be reported to the PACKMaN Trial team as soon as possible and within 24 hours of the research staff becoming aware of the event.

Any change of condition or other follow-up information should be sent to the PACKMaN Trial team as soon as it is available or at least within 24 hours of the information becoming available. Events will be followed up until the event has resolved or an outcome has been reached.

The SAEs will be assessed for causality from treatment and the relationship will be classed as unrelated, unlikely to be related, possible relationship, probable relationship, or definitely related.

Section 2: Monitoring of the PACKMAN Trial (operational and statistical)

2.1 Operational monitoring

2.1.1 Recruitment by ambulance service

Two ambulance trusts (services) have been selected to randomise patients.

Recruitment by ambulance service is summarised in Tables 1 and 2. These tables illustrate the balance of randomisation across the services and the number of patients recruited, with number of patients recruited per month.

2.1.2 Recruitment of patients

A consort diagram showing the flow chart of patients recruited in the trial is illustrated in figure 1.

- A detailed diagram showing the flow chart of patients from recruitment to hospital discharge will be illustrated in figure 2
- Reasons for non-enrolment will be detailed in tables 3, 4a and 4b
- A participant flow table will be included for the trial in table 5. In this diagram participant deaths and withdrawals will be summarised between hospital arrival and 3 month follow up. Between 3 month and 6 month follow up and after having reached 6 months follow up we will summarise participants death and withdrawals still, as well as those who declined consent during the follow up rate, those lost to follow and non-respondents.

2.1.2 Withdrawals & follow up rates

Withdrawals will be summarised using frequencies and percentages (Table 6).

There are two main levels of withdrawal: (a) withdrawal from treatment; (b) withdrawal from study.

For withdrawal from study, this may occur: (a) prior to hospital arrival; (b) from hospital arrival to discharge; (c) from discharge to 3 months; (d) from 3 months to 6 months.

The reasons for withdrawal will be noted and presented in Listing 1.

Cumulative withdrawals and deaths are summarised in tables 6 and 7.

2.1.3 Protocol violations and deviations

Protocol violations, with number of patients (and percentage in each arm) will be tabulated as in Table 8a and Table 8b.

Protocol deviators, with number of patients (and percentage in each arm) will also be presented as in Table 9a and Table 9b.

For each of these, we will present the number of patients who have at least one violation/deviation and the total number of violations/deviations. We will further present the details of these by each treatment arm.

2.1.4 Safety data

Serious adverse events and adverse events, with number of patients with at least one event (and percentage in each arm) and the total number of events will be tabulated as in Table 10 and Table 11 respectively.

Listings 2 and 3 details the description of the serious adverse event and adverse events by treatment arm.

2.1.5 Unblinding

Unblinding requests will be summarised by treatment arm in table 12. In this table we will detail the ambulance site that has requested the unblinding, as well as the date and further details on the unblinding.

2.1.6 Paramedic experience

We will also look at the trial paramedics and their experiences, but this will not form part of the main reporting of the trial and analysis.

Trial trained paramedics and participant recruits will be summarised by paramedic experience level (NQP vs band 6 paramedic) in table 13. We will also summarise the number of incidences of serious adverse events & non-compliances by paramedic experience level in frequencies and percentages. These will also be looked at by treatment arm in table 14, table 15, and table 16.

2.2 Statistical monitoring during the trial

2.2.1 Randomisation

Randomisation will be achieved by way of specially prepared, sequentially numbered treatment packs containing identical ampoules of either morphine (comparator) or ketamine (intervention). The content of the drug packs will be determined from a randomisation list prepared by the study programmer. The blinded block randomisation system will look to ensure a ratio of 1:1 control: intervention. The balance between arms at each site is handled by the ordering system that ensures a pre specified number of paired packs are delivered to each site. The block size is determined by the number of drugs in any given site batch order. Distribution of trial drug packs by the trial drug manufacturer will ensure equal proportions of morphine (comparator) and ketamine (intervention) are distributed to each participating site. Allocation will be concealed from study personnel, ambulance staff and patients.

Numbered study drug packs in a pre-randomised sequence, will be carried by participating ambulance paramedics. Randomisation will be achieved by opening the pack, the packs will not necessarily be opened in sequential order. Due to the method of randomisation, it will not be possible to stratify the randomisation. However, we will examine how factors such as, age, gender and use of alternative parenteral analgesia are distributed across treatment arms.

2.2.2 Adequacy of blinding

To determine the adequacy of the blinding procedure some trial paramedics have been selected to attempt to guess which treatment has been given. The trial statistician randomly selected 16 packs per site ensuring a balance across treatment arms. If one of these packs is used for a recruit, the research paramedic will get in touch with the recruiting paramedic to ask them to guess what IMP they think they administer and the reasons why they think the IMP they guessed may be the correct one. This will be done after randomisation and pain score data collection. The results from this procedure will be summarised in table 17.

2.2.3 Sample size

For the sample size calculation, we have used the Sum of Pain Intensity Difference (SPID) as the primary outcome measure. Our sample size is calculated to detect a 1-point difference (0-10 NRS) in the primary outcome (SPID) between morphine and ketamine. Other randomised controlled trials comparing ketamine and morphine have adopted a standard deviation of 3.0[2-5]), while our review of previous prehospital analgesia studies showed an average withdrawal rate of 14%[2,6-9]. Therefore, assuming a standard deviation of 3.0, with a clinically relevant difference of 1 point, 1:1 randomisation, a power of 90%, significance level of 5%, and withdrawal/non-response rate of 15%, we require a sample size of 446 patients.

To ensure the required sample size is achieved, we will monitor post-randomisation exclusions, withdrawals and non-responders and assess their impact on the required sample size/detectable effect size.

2.2.4 Non-compliance

IMP dosage compliance will be monitored. Both morphine and ketamine are controlled drugs and therefore correct IMP dosing will be important. Data Collection Form CRF02 is used to retrospectively calculate the IMP dose given based on the patient's weight and to ensure that the correct dose timing was used. Guidance on volumes and administration times are provided in the PACKMaN protocol. We will monitor that no patient received > 20ml or the patient's weight was <50kg to check the weight adjusted dose was appropriate. We will monitor any instances of an overdose or underdose.

An overdose will be defined as:

- More than 20ml IMP administered
- Further doses of IMP administered despite a pain score of 0
- IMP administered too rapidly (>10mls in first 10 mins and/or first 10mls given in less than 5 mins as this exceeds the protocol dosing schedule) in conjunction with the presence of adverse events and/or the use of midazolam/naloxone

An underdose will be defined as:

- Pain score at hospital arrival is 7 or more and the full 20ml of IMP has not been administered, with no reasonable justifications
- IMP administration period exceeds 60 mins, with no reasonable justifications provided.

As PACKMaN is a pragmatic trial, it is acknowledged that the timings of IMP dose administration will be varied given real life trauma situations and this will therefore not impact data integrity. As such, dosing times above and below the 5 mins interval stated in the protocol (for guidance purposes), are expected. IMP administration over an extended period of time is also expected. Therefore, in cases where IMP is deemed to have been given too rapidly or it is not possible to determine the IMP administration intervals or overall period, the occurrence of adverse events and/or the use of midazolam/naloxone will be used by the TMG to determine whether the event is a non-compliance. These 2 parameters will also be used to assess whether an event is a deviation, violation, serious breach or none of these.

Section 3: Datasets used from the PACKMAN database

3.1 Intention to treat

All analysis will be based on '**Intention-to-treat' (ITT),** this will include all patients randomised.

An ITT analysis produces estimates as would be in the `real world' and maintains the baseline comparability achieved by the randomisation process.

<u>The point of randomisation is defined as the time when drug pack is opened.</u> It may be that we will have a small number of patients who are found to be ineligible before any trial drug was administered, but the pack was opened. Using Fergusson et al. (2002), criteria, we will determine if a patient needs to be excluded from the ITT population[10]. For these post-randomisation exclusions, we will assess their baseline and any other data collected (that they have agreed to) to ensure that this group is similar to the study (ITT) population and no bias has occurred in excluding these patients.

The patients will be assessed for eligibility by the paramedic upon arrival to scene. Eligible patients will be informed by the attending paramedic that they are eligible to participate in the PACKMaN study and that paramedic intends to enrol them in the study unless they prefer to receive usual treatment (morphine). Informed written consent will be obtained by research paramedics after the patient is admitted to the Emergency Department. All patients that are eligible will be analysed according to the treatment arm they were randomised to, irrespective of the treatment they received. All patients with sufficient data to calculate the primary outcome will be included in the primary analysis, from baseline, and will be included in the secondary analysis provided they offer consent/complete follow up questionnaires. Participant safety data and serious adverse event data will use the same ITT population as the primary outcome.

3.2 Analysis datasets

Observed dataset

This will comprise of all the data observed (including follow up) with missing values.

Imputed dataset

See section 4.4.

Section 4: Analysis of the primary outcome and the Estimand Framework for PACKMAN (in relation to the primary outcome)

4.1 Primary outcome and the Estimand framework

In line with the ICH E9 (R1) addendum on Estimand and sensitivity analyses in clinical trials, the following defines the Estimand framework, in relation to the primary outcome.[11]

Estimand attribute	Description
Objective	To assess the effectiveness of paramedic administered Ketamine (intervention) with Morphine (standard care) for pain relief in patients reporting severe trauma, from randomisation to arrival at hospital
Treatment conditions	Pain resulting from acute trauma injury in an emergency care setting
Population	Adults (≥ 16 years) participants, identified by the attending paramedic (pre-hospital), where an intravenous (IV) or intraosseous (IO) access can be obtained
Variable (outcome)	Sum of Pain Intensity Difference (SPID) score (using a 0-10 numerical rating scale), from randomisation to arrival at hospital.<br><br>Variables used for SPID are the patients pain score and the time at which the observation was taken.<br><br>The point of randomisation is defined as the time when drug pack is opened. Arrival at hospital is when the vehicle arrives at the ED department, not when the participant is admitted to ED.
Summary measure	The SPID is measured using a weighted sum of the scores, as shown below:<br><br>$SPID_n = \sum_{i-1}^{n} (T_i - T_{i-1}) * PID_i$<br><br>$T_i$ is the time in hours when observation *i* is taken. PID_i is the difference in Pain Intensity (PI) scores from initial pain score to the pain score at time T_i. The SPID looks to calculate the area under the curve of pain intensity difference over time, using the trapezoidal rule. The summary statistics will be the mean (SD), together with the 95% confidence interval of the SPID for all the patients in the study.<br><br>The SPID will be summarised by treatment arms.
Handling Intercurrent events	Post-randomisation events which may affect the interpretation or occurrence of the primary outcome include:

	ICE 1: discontinuation of the allocated treatment (i.e., withdrawal from treatment not study)<br><br>ICE 2: use of rescue medication<br><br>ICE 3: non-compliance: over-dosing, or under-dosing<br><br>ICE 4: death during period from initial IMP admin to hospital arrival
Strategies for handling intercurrent events	ICE 1:<br>(a) *Treatment policy* (same as main analysis) - analysis as observed because it is unlikely that patients will be withdrawn, or treatment discontinued. Treatment discontinuation is defined as either participant is withdrawn from treatment without reason, or participant dies before hospital arrival, or participant has an adverse event that results in treatment stopping.<br>ICE 2:<br>(a) *Treatment policy* (same as main analysis) - i.e., analyse as observed data. Use of rescue medication is considered part of the treatment and we will assess the effect of the intervention, regardless of the event<br>ICE 3:<br>(a) *Principal stratum strategy* - assessing the effect of the intervention, having adjusted for the non-compliance (i.e., over-dosing/under-dosing); see below for details of analysis.<br>ICE 4:<br>(a) *Composite strategy:* using death as a composite with the primary outcome (see below for details of analysis).

Treatment policy will result in the primary analysis

4.2 Primary outcome (summaries and statistical analysis)

Primary outcome data will be summarised with descriptive statistics (n, mean, standard deviation, median, interquartile range, and n of missing data). We anticipate low levels of missing data for the primary outcome at the end of the trial.

Baseline pain score will be calculated as the latest available pain measurement before initial IMP dose was administered, in the unlikely event that this baseline score is 0, the data will be classed as missing.

Results of all the statistical analysis using statistical modelling will be presented using mean (sd) and two-sided 95% confidence intervals. The primary analysis will be carried out using linear regression on an ITT population and on the observed data with adjustments for the following covariates as fixed effects:

- ambulance service
- age (<60; $\geq$60 years)
- gender (male, female, transgender, other, not disclosed)
- administration of intravenous paracetamol prior to randomisation (yes, no)
- weight (cutpoint on the mean/median)

Ambulance service is included as a covariate as there may be some differences in practice across sites. Age and gender are included as covariates as the groups specified can experience pain differently. Administration of IV paracetamol prior to randomisation is included as a covariate as it is an adjunctive treatment that may impact pain response. Weight is included as a covariate since different weight groups have different requirement for an adequate dose of IMP.

The secondary analysis will be the unadjusted analysis.

Model assumptions will be checked visually, for example through the use of residual plots and if necessary, hypothesis tests will be used (i.e the Anderson Darling test for checking if errors are normally distributed).

For heavily skewed data, where the standard deviation is larger than the mean, we will assess methods will accommodate for the non-normality in the data (e.g. Gamma distribution models or transformation of the data), instead of linear regression models. Descriptive statistics and results will be presented with adjusted and unadjusted models in tables 18 and 19. Hypothesis tests will be conducted at a two-sided 5% significance level and their corresponding p-value will be reported. There is no adjustment for multiple testing for the PACKMaN trial.

Graphical display of participant pain score differences over time/observations will be explored for summary of the primary outcome.

4.3 Primary outcome (sensitivity analyses)

The sensitivity analyses for the primary outcome will be those described above in section 4.1, around the estimand framework.[11] All the statistical analysis using statistical modelling will be presented using mean (sd) and 95% confidence intervals.

__Intercurrent event 1 (Treatment policy):__

In the case of discontinuation of treatment we will analyse the data based on the ITT population specified in section 3.1.

__Intercurrent event 2 (Treatment policy):__

In the event of rescue analgesia being used we will analyse the data based on the ITT population specified in section 3.1.

Intercurrent event 3 (Composite strategy):

The effect of compliance will be assessed using a complier average causal effect (CACE) analysis[12,13] will be conducted for the primary outcome and mortality. A structural mean model with the inclusion of an instrumental variable will be fitted to estimate the treatment effect among those who complied with the study drug infusion protocol. Results will be presented in tables 20-28.

Intercurrent event 4 (Composite strategy):

The death rate prior to completion of data collected to calculate the primary outcome is expected to be low. If there are more than 5% death affecting assessment of the primary outcome across all participants, we will use the Pocock's win-ratio method[14]. In the Pocock's win-ratio, each patient from the intervention group is compared to each patient in the standard care group (a total of *m x n* comparisons where *m* is the total number of patients in the intervention group and *n* the number of patients in the standard care group) for the mortality endpoint and then on the primary outcome. Based on which patient performs better in each pair, the group they belong to will be declared the 'winner'. This will give us the total number of winners in each group and our test statistic is based on this. In the case of the win-ratio method, the statistic is the number of winners in the intervention group divided by the number of winners in the standard care group. This approach allows us to infer if the intervention is significantly better than the standard care having considered the clinical priority i.e., treating mortality as a more important outcome than having a better primary outcome.

4.4 Primary outcome (imputation analysis)

Some item missingness for the primary outcome is expected, however if 2 or more pain scores are recorded for a participant the primary outcome will still be able to be calculated. Due to the nature of the data it will be difficult to judge when an item is genuinely missing or when it is clinically reasonable for a participating paramedic to not record participant pain scores. Multiple imputation is not recommended if data is missing not at random (MNAR).[15] Imputation methods were considered by the statisticians along with the trial management team and it was deemed that last observation carried forward was not suitable due to the variability of pain scores for participants. Clinically, there are also reasons for why a paramedic may not have recorded a pain score so we cannot assume that data are missing at random, therefore multiple imputation is also not possible.

Additionally, the primary outcome is calculated as the area under the curve using the pain scores of the participant, therefore imputing missing items will not improve the measurement of the primary outcome.

Section 5: Secondary Outcomes and other data collection

5.1 Secondary Outcomes and data collection

Secondary Outcomes	Definition	Mitigations
The Total Pain Relief (TOTPAR)	The TOTPAR is measured using a weighted sum of the scores, as shown below: $$TOTPAR_n = \sum_{i-1}^{n} (T_i - T_{i-1}) * PAR_i$$ T_i is the time in hours when observation i is taken. PAR_i is the pain relief score measured as defined below on a scale of 1 to 3. We measure minimal pain relief as 1.8 (1.7-1.9) change in pain score, much pain relief as 4.0 (3.9-4.1) and very much pain relief as 5.2 (5-5.4)[16]. Percentage changes defined as 20.3 (19-21.6), 44.4 (43.2–45.6), 56.1 (53.9–58.4) respectively[16].	
Time to Perceptible Analgesia	Perceptible pain relief is defined as a 20% improvement in NRS pain score from the initial pain score[16]. The time will be taken as time of perceptible analgesia minus time of first administration.	If 20% improvement not achieved, we will score this as perceptible analgesia not achieved.
Time to Meaningful Analgesia	Defined as a 44% improvement in NRS pain score from initial pain score[16]. The time will be taken as time of meaningful analgesia minus time of first administration.	If 44% improvement not achieved, we will score this as meaningful analgesia not achieved.
Time to Peak Analgesia	Measured as the time when lowest NRS pain score, relative to initial pain score, is	

	achieved minus time of first administration.	
Duration of Analgesia	Measured as the time period in which patient pain scores have consecutively decreased or remained stationary.	
Requirement for Rescue Analgesia	We will record whether a patient has needed rescue analgesia after randomisation and before arrival at hospital. We will also consider if any adjunctive analgesia was administered (e.g. Entonox, paracetamol, ibuprofen, or other).	
Proportion of patients with pain intensity score below 4/10 on NRS scale	At hospital arrival, as defined previously, the research paramedic will record a NRS pain score. We will provide the proportion, as a percentage, of each patient that achieved a score < 4/10.	If there is no hospital arrival score, we will use the last available score recorded provided it is not the baseline pain score.
Vital Signs	At each observation time, the respiratory rate (bpm), oxygen saturations (%), heart rate (bpm), blood pressure (mmHg) and their Glasgow Coma Scale (GCS).	
Glasgow Coma Scale	Three subscales measuring eyes, verbal, and motor response of each patient. The scales are as such:<br>Eyes 1-4<br>Verbal 1-5<br>Motor 1-6	
Global Impression of Change	Using a 7-point Likert scale. The options offered ranging from 'very much improved' to 'very much worse'	
Side effects and adverse events	Measured in the following categories, 'Airway', 'Respiratory', 'Cardiovascular', 'Neurologic', and 'other'.	
Ambulance job cycle time	Time taken from arrival on scene to hospital arrival	
Number of ambulance resources	Number of doctors, paramedics, doctors, and vehicles attending scene	

Cumulative IMP doses administered	Total dose of IMP administered	
CT scan use	If patient had a CT scan and how many	
Hospital or ICU admission	Yes; no option if patient is admitted to hospital or ICU	
Length of stay in ED, ICU, or hospital	Classed as date and time of admission to date and time of discharge	
**BPI-SF at 3 and 6 Months**		
9 part self-reported form which allows us to monitor the severity of the patient's pain and its effect on their daily life. Split into two sections, pain intensity and pain inference.	9 part self-reported form which allows us to monitor the severity of the patient's pain and its effect on their daily life.	
Pain Severity	The pain severity part assesses the pain of the patient at its worst, least, average and now.	We can then determine the average of the 4 categories to determine pain intensity, however it is recommended that we present all 4 of the options, worst, least, average, and now.
Pain Interference	The interference section measures the effect of pain in 7 different tasks, walking, work, mood, enjoyment of life, relations with others, and sleep.	Measure as a mean if at least 4 of the sections have been completed.

Descriptive statistics and results will be presented with adjusted and unadjusted models for the secondary outcomes in tables 29-43.

5.2 Tertiary variables and data collection

Variable	Timepoint	Measure
Time to first noticeable pain relief	Transport to hospital	Time from initial IMP dose to participant has first noticeable pain relief
Time to adequate pain relief	Transport to hospital	Time from initial IMP dose to participant has adequate pain relief
Pain score at hospital arrival	Transport to hospital	Pain score collected at hospital arrival
Mechanism of injury	Transport to hospital	Blunt trauma; Penetrating trauma; Burn

Injuries sustained	Transport to hospital	Facture/dislocation; Soft tissue injury; Wound/laceration
Body part/region injured	Transport to hospital	Head; Neck; Check & back; Abdomen; Pelvis; Upper limbs; Lower limbs
Analgesia post randomisation	Transport to hospital	Yes; no option with specific options offered: Entonox; Paracetamol; Ibuprofen; Other with time analgesia was given.
Midazolam/Naloxone administered	Transport to hospital	Yes; no option with time and dose given.
IMP admin	Transport to hospital	Timing and route of dose given as well as amount given in mg.
Discharge location	Hospital data collection	Normal residence; Rehabilitation service; Another acute hospital; Death in hospital
Participant entered into TARN network	Hospital data collection	Yes; no
Participant suffered IMP underdose	Data is collected during transport to hospital if underdose is suspected a report will be made to the clinicians within trial team to determine if underdose has occurred	Yes; no
Participant suffered IMP overdose	Data is collected during transport to hospital if overdose is suspected a report will be made to the clinicians within trial team to determine if overdose has occurred	Yes; no

5.3 Patient Characteristics

The characteristics of enrolled patients will be summarised by treatment arm. Below is a table of the patient characteristics collected and how they are measured. Patient characteristics will be summarised in table 44, table 45, and table 46.

Baseline characteristic	Characteristic measure
Age	Years
Weight	Kilograms
Gender, Ethnicity	Tick boxes offered (Discrete data)
Ambulance Service	Patients will be enrolled either by the West Midlands or Yorkshire ambulance service
Inclusion criteria	Inclusion criteria data will be collected and recorded by the paramedic
Mechanism of injury	The mechanism of injury (blunt trauma, penetrating trauma, burn), as well as the injury sustained (fracture/dislocation, soft tissue injury, wound/laceration), and body part/region of injury.
Vital signs	Initial participant vital signs (respiratory rate, oxygen sats, heart rate, blood pressure systolic, blood pressure diastolic, Glasgow coma scale).

5.4 Analysis consideration for secondary outcomes/tertiary variables

5.4.1 Summary statistics

In general, continuous baseline and outcome data will be summarised with descriptive statistics, including n, mean, standard deviation, median, interquartile range and n of missing data. Categorical baseline and outcome data will be summarised with frequency counts and percentages. All the statistical analysis using statistical modelling will be presented using mean (sd) and two-sided 95% confidence intervals. In addition, some graphical presentations will be considered for some variables.

Kaplan-Meier plots will be considered for time to events, as well dose response curves and other graphical displays to identify an effective IMP dose.

5.4.2 Analysis strategies

Sum of pain intensity difference (SPID)

Sum of pain intensity difference data will be treated as continuous. Sum of pain intensity difference will be summarised and analysed using a linear regression model on the ITT. Analysis will be carried

out with and without adjustment for covariates as detailed in the primary outcome sections, with results presented in table 18 and 19.

Total pain relief (TOTPAR)

Total pain relief data will be treated as continuous. Total pain relief will be summarised and analysed using a linear regression model on the ITT. Analysis will be carried out with and without adjustment for covariates as detailed in the primary outcome sections, with results presented in table 29 and 30.

Time to perceptible analgesia

Time to perceptible analgesia will be summarised as mean, standard deviation, median, and interquartile range (IQR). We will analyse time to perceptible analgesia using time to event analysis. Analysis on the ITT population using Cox's proportional hazard model with and without adjustment for covariates as detailed in the primary outcome section, with results presented in table 29 and 30. Observations that will be censored will include those where time to perceptible analgesia has not been reached, as defined in the above table. This will include patients who withdraw or died (and have not reached perceptible analgesia) and those who at time of initial IMP administration to time of hospital arrival still have not reached perceptible analgesia.

Time to meaningful analgesia

Time to meaningful analgesia will be analysed in the same way as detailed for time to perceptible analgesia, with results presented in table 29 and 30. Censored observations will be similarly defined as for time to perceptible analgesia.

Time to peak analgesia

Time to peak analgesia will be analysed in the same way as detailed for time to perceptible analgesia, with results presented in table 29 and 30. Censored observations will be similarly defined as for time to perceptible analgesia.

Duration of analgesia

Duration of analgesia will be analysed in the same way as detailed for time to perceptible analgesia, with results presented in table 29 and 30. Censored observations will be similarly defined as for time to perceptible analgesia.

Requirement for rescue analgesia

Requirement for rescue analgesia will be treated as dichotomous. Requirement for rescue analgesia will be summarised and analysed using an ordinary logistic regression model on the ITT population. Rescue analgesia will be defined by the administration of open label ketamine or morphine before hospital arrival. We will also consider requirement for adjunctive analgesia (post randomisation entonox, paracetamol, ibuprofen). Analysis will be carried out with and without adjustment for covariates as detailed in the primary outcome section, with results presented in table 29 and 30.

Proportion of patients with pain intensity score below 4/10 on NRS scale

Proportion of patients with pain intensity score below 4/10 on NRS scale will be analysed in the same way as detailed for requirement for rescue analgesia, with results presented in table 29 and 30.

Global impression of change

Global impression of change will be treated as ordinal data. Unadjusted and adjusted analysis of the global impression of change will be summarised and analysed using a proportional odds model on the ITT population. If the assumptions for a proportional odds model do not hold, nonparametric tests will be used to compare global impression of change across treatment arms, for example the Mann-Witney U test. We will look to dichotomise the global impression of change in the case where the proportional odds for the ordinal model does not satisfy for the adjusted analysis. Adjusted analysis will use the covariates as detailed in the primary outcome section, with results presented in table 29 and 30.

Side effects and adverse events

Side effects and adverse events will be analysed in the same way as detailed for requirement for rescue analgesia, with results presented in table 34 and 35.

Vital signs

Participant vital signs and Glasgow coma scale are collected in a longitudinal data format, where observations are not collected at specific timepoints This means that vital signs observations are collected at irregular timepoints and different frequencies across participants.

Different methods have been explored to summarise and analyse these data. Observations from the same participant are not assumed to be random, therefore the randomness in the data will come from the different participants' set of observations. The most appropriate model for these data is the mixed effects model[17] without a random intercept where the residual errors correlation structure accounts for the dependency within participant observations.

An unstructured correlation structure was first explored, as this allows for the variance and covariance for each observation to be distinctly calculated, however this model specification did not converge unless some participant observations were truncated. An auto regressive structure of order 1 has therefore been adopted, as this structure assumes a lag between observations[17]. Here the observations that are closer to each other have a higher correlation than those further apart and will account for the varying timespan between observations across the participants.

Analysis will be carried out with and without adjustment for the covariates detailed in the primary outcome section, with results as presented in table 32 and 33.

Glasgow coma scale

Glasgow coma scale will be analysed in the same as detailed for vital signs, with results presented in table 32 and 33.

Brief pain inventory

The brief pain inventory score will be analysed in the same as detailed for total pain relief, with results presented in tables 40-43.

Ambulance job cycle time

Ambulance job cycle time will be summarised as mean, standard deviation, median, and interquartile range (IQR). We will assess the distribution of time ambulance job cycle time. If this is normally distributed, then we will use linear regression models, otherwise will be examine the difference using non-parametric statistics or gamma- distribution models on the ITT population with and without adjustment for covariates as detailed in the primary outcome section, with results presented in table 36 and 37.

Number of ambulance clinicians in attendance

Number of ambulance clinicians in attendance will be analysed in the same way as detailed for ambulance job cycle time, with results presented in table 36 and 37. Ambulance clinicians includes paramedics and technicians.

Cumulative IMP doses administered

Cumulative IMP doses administered will be analysed in the same way as detailed for ambulance job cycle time, with results presented in table 36 and 37.

CT scan use

CT scan use will be analysed in the same way as detailed for requirement for rescue analgesia, with results presented in table 38 and 39.

Number of CT scans used

Number of CT scans used will be analysed in the same way as detailed for ambulance job cycle time, with results presented in table 38 and 39.

Hospital or ICU admission

Hospital or ICU admission will be analysed in the same way as detailed for requirement for rescue analgesia, with results presented in table 38 and 39.

Length of stay in ED, ICU, hospital

Length of stay in ED, ICU, hospital will be analysed in the same way as detailed for ambulance job cycle time, with results presented in table 38 and 39.

5.5 Sub-groups Analysis

Subgroup analysis will be conducted for the following:

- Age (<60 and ≥60 years)
- Gender (male; female)
- Alternative parental analgesia prior to randomisation (participant received intravenous paracetamol, yes; no)

These subgroup analyses will be performed on the ITT population. The primary outcome will be used as the dependent variable and interaction with treatment. Linear regression models will be used to assess the subgroup effect, using interaction terms, subgroup by treatment, to measure the effect of each subgroup. As these analyses are post-hoc analyses which are not powered for any effect size, emphasis will not be based on the statistical testing, rather the point estimates and two-sided 95% confidence intervals, the results will be summarised in table 48.

Injury severity score, determined by the participant being entered to the TARN network was initially considered as a subgroup analysis. However, there would be insufficient data to complete the subgroup analysis.

5.6 Secondary analysis

In addition to the standard frequentist analysis that was originally planned at the start of the trial, we will also perform a Bayesian analysis, to aid the interpretation of the results. If the assumptions used in the trial design are found to be incorrect, for example the observed standard deviation of the primary outcome is higher than expected, the accuracy of the trial results may be reduced, which would lower the chance of a treatment effect reaching the threshold for statistical significance. In this situation, interpretation of the results by clinicians and other decision makers will be helped by producing a quantitative summary of the probability that ketamine is a superior analgesia than morphine for acute pain, considering existing evidence and the trial's results through Bayesian analysis.

The analysis will model the primary outcome, SPID, using Bayesian linear regression models, and including the same covariates as the main analysis. The means of the posterior distribution for each of the covariates and treatment group will be calculated from this model. Sensitivity analysis will determine the effects of the prior, this is expected to be small due to the large amount of data available from the trial.

Section 6: Introduction to Health Economic Analysis

6.1 Purpose of the health economic analysis plan

The objective of the health economics analysis is to inform decision makers regarding the cost-effectiveness of paramedic administered ketamine compared to morphine for the management of acute severe pain from traumatic injury. The purpose of the health economics analysis plan (HEAP) is to outline the framework of methods that will be used to analyse the health economic components of the trial to ensure the integrity of the cost-effectiveness analysis.

6.2 Type of economic evaluation

As recommended, the primary health economic analysis will be a cost-utility analysis with incremental quality adjusted life years (QALYs) as the primary health economic outcome[1]. Following NICE guidance, the EQ-5D-5L will be used for the construction of QALYs (see section 7.1.1)[1].

6.3 Perspective

A healthcare and personal social services (PSS) will be adopted in the primary analysis as recommended by NICE[1].

6.4 Time Horizon

The primary health economic analysis will run concurrently to the effectiveness analysis. The EQ-5D-5L will be collected at three and six-months post-randomisation. The time horizon will therefore be the 6-month period post-randomisation. Should outcomes not have converged after 6 months, we will consider the development of a decision analytic model to extrapolate the cost-effectiveness results over a lifetime horizon (see section 8.5).

6.5 Discounting

Given the trial-based analysis has a time-horizon of 6 months, costs and QALYs will not be discounted. Should longer-term decision modelling be conducted, we will use the 3.5% annual discount rate as recommended by NICE to discount future costs and QALYs[1].

6.6 Intention to treat

The health economic analysis will adopt the principle of 'intention to treat'[18]. This means that the health economic analysis will analyse individuals according to the trial arms to which they were randomised.

6.7 Missing data

Missing data is a common occurrence within randomised clinical trials and needs to be considered within the health economic analysis[19]. Missing data will be explored, and if non-trivial (5% or more in either costs or QALYs)[20], the base case analysis will use multiple imputation (MI)[21] as the preferred method for estimating results in the presence of missing data. MI uses the observed data and samples from the predictive distribution to create multiple datasets[22]. Under the assumption of missing at random, this provides unbiased estimates; this allows uncertainty surrounding estimates to be maintained whilst allowing full use of the available data (see section 8.2).

Section 7: Health Economic Outcomes

7.1 Primary health economic outcome

As recommended by NICE, incremental quality adjusted life years (QALYs) will be used as the primary measure of health consequence for the health economic analysis[1].

7.1.1 Quality adjusted life years (QALYs)

Estimating QALYs

QALYs combine both mortality and morbidity into a single measure that can be compared across contexts within the healthcare service. To calculate QALYs it is necessary to combine a preference-based health-related quality of life outcomes with survival benefits. In this study, we are using the EQ-5D-5L[23] at two time points (3 months, 6 months). The EQ-5D-5L is a preference-based measure of health-related quality of life and is recommended by NICE for use in economic evaluation[1]. The measure contains a descriptive system with five dimensions of health, each containing five levels. There exist value-sets[24,25] that allow the calculation of *utility* scores for any given set of responses to the EQ-5D-5L descriptive system. A utility score is a score on a cardinal scale indexed at zero and one, where zero represents death, and one represents full health (negative states are possible). These utility values can be combined with survival benefits to derive QALYs. Although a new UK specific EQ-5D-5L value set exists[24], it has been a subject of controversy[26]. Currently NICE instead recommends[27] the use of the Van Hout et al[25] 'cross-walk' algorithm.

A challenge to the analysis is the lack of baseline EQ-5D-5L measurement which is required for the calculation of QALYs for the duration of follow-up from randomisation. For ethical, logistical and pragmatic reasons, it is not possible to capture baseline EQ-5D-5L measurements in patients suffering acute pain following trauma within this trial. This is not uncommon within trials involving emergency and critical care settings[28]. Ideally, the EQ-5D-5L would be completed at the time of randomisation or as soon as possible afterwards. This however is not possible in this trial. A systematic review of emergency and critical care studies[28] identified four strategies that have been used in such situations. First, the most common approach (57% of all studies identified by the systematic review) is to assign a fixed health utility to all participants at baseline. Second, some studies (29%) estimated QALYs using only the available data and implicitly ignored any benefits that occurred before the first follow up data collection point. One study (7%) asked patients to retrospectively recall their health state at randomisation. Finally, one study (7%) mapped health states onto EQ-5D-3L using expert evidence to derive baseline health states. The primary analysis will use a fixed baseline approach for all participants. This fixed value will be derived by mapping the 'typical' acute pain trauma case to the EQ-5D-5L using expert opinion. The sensitivity of this assumption will be tested within sensitivity analyses (see 8.4.2). Sensitivity analyses will include assigning different values to patients according to severity as determined by registration to the Trauma Audit and Research Network (TARN). TARN can be used as proxy for severity as the most serious trauma patients will be registered onto TARN whilst less severe cases will not (non-TARN). We will then use expert opinion to estimate a baseline EQ-5D profile for both TARN and non-TARN patients.

QALYs for each patient will be calculated by using the utility values at baseline, 3 months and 6 months. QALYs will be calculated by linearly interpolating utility values at the three time points and

calculating the area under the curve using the trapezium rule[29]. QALYs will be calculated for each patient in the trial.

7.2 Resource Use and Costing

To calculate costs for use in cost-utility analysis it is necessary to capture information on resources used for both the control and intervention arm. Costs within this trial can be split into the following broad components:

- Direct intervention costs (medication costs)
- Direct healthcare and PSS costs (e.g. medication for side-effects, outpatient appointments, community care)
- Other societal costs (e.g. value of lost productivity, out of pocket expenses)

NICE's guide to methods of technology appraisal recommend costing from an NHS and personal social services (PSS) perspective[1]. The primary analysis will only consider the first two categories of costs; broader societal costs will be included within a sensitivity analysis. To calculate costs, it is first necessary to capture resource use, and then apply unit costs to each resource input. The price year for the analysis will be informed by the latest available base year for common costing resources at time of analysis.

7.2.1 Direct intervention resource use and costs

The PACKMaN trial focuses on administration of two alternative medications for pain relief in patients with severe pain. The intervention arm will receive ketamine hydrochloride whilst the control arm will receive morphine sulphate. The intervention components and associated resource use are summarised within the table below. This table shows what the components are, how they will be collected and where unit cost sources may be sourced from.

Direct intervention resource use and cost sources

Intervention arm			
**Resource type**	**Resource use**	**How collected**	**Unit costs source**
Ketamine hydrochloride	Number and ml of doses administered.	Recorded within ambulance service data	NHSBSA[29]
Control arm			
Morphine sulphate	Number and ml of doses administered.	Recorded within ambulance service data	NHSBSA[29]

7.2.2 Healthcare and social care resource use

In according with NICE guidance, we will capture healthcare and PSS costs for both arms of the trial[1]. This will include, within-ambulance costs, inpatient care, outpatient care, community care, accident and emergency admission, medication, and personal social services. The methods for capturing the resource use and the sources for unit costs are outlined in the table below. Within ambulance costs will be captured through the ambulance service data form, index admission costs will be collected

via the hospital data collection form, whilst the remaining costs will be collected through the case report forms at 3 and 6 months.

Health and social care costs for both arms

Resource type	Resource use	How collected	Unit cost sources
Within ambulance – rescue analgesia medication	Entonox, paracetamol, ibuprofen, other painkiller	Ambulance service data form	NHSBSA[30]
Within ambulance – side-effects medication	Midazolam or naloxone	Ambulance service data form	NHSBSA[30]
Inpatient care – index admission	Length of stay and number of days at each level of care	Hospital data collection form	NHS Reference Costs[31]
Index admission	CT scans	Hospital data collection form	NHS Reference Costs[31]
Inpatient care – follow up	Specified within CRFs	CRFs at 3m and 6m	NHS Reference Costs[31] and PSSRU[32]. HRG4+ 'Code to Group'[33] used to allocate inpatient care to HRG groups for costing.
Outpatient care	Specified within CRFs	CRFs at 3m and 6m	NHS Reference Costs[31] and PSSRU[32].
Community care	Specified within CRFs	CRFs at 3m and 6m	NHS Reference Costs[31] and PSSRU[32].
Medication	Specified within CRFs	CRFs at 3m and 6m	NHSBSA[30]
Personal social services	Specified within CRFs	CRFs at 3m and 6m	PSSRU[32] unit costs

7.2.3 Wider costs

Within an additional sensitivity analysis, we will also be collecting information related to days lost from work and out of pocket expenses.

Wider costs

Resource type	Resource use	How collected	Unit cost sources
Absence from work and out of pocket expenses	Specified within CRFs	CRFs at 3m and 6m	Stated in CRFs

Section 8: Health Economic Analysis

8.1 Descriptive analysis

Resource use, costs and EQ-5D utility scores will first be presented descriptively to inform parameters for future health economic studies (this includes means and standard deviations). Costs will be calculated for all perspectives outlined previously.

8.2 Addressing missing data with multiple imputation

If the proportion of missing data for either costs or QALYs is more than 5% we will use multiple imputation to impute data within the base-case analysis. This data will then be used in the incremental analysis of costs, QALYs and the joint cost-effectiveness analysis. A complete case analysis will be included as a sensitivity analysis. Stata[34] will be used to conduct both the multiple imputation and the analysis of imputed data. The 'mi impute chained' command which uses chained equations to generate imputed datasets will be used for each treatment group. Within the imputation regression framework, we will include both costs and EQ-5D-5L at each timepoint as both imputed and predictor variables. We will also include any auxiliary variables that are found to be highly correlated ($r>0.4$)[35] with costs or EQ-5D-5L, or are believed to be associated with missingness. We will use predictive mean matching drawing from the 5 nearest 'neighbours', this is important for the avoidance of drawing implausible values, e.g., utility values over 1, and 'negative costs'. The number of iterations will be guided by the fraction of missing information[20]. We will then use the 'mi estimate' functionality within Stata to run the analyses (specified in subsequent sections) within each dataset and to combine results using Rubin's combination rule to allow inferential statistics. Imputations will be added until estimates stabilise. We will examine the validity of the imputed data by comparing the distribution of the imputations and observed data both visually and statistically.

8.3 Single end point analysis: incremental costs and incremental QALYs

Before conducting the joint cost-effectiveness analysis, we will examine the impact of the intervention on incremental costs and incremental QALYs in isolation. Differences between the two arms will be assessed using a regression framework. The exact specification will depend upon the nature of the data. Costs will be estimated by combining resource use data with unit costs. Costs for each patient within the trial will be calculated and incremental costs between the two arms will be estimated. Again, a regression framework will be used, and its exact specification will be informed by the nature and distribution of the data.

8.4 Cost-effectiveness analysis and characterising uncertainty

We will use bivariate regression analysis in the form of seemingly unrelated regressions (with bootstrapping) for the joint analysis of costs and QALYs. This framework offers several benefits: first of all it accounts for the existence of correlation between costs and outcomes for patients; second it allows the inclusion of covariates within the analysis, this is particularly relevant for the adjustment of baseline utility with respect to QALYs accrued; third it is generally robust to non-normal distributions; fourth, it can account for clustering either by including clusters as a fixed effect or by running the seemingly unrelated regressions in a multi-level framework. Non-parametric bootstrapping will be used to examine the level of uncertainty by presenting the bootstrapped results on a cost-effectiveness plane, and by generating cost-effectiveness acceptability curves (CEACs). Should there be distributional or computational concerns (e.g. difficulty in fitting a multi-

level seemingly unrelated regression model with imputed data in Stata) then we may consider combining costs and outcomes within a univariate net-benefit regression framework.

8.4.1 Characterizing uncertainty for decision makers

Cost-effectiveness acceptability curves (CEACs) will be used to characterise uncertainty. CEACs show the probability that the intervention is cost-effective compared to the control at different levels of willingness to pay for QALYs and explicitly highlight the uncertainty within the decision problem. To avoid the issues related to uncertainty around cost-effectiveness ratios we will calculate net-monetary benefit for each of the bootstrapped iterations:

$$\Delta NB = \Delta e\gamma - \Delta c$$

In this instance, ΔNB refers to the incremental net monetary benefit, Δe reflects the incremental outcome of interest, incremental QALYs, whilst Δc refers to the incremental costs. The symbol γ refers to the decision maker's willingness to pay per QALY. For each of the bootstrapped cost-effectiveness samples we will calculate the associated net-monetary benefit across a range of levels of willingness to pay (γ). For each γ the proportion of iterations where net-benefit is greater than zero can be used estimate the probability that the intervention is more cost-effective at that willingness to pay. This will be conducted for a range of γ including £20,000 and £30,000 per QALY as specified by NICE and plotted to derive a CEAC[1].

8.4.2. Sensitivity analyses

In addition to the probabilistic sensitivity analysis outlined above we will also consider sensitivity analyses, these will include:

- Costing from a societal perspective
- Complete case analysis (assuming missing data exceeds 5%)
- Changing baseline utility assumptions
- Subgroup analyses as specified within the statistical analysis plan
- Cost per sum of pain intensity difference (SPID) score point reduction

8.5 Decision modelling

The primary trial-based analysis will focus on the costs and QALYs accrued during the trial period. There however is potential for costs and benefits to accrue beyond the trial period. If outcomes have not converged by the 6m timepoint we will consider extrapolating the results over a longer time horizon using a decision analytic model. This would involve combining the trial data with external sources to estimate the long-term cost-effectiveness of the intervention. Any costs and benefits accruing after the first year would be discounted at a rate of 3.5% per year and full probabilistic sensitivity analysis would be conducted in line with the NICE reference case[1]. A decision as to the necessity of building a decision analytic model and its specification will be made following discussion between the health economists and the trial team following preliminary analysis of the data. This will be informed by considerations such as the conclusiveness and direction of within trial results. For example, if the control dominates the intervention and extrapolation would only increase the strength of this result then there is little need to extrapolate further as the intervention should be rejected.

8.6 Value of information analysis

Should a decision model be developed we will also conduct a value of information (VoI) analysis to examine the expected value of future research. The VoI analysis will entail the calculation of the expected value of perfect information (EVPI) using data from the cost-effectiveness analysis. EVPI can be conceptualised as the expected gain from eliminating uncertainty within the decision problem, or put another way, the expected loss associated with uncertainty. This is essentially the probability of the decision being wrong multiplied by the average consequence of being wrong[36]. This allows us to calculate the estimated value of 'perfect knowledge' which is the maximum value society should be willing to pay for additional evidence to reduce uncertainty around whether the intervention or the control is more cost-effective[37]. Using the trial data, we will calculate the per person EVPI using a willingness to pay threshold of £30,000 per QALY, this representing the threshold NICE uses in practice[38]. This will be multiplied by the number of potential beneficiaries of the intervention within the NHS along with the technological horizon (years) to estimate population EVPI. Discounting of EVPI will be applied at 3.5% beyond the first year.

References

1. NICE, *Guide to the methods of technology appraisal 2013*. NICE, 2013. Accessed: Jul. 28, 2014. [Online]. Available: https://www.nice.org.uk/article/PMG9/chapter/5-The-reference-case
2. Le Cornec C, Lariby S, Brenckmann V, Hardouin JB, Ecoffey C, Le Pottier M, Fradin P, Broch H, Kabbaj A, Auffret Y, Deciron F. Is intravenously administered, subdissociative-dose KETAmine non-inferior to MORPHine for prehospital analgesia (the KETAMORPH study): study protocol for a randomized controlled trial. Trials. 2018 Dec;19(1):1-6.
3. Motov S, Rockoff B, Cohen V, Pushkar I, Likourezos A, McKay C, Soleyman-Zomalan E, Homel P, Terentiev V, Fromm C. Intravenous subdissociative-dose ketamine versus morphine for analgesia in the emergency department: a randomized controlled trial. Annals of emergency medicine. 2015 Sep 1;66(3):222-9.
4. Motov S, Mann S, Drapkin J, Butt M, Likourezos A, Yetter E, Brady J, Rothberger N, Gohel A, Flom P, Mai M. Intravenous subdissociative-dose ketamine versus morphine for acute geriatric pain in the emergency department: a randomized controlled trial. The American Journal of Emergency Medicine. 2019 Feb 1;37(2):220-7.
5. Sin B, Tatunchak T, Paryavi M, Olivo M, Mian U, Ruiz J, Shah B, de Souza S. The use of ketamine for acute treatment of pain: a randomized, double-blind, placebo-controlled trial. The Journal of emergency medicine. 2017 May 1;52(5):601-8.
6. Tran KP, Nguyen Q, Truong XN, Le V, Le VP, Mai N, Husum H, Losvik OK. A comparison of ketamine and morphine analgesia in prehospital trauma care: a cluster randomized clinical trial in rural Quang Tri province, Vietnam. Prehospital Emergency Care. 2014 Apr 3;18(2):257-64.
7. Jennings PA, Cameron P, Bernard S, Walker T, Jolley D, Fitzgerald M, Masci K. Morphine and ketamine is superior to morphine alone for out-of-hospital trauma analgesia: a randomized controlled trial. Annals of emergency medicine. 2012 Jun 1;59(6):497-503.
8. Mahshidfar B, Mofidi M, Fattahi M, Farsi D, Moghadam PH, Abbasi S, Rezai M. Acute pain management in emergency department, low dose ketamine versus morphine, a randomized clinical trial. Anesthesiology and pain medicine. 2017 Dec;7(6).
9. Beaudoin FL, Lin C, Guan W, Merchant RC. Low-dose ketamine improves pain relief in patients receiving intravenous opioids for acute pain in the emergency department: results of a randomized, double-blind, clinical trial. Academic Emergency Medicine. 2014 Nov;21(11):1193-202.
10. Fergusson D, Aaron SD, Guyatt G, Hébert P. Post-randomisation exclusions: the intention to treat principle and excluding patients from analysis. BMJ. 2002 Sep 21;325(7365):652-4. doi: 10.1136/bmj.325.7365.652. PMID: 12242181; PMCID: PMC1124168
11. International Council for Harmonisation of Technical Requirements for Pharmaceuticals For Human Use. ICH Harmonised Guideline.Addendum on estimands and sensitivity analysis in clinical trials to the guideline on statistical principles for clinical trials E9(R1). 2017.https://database.ich.org/sites/default/files/E9-R1_Step4_Guideline_2019_1203.pdf
12. Knox, Christopher R., Lall, Ranjit, Hansen, Zara and Lamb, Sarah E.. (2014) Treatment compliance and effectiveness of a cognitive behavioural intervention for low back pain :a complier average causal effect approach to the BeST data set. BMC Musculoskeletal Disorders, Volume 15 . Article number 17. ISSN 1471-2474

13. Kasza J. Research Note: Estimating the complier average causal effect when participants in randomised trials depart from allocated treatment. J Physiother. 2021 Apr;67(2):147-149. doi: 10.1016/j.jphys.2021.02.002. Epub 2021 Feb 20. PMID: 33622650.

14. Pocock SJ, Ariti CA, Collier TJ, et al. The win ratio: a new approach to the analysis of composite endpoints in clinical trials based on clinical priorities. *European Heart Journal.* 2011;33(2):176-182.

15. Carpenter, J.R. and M.G. Kenward, Missing data in randomised controlled trials : a practical guide. 2007.

16. Cepeda, Soledad M.a; Africano, Juan Ma; Polo, Rodolfoa; Alcala, Ramiroa; Carr, Daniel Bb,c,*. What decline in pain intensity is meaningful to patients with acute pain?. Pain 105(1):p 151-157, September 2003. | DOI: 10.1016/S0304-3959(03)00176-3

17. Funatogawa, I., Funatogawa, T. (2018). Autoregressive Linear Mixed Effects Models. In: Longitudinal Data Analysis. SpringerBriefs in Statistics(). Springer, Singapore. https://doi.org/10.1007/978-981-10-0077-5_2

18. S. Ramsey *et al.*, 'Cost-Effectiveness Analysis Alongside Clinical Trials II - An ISPOR Good Research Practives Task Force Report', *Value Health*, vol. 18, pp. 161–172, 2015, doi: 10.1016/j.jval.2015.02.001.

19. M. Gomes, K. Díaz-Ordaz, R. Grieve, and M. G. Kenward, 'Multiple imputation methods for handling missing data in cost-effectiveness analyses that use data from hierarchical studies: an application to cluster randomized trials.', *Med. Decis. Mak. Int. J. Soc. Med. Decis. Mak.*, vol. 33, no. 8, pp. 1051–63, Nov. 2013, doi: 10.1177/0272989X13492203.

20. P. Madley-Dowd, R. Hughes, K. Tilling, and J. Heron, 'The proportion of missing data should not be used to guide decisions on multiple imputation', *J. Clin. Epidemiol.*, vol. 110, pp. 63–73, Jun. 2019, doi: 10.1016/j.jclinepi.2019.02.016.

21. J. L. Schafer, 'Multiple imputation: a primer':, *Stat. Methods Med. Res.*, Jul. 2016, doi: 10.1177/096228029900800102.

22. J. A. C. Sterne *et al.*, 'Multiple imputation for missing data in epidemiological and clinical research: potential and pitfalls.', *BMJ*, vol. 338, no. jun29_1, p. b2393, Jan. 2009, doi: 10.1136/bmj.b2393.

23. M. Herdman *et al.*, 'Development and preliminary testing of the new five-level version of EQ-5D (EQ-5D-5L)', *Qual. Life Res.*, vol. 20, no. 10, pp. 1727–1736, Dec. 2011, doi: 10.1007/s11136-011-9903-x.

24. N. Devlin, K. Shah, Y. Feng, B. Mulhern, and B. van Hout, 'Valuing health-related quality of life : An EQ-5D-5L value set for England', *OHE Res. Pap.*, vol. January, no. January, 2016, [Online]. Available: https://www.ohe.org/publications/valuing-health-related-quality-life-eq-5d-5l-value-set-england

25. B. van Hout *et al.*, 'Interim scoring for the EQ-5D-5L: mapping the EQ-5D-5L to EQ-5D-3L value sets.', *Value Health*, vol. 15, no. 5, pp. 708–715, 2012.

26. C. Sampson, 'Bad reasons not to use the EQ-5D-5L', *The Academic Health Economists' Blog*, Mar. 23, 2018. https://aheblog.com/2018/03/23/bad-reasons-not-to-use-the-eq-5d-5l/ (accessed Apr. 29, 2020).

27. NICE, 'Position statement on use of the EQ-5D-5L value set for England (updated October 2019) | NICE technology appraisal guidance | NICE guidance | Our programmes | What we do | About', *NICE*, 2019. https://www.nice.org.uk/about/what-we-do/our-programmes/nice-guidance/technology-appraisal-guidance/eq-5d-5l (accessed Apr. 29, 2020).

28. M. Dritsaki, F. Achana, J. Mason, and S. Petrou, 'Methodological Issues Surrounding the Use of Baseline Health-Related Quality of Life Data to Inform Trial-Based Economic Evaluations of Interventions Within Emergency and Critical Care Settings: A Systematic Literature Review', *PharmacoEconomics*, vol. 35, no. 5, pp. 501–515, May 2017, doi: 10.1007/s40273-016-0485-x.

29. A. Manca, N. Hawkins, and M. J. Sculpher, 'Estimating mean QALYs in trial-based cost-effectiveness analysis: the importance of controlling for baseline utility.', *Health Econ.*, vol. 14, no. 5, pp. 487–96, May 2005, doi: 10.1002/hec.944.

30. 'Prescription Cost Analysis (PCA) data | NHSBSA'. https://www.nhsbsa.nhs.uk/prescription-data/dispensing-data/prescription-cost-analysis-pca-data (accessed May 24, 2021).

31. Department of Health, 'NHS reference costs 2014 to 2015', London, 2015.

32. 'Unit Costs of Health and Social Care 2017 | PSSRU'. https://www.pssru.ac.uk/project-pages/unit-costs/unit-costs-2017/ (accessed Oct. 14, 2019).

33. 'HRG4+ 2020/21 Local Payment Grouper', *NHS Digital*. https://digital.nhs.uk/services/national-casemix-office/downloads-groupers-and-tools/local-payment-2020-21 (accessed May 14, 2020).

34. StataCorp, *Stata statistical software: Release 16*. College Station, TX: StataCorp LLC, 2019.

35. UCLA IDRE, 'Multiple Imputation in Stata'.

36. E. C. F. Wilson, 'A Practical Guide to Value of Information Analysis', *PharmacoEconomics*, vol. 33, no. 2, pp. 105–121, Feb. 2015, doi: 10.1007/s40273-014-0219-x.

37. H. V. Eeren, S. J. Schawo, R. H. J. Scholte, J. J. V. Busschbach, and L. Hakkaart, 'Value of Information Analysis Applied to the Economic Evaluation of Interventions Aimed at Reducing Juvenile Delinquency: An Illustration', *PLOS ONE*, vol. 10, no. 7, p. e0131255, Jul. 2015, doi: 10.1371/journal.pone.0131255.

38. K. Claxton *et al.*, 'Methods for the estimation of the National Institute for Health and Care Excellence cost-effectiveness threshold', *Health Technol. Assess. Winch. Engl.*, vol. 19, no. 14, pp. 1–503, v–vi, Feb. 2015, doi: 10.3310/hta19140.

Figure 1: CONSORT diagram

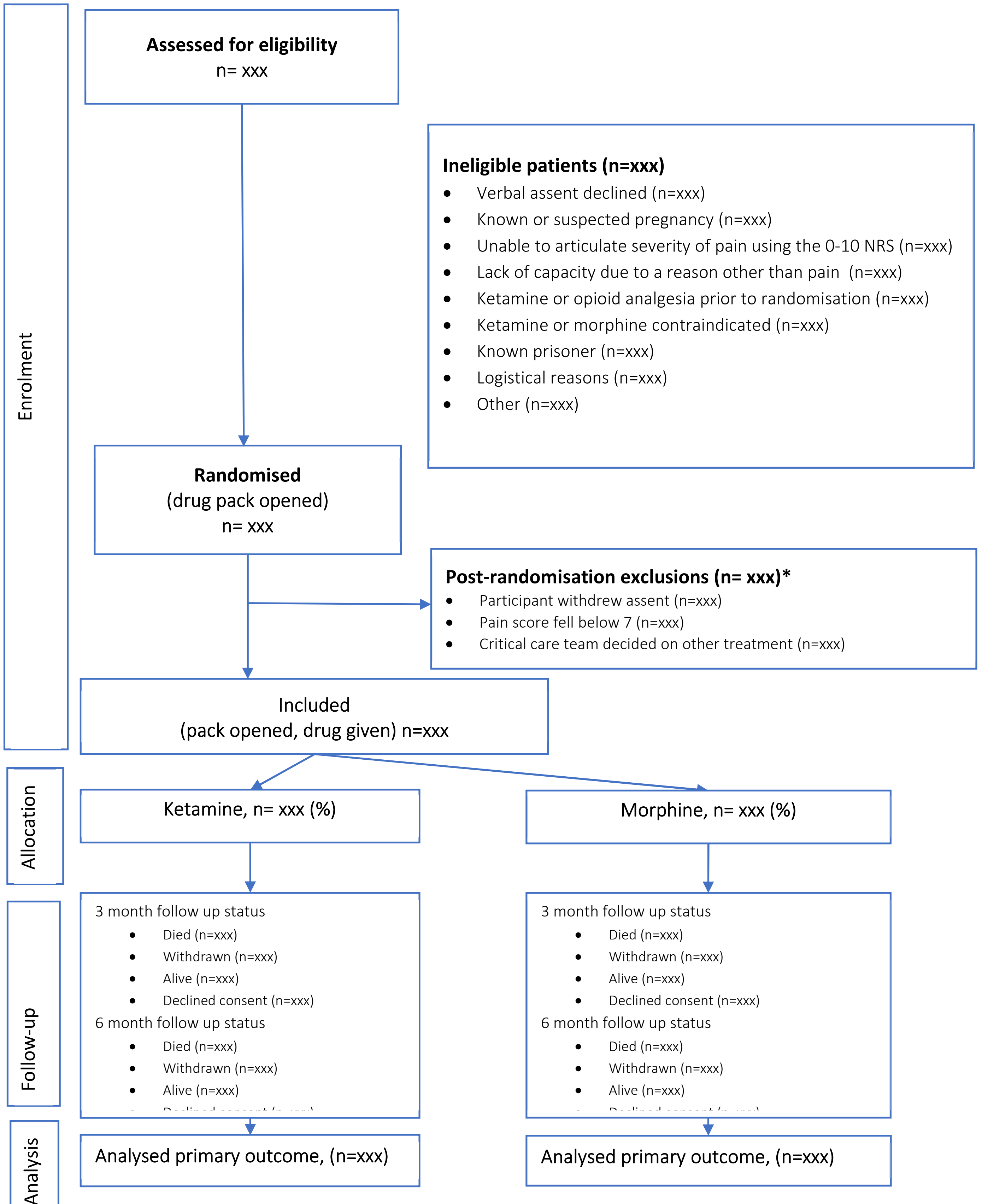

*screened by trial trained paramedic

Figure 2: Patient flow of PACKMaN trial from recruitment to hospital discharge

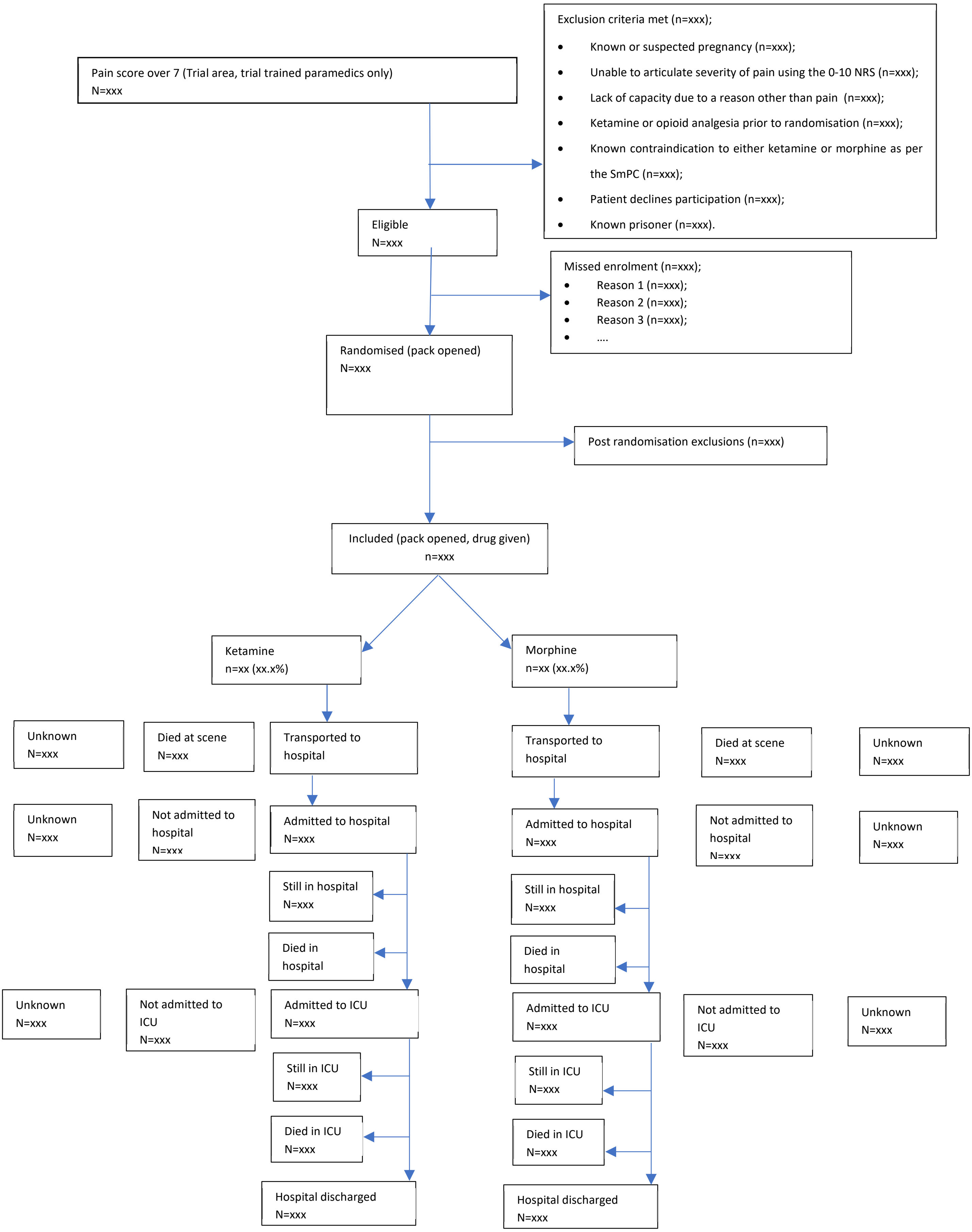

Table 1: Randomised patients by treatment and ambulance service – assessment of balance of randomisation

	Ketamine	Morphine	Total
Ambulance service 1	N(%)	N(%)	N
Ambulance service 2	N(%)	N(%)	N
TOTAL	N(%)	N(%)	N

Percentages are based within each treatment arm

Table 2: Current recruitment by treatment and ambulance service

	Number of months recruitment	Average number of patients recruited per month	Ketamine	Morphine	Total
SITE	N	N	N(%)	N(%)	N
Ambulance service 1	N	N	N(%)	N(%)	N
Ambulance service 2	N	N	N(%)	N(%)	N
TOTAL	N	N	N	N	N

Percentages are based within each treatment arm

Table 3: Reasons for missed enrolment, per ambulance service and total

Missed enrolment reasons	Ambulance service 1	Ambulance service 2	Total
Total			
Paramedic trained but not carrying pack	N(%)	N(%)	N(%)
Paramedic trained but pack used previously on shift	N(%)	N(%)	N(%)
Other reason	N(%)	N(%)	N(%)

Percentages are based within each ambulance service

Table 4a: Reasons for ineligibility, per ambulance service and total

Eligibility Criteria	Ambulance service 1	Ambulance service 2	Total
Total			
Under 16 years of age	N(%)	N(%)	N(%)
Patient reported pain score is below 7	N(%)	N(%)	N(%)
Intravenous and intraosseous access not obtained	N(%)	N(%)	N(%)
Paramedic determined patient did not require IV morphine or equivalent	N(%)	N(%)	N(%)
Known or suspected pregnancy	N(%)	N(%)	N(%)
Unable to articulate pain severity	N(%)	N(%)	N(%)
Lack of capacity due to reason other than pain	N(%)	N(%)	N(%)
Ketamine or opioid analgesia prior to randomisation	N(%)	N(%)	N(%)
Known contraindication to either ketamine or morphine, per SmPC	N(%)	N(%)	N(%)

Patient declined participation	N(%)	N(%)	N(%)
Known prisoner	N(%)	N(%)	N(%)
Other	N(%)	N(%)	N(%)

Percentages are based within each ambulance service

Table 4b: Post randomisation exclusion reasons

TNO	Date of randomisation	Reason for no primary outcome data

Table 5: Participant trial flow of all randomised participants by treatment arm

Time period	Patient flow	Ketamine	Morphine	Total
At randomisation	TOTAL participants RANDOMISED	Xxx (xx.x%)	Xxx (xx.x%)	xxx
From randomisation to arrival in hospital	Alive with data collection	Xxx (xx.x%)	Xxx (xx.x%)	xxx
	Deaths	Xxx (xx.x%)	Xxx (xx.x%)	xxx
	Withdrawals from trial treatment (but continued trial participations)	Xxx (xx.x%)	Xxx (xx.x%)	xxx
	Withdrawal from study (after consent)	Xxx (xx.x%)	Xxx (xx.x%)	xxx
	Did not consent	Xxx (xx.x%)	Xxx (xx.x%)	xxx
	Lost to follow-up	Xxx (xx.x%)	Xxx (xx.x%)	xxx
From arrival in hospital to discharge	Alive with data collection	Xxx (xx.x%)	Xxx (xx.x%)	xxx
	Deaths	Xxx (xx.x%)	Xxx (xx.x%)	xxx
	Withdrawal from study (after consent)	Xxx (xx.x%)	Xxx (xx.x%)	xxx
	Did not consent	Xxx (xx.x%)	Xxx (xx.x%)	xxx
	Lost to follow-up	Xxx (xx.x%)	Xxx (xx.x%)	xxx
From discharge to 3-month follow-up	Alive with data collection	Xxx (xx.x%)	Xxx (xx.x%)	xxx
	Deaths	Xxx (xx.x%)	Xxx (xx.x%)	xxx
	Withdrawal from study (after consent)	Xxx (xx.x%)	Xxx (xx.x%)	xxx
	Did not consent	Xxx (xx.x%)	Xxx (xx.x%)	xxx
	Lost to follow-up	Xxx (xx.x%)	Xxx (xx.x%)	xxx
	Alive with data collection	Xxx (xx.x%)	Xxx (xx.x%)	xxx

From discharge to 6-month follow-up	Deaths	Xxx (xx.x%)	Xxx (xx.x%)	xxx
	Withdrawal from study (after consent)	Xxx (xx.x%)	Xxx (xx.x%)	xxx
	Did not consent	Xxx (xx.x%)	Xxx (xx.x%)	xxx
	Lost to follow-up	Xxx (xx.x%)	Xxx (xx.x%)	xxx

Percentages are based within each column

Table 6: Withdrawals by treatment arm

Time period	Type of Withdrawals	Ketamine	Morphine	Total
Randomisation to hospital arrival	Withdrawn from treatment	Xxx (xx.x%)	Xxx (xx.x%)	xxx
Randomisation to hospital arrival	Withdrawn from study	Xxx (xx.x%)	Xxx (xx.x%)	xxx
Randomisation to discharge	Withdrawn from study	Xxx (xx.x%)	Xxx (xx.x%)	xxx
Randomisation to 3 months	Withdrawn from study	Xxx (xx.x%)	Xxx (xx.x%)	xxx
Randomisation to 6 months	Withdrawn from study	Xxx (xx.x%)	Xxx (xx.x%)	xxx
Total		Xxx	Xxx	xxx

Percentages are based within each column

Listing 1 reasons for withdrawal

Table 7: Deaths by treatment arm

Time period	Ketamine	Morphine	Total
Randomisation to hospital arrival	N(%)	N(%)	N
Randomisation to hospital arrival	N(%)	N(%)	N
Randomisation to discharge	N(%)	N(%)	N
Randomisation to 3 months	N(%)	N(%)	N
Randomisation to 6 months	N(%)	N(%)	N
Total	N	N	N

Percentages are based within each column

Table 8a: Summary of Protocol violations by treatment arm

PROTOCOL VIOLATIONS	Ketamine	Morphine	Total
No of patients with at least one protocol violations	N(%)	N(%)	N
Number of protocol violations	N(%)	N(%)	N
TOTAL	N	N	N

Percentages are based within each column

Table 8b: Details of Protocol violations by treatment arm

Ketamine

CAPA Number	TNO	Issue	Date aware	Date resolved/actions implemented	File note/ deviation/ violation/ breach
	XX				
	XX				

Morphine

CAPA Number	TNO	Issue	Date aware	Date resolved/actions implemented	File note/ deviation/ violation/ breach
	XX				
	XX				

Table 9a: Summary of Protocol deviations by treatment arm

PROTOCOL DEVIATIONS	Ketamine	Morphine	Total
No of patients with at least one protocol violations	N(%)	N(%)	N
Number of protocol violations	N(%)	N(%)	N
TOTAL	N	N	N

Percentages are based within each column

Table 9b: Details of Protocol deviations by treatment arm

Ketamine

CAPA Number	TNO	Issue	Date aware	Date resolved/actions implemented	File note/ deviation/ violation/ breach
	XX				
	XX				

Morphine

CAPA Number	TNO	Issue	Date aware	Date resolved/actions implemented	File note/ deviation/ violation/ breach
	XX				
	XX				

Table 10: Number of Serious adverse events

SERIOUS ADVERSE EVENTS	Ketamine	Morphine	Unadjusted estimate (95% CI); p-value	Total
No of patients with at least one SAE	N(%)	N(%)		N
Number of SAE	N(%)	N(%)		N
TOTAL	N	N		N

Percentages are based within each column

LISTING 2: Serious adverse events by treatment arm

Table 11: Number of Adverse events

ADVERSE EVENTS	Ketamine	Morphine	Unadjusted estimate (95% CI); p-value	Total
No of patients with at least one AE	N(%)	N(%)		N
Number of AE	N(%)	N(%)		N
TOTAL	N	N		N

Percentages are based within each column

LISTING 3: Adverse events by treatment arm

Table 12: Unblinding requests by treatment arm

	Ketamine	Morphine	Total
Total number of unblinding requests	Xxx (xx.x%)	Xxx (xx.x%)	xxx

Table 13: Participants recruited, and paramedics trained by paramedic experience level

Intervention: Ketamine

	Recruited	% Recruited	Trial-trained	% Trial-trained
Band 6	xxx	xx.x%	xxx	xx.x%
NQPs	xxx	xx.x%	xxx	xx.x%
No longer in service	xxx	xx.x%	xxx	xx.x%
Total	xxx	xx.x%	xxx	xx.x%
WMAS Band 6	xxx	xx.x%	xxx	xx.x%
WMAS NQPs	xxx	xx.x%	xxx	xx.x%
WMAS No longer in service	xxx	xx.x%	xxx	xx.x%
WMAS Total	xxx	xx.x%	xxx	xx.x%

YAS band 6	xxx	xx.x%	xxx	xx.x%
YAS NQPs	xxx	xx.x%	xxx	xx.x%
YAS UK - no longer in service	xxx	xx.x%	xxx	xx.x%
YAS Total	xxx	xx.x%	xxx	xx.x%

Percentages are based within each column

Intervention: Morphine

	Recruited	% Recruited	Trial-trained	% Trial-trained
Band 6	xxx	xx.x%	xxx	xx.x%
NQPs	xxx	xx.x%	xxx	xx.x%
No longer in service	xxx	xx.x%	xxx	xx.x%
Total	xxx	xx.x%	xxx	xx.x%
WMAS Band 6	xxx	xx.x%	xxx	xx.x%
WMAS NQPs	xxx	xx.x%	xxx	xx.x%
WMAS No longer in service	xxx	xx.x%	xxx	xx.x%
WMAS Total	xxx	xx.x%	xxx	xx.x%
YAS band 6	xxx	xx.x%	xxx	xx.x%
YAS NQPs	xxx	xx.x%	xxx	xx.x%
YAS UK - no longer in service	xxx	xx.x%	xxx	xx.x%
YAS Total	xxx	xx.x%	xxx	xx.x%

Percentages are based within each column

Table 14: Serious adverse events, adverse events and non-compliance frequencies for paramedic experience level

Intervention: Ketamine

	Band 6	NQP	Unadjusted estimate (95% CI); p-value
Serious Adverse Events	Xxx (xx.x%)	Xxx (xx.x%)	
Adverse Events	Xxx (xx.x%)	Xxx (xx.x%)	
Non-compliances	Xxx (xx.x%)	Xxx (xx.x%)	

Percentages are based within each column

Table 15: Non compliances details for paramedic experience level

Intervention: Ketamine

TNO	Site	NC	NC details	NC classification	Paramedic experience level (e.g. NQP1, NQP2?)	Any notes
…<br>…						

Intervention: Morphine

TNO	Site	NC	NC details	NC classification	Paramedic experience level (e.g. NQP1, NQP2?)	Any notes
…<br>…						

Table 16: Serious adverse events details for paramedic experience level

Intervention: Ketamine

TNO	Site	SAE	Causality	SAE resolved?	Paramedic experience level (e.g. NQP1, NQP2?)	Any notes

Intervention: Morphine

TNO	Site	SAE	Causality	SAE resolved?	Paramedic experience level (e.g. NQP1, NQP2?)	Any notes

Table 17: Adequacy of blinding

Paramedic guess	Ketamine	Morphine	Total	P value
Correct	Xx (xx.x%)	Xx (xx.x%)	Xx	
Incorrect	Xx (xx.x%)	Xx (xx.x%)	xx	0.xx

Percentages are based within each column

Table 18: Primary outcome summary statistics of all randomised participants by treatment arm

		Ketamine	Morphine	Total
Initial pain score at eligibility	Mean(sd)	xx.x (xx.x)	xx.x (xx.x)	xx.x (xx.x)
	Median (IQR)	xx.x (xx.x – xx.x)	xx.x (xx.x – xx.x)	xx.x (xx.x – xx.x)
	Missing (%)	N (xx%)	N (xx%)	N (xx%)
Sum of pain intensity (SPID)	Mean (sd)	xx.x (xx.x)	xx.x (xx.x)	xx.x (xx.x)
	Median (IQR)	xx.x (xx.x – xx.x)	xx.x (xx.x – xx.x)	xx.x (xx.x – xx.x)
	Missing (%)	N (xx%)	N (xx%)	N (xx%)

Percentages are based within each column

Table 19: Primary outcome study outcome at baseline, [mean(sd)]

	Ketamine	Morphine	Unadjusted estimate (95% CI); p-value	Adjusted estimate (95% CI)*; p-value
Sum of pain intensity (SPID)	(n=xxx)<br>xx.x (xx.x)	(n=xxx)<br>xx.x (xx.x)	MD, xx.x (xx.x to xx.x); 0.xx	MD, xx.x (xx.x to xx.x); 0.xx

Percentages are based within each column

Table 20: Baseline demographic characteristics of randomised participants summarised by compliance status (compliers and non-compliers)

Baseline demographics		Complier	Non-complier	Unadjusted estimate (95% CI); p-value
Age	Mean (sd)	xx.x (xx.x)	xx.x (xx.x)	
Gender	Male	N (xx%)	N (xx%)	
	Female	N (xx%)	N (xx%)	
	Transgender	N (xx%)	N (xx%)	
	Other	N (xx%)	N (xx%)	
	Not disclosed	N (xx%)	N (xx%)	
	Missing	N (xx%)	N (xx%)	
Ethnicity	White	N (xx%)	N (xx%)	
	Black	N (xx%)	N (xx%)	
	Mixed	N (xx%)	N (xx%)	
	Any other ethnic group	N (xx%)	N (xx%)	
	Asian	N (xx%)	N (xx%)	
	Ethnicity not given	N (xx%)	N (xx%)	
	Missing	N (xx%)	N (xx%)	
Weight	Mean (sd)	xx.x (xx.x)	xx.x (xx.x)	

Percentages are based within each column

Table 21: Primary outcome and mortality summary statistics of randomised participants summarised by compliance status (compliers and non-compliers), [mean (sd)]

	Complier	Non-complier	Unadjusted estimate (95% CI); p-value
Sum of pain intensity (SPID)	(n=xxx)<br>xx.x (xx.x)	(n=xxx)<br>xx.x (xx.x)	
Mortality	N (xx%)	N (xx%)	

Percentages are based within each column

Table 22: Injury characteristics of randomised participants summarised by compliance status (compliers and non-compliers)

			Complier	Non-complier	Unadjusted estimate (95% CI); p-value
Mechanism of Injury:	Blunt Trauma		N (xx%)	N (xx%)	
	Penetrating Trauma		N (xx%)	N (xx%)	
	Burn		N (xx%)	N (xx%)	
	Missing		N (xx%)	N (xx%)	
Injuries sustained:	Fracture/ dislocation	Yes	N (xx%)	N (xx%)	
		No	N (xx%)	N (xx%)	
		Missing	N (xx%)	N (xx%)	
	Soft tissue injury	Yes	N (xx%)	N (xx%)	
		No	N (xx%)	N (xx%)	
		Missing	N (xx%)	N (xx%)	
	Wound/ laceration	Yes	N (xx%)	N (xx%)	
		No	N (xx%)	N (xx%)	
		Missing	N (xx%)	N (xx%)	
Body part/region injured:	Head	Yes	N (xx%)	N (xx%)	
		No	N (xx%)	N (xx%)	
		Missing	N (xx%)	N (xx%)	
	Neck	Yes	N (xx%)	N (xx%)	
		No	N (xx%)	N (xx%)	
		Missing	N (xx%)	N (xx%)	
	Chest & back	Yes	N (xx%)	N (xx%)	
		No	N (xx%)	N (xx%)	
		Missing	N (xx%)	N (xx%)	
	Abdomen	Yes	N (xx%)	N (xx%)	
		No	N (xx%)	N (xx%)	
		Missing	N (xx%)	N (xx%)	
	Pelvis	Yes	N (xx%)	N (xx%)	
		No	N (xx%)	N (xx%)	
		Missing	N (xx%)	N (xx%)	
	Upper limbs	Yes	N (xx%)	N (xx%)	
		No	N (xx%)	N (xx%)	
		Missing	N (xx%)	N (xx%)	
	Lower limbs	Yes	N (xx%)	N (xx%)	

		No	N (xx%)	N (xx%)	
		Missing	N (xx%)	N (xx%)	

Percentages are based within each column

Table 23: Secondary outcomes summary statistics of randomised participants summarised by compliance status (compliers and non-compliers)

	Complier	Non-complier	Unadjusted estimate (95% CI); p-value
Total pain relief (TOTPAR), mean(sd)	(n=xxx)<br>xx.x (xx.x)	(n=xxx)<br>xx.x (xx.x)	
Time to perceptible analgesia, median (IQR)	(n=xxx)<br>xx.x (xx.x to xx.x)	(n=xxx)<br>xx.x (xx.x to xx.x)	
Time to meaningful analgesia, median (IQR)	(n=xxx)<br>xx.x (xx.x to xx.x)	(n=xxx)<br>xx.x (xx.x to xx.x)	
Time to peak analgesia, median (IQR)	(n=xxx)<br>xx.x (xx.x to xx.x)	(n=xxx)<br>xx.x (xx.x to xx.x)	
Duration of analgesia, median (IQR)	(n=xxx)<br>xx.x (xx.x to xx.x)	(n=xxx)<br>xx.x (xx.x to xx.x)	
Patient global impression of change, N (%)			
Very much improved	(n=xxx) xxx (xx.x%)	(n=xxx) xxx (xx.x%)	
Much improved	(n=xxx) xxx (xx.x%)	(n=xxx) xxx (xx.x%)	
Minimally improved	(n=xxx) xxx (xx.x%)	(n=xxx) xxx (xx.x%)	
No change	(n=xxx) xxx (xx.x%)	(n=xxx) xxx (xx.x%)	
Minimally worse	(n=xxx) xxx (xx.x%)	(n=xxx) xxx (xx.x%)	
Much worse	(n=xxx) xxx (xx.x%)	(n=xxx) xxx (xx.x%)	
Very much worse	(n=xxx) xxx (xx.x%)	(n=xxx) xxx (xx.x%)	
Required rescue analgesia, N (%)	(n=xxx) xxx (xx.x%)	(n=xxx) xxx (xx.x%)	

Final pain score below 4/10, N (%)	(n=xxx) xxx (xx.x%)	(n=xxx) xxx (xx.x%)	

Percentages are based within each column

Table 24: Ambulance vital signs characteristics of randomised participants summarised by compliance status (compliers and non-compliers)

	Complier	Non-complier	Unadjusted estimate (95% CI); p-value
Blood pressure Systolic (mmHg)	(n=xxx)<br>xx.x (xx.x)	(n=xxx)<br>xx.x (xx.x)	
Blood pressure Diastolic (mmHg)	(n=xxx)<br>xx.x (xx.x)	(n=xxx)<br>xx.x (xx.x)	
Pulse rate (bpm)	(n=xxx)<br>xx.x (xx.x)	(n=xxx)<br>xx.x (xx.x)	
Respiration rate (bpm)	(n=xxx)<br>xx.x (xx.x)	(n=xxx)<br>xx.x (xx.x)	
Oxygen sats (%)	(n=xxx)<br>xx.x (xx.x)	(n=xxx)<br>xx.x (xx.x)	
Glasgow coma scale (GCS)	(n=xxx)<br>xx.x (xx.x)	(n=xxx)<br>xx.x (xx.x)	

Table 25: Expected adverse event of randomised participants summarised by compliance status (compliers and non-compliers)

			Complier	Non-complier	Unadjusted estimate (95% CI); p-value
Experienced adverse event			N (xx%)	N (xx%)	
Airway	Vomiting	Yes	N (xx%)	N (xx%)	
		No	N (xx%)	N (xx%)	
		Missing	N (xx%)	N (xx%)	
	Aspiration	Yes	N (xx%)	N (xx%)	
		No	N (xx%)	N (xx%)	
		Missing	N (xx%)	N (xx%)	
	Advanced airway management	Yes	N (xx%)	N (xx%)	
		No	N (xx%)	N (xx%)	
		Missing	N (xx%)	N (xx%)	
Respiratory	Desaturation	Yes	N (xx%)	N (xx%)	

		No	N (xx%)	N (xx%)	
		Missing	N (xx%)	N (xx%)	
	Need for ventilatory support	Yes	N (xx%)	N (xx%)	
		No	N (xx%)	N (xx%)	
		Missing	N (xx%)	N (xx%)	
Cardiovascular	Arrhythmia	Yes	N (xx%)	N (xx%)	
		No	N (xx%)	N (xx%)	
		Missing	N (xx%)	N (xx%)	
	Hypotension	Yes	N (xx%)	N (xx%)	
		No	N (xx%)	N (xx%)	
		Missing	N (xx%)	N (xx%)	
	Hypertension	Yes	N (xx%)	N (xx%)	
		No	N (xx%)	N (xx%)	
		Missing	N (xx%)	N (xx%)	
Neurologic	Sedation	Yes	N (xx%)	N (xx%)	
		No	N (xx%)	N (xx%)	
		Missing	N (xx%)	N (xx%)	
	Excitatory movements	Yes	N (xx%)	N (xx%)	
		No	N (xx%)	N (xx%)	
		Missing	N (xx%)	N (xx%)	
	Adverse behavioural reactions	Yes	N (xx%)	N (xx%)	
		No	N (xx%)	N (xx%)	
		Missing	N (xx%)	N (xx%)	
Other	Allergic reaction	Yes	N (xx%)	N (xx%)	
		No	N (xx%)	N (xx%)	
		Missing	N (xx%)	N (xx%)	
	Nausea	Yes	N (xx%)	N (xx%)	
		No	N (xx%)	N (xx%)	
		Missing	N (xx%)	N (xx%)	

Percentages are based within each column

Table 26: Ambulance resource use of randomised participants summarised by compliance status (compliers and non-compliers)

	Complier	Non-complier	Unadjusted estimate (95% CI); p-value
Ambulance job cycle time, median (IQR)	(n=xxx)<br>xx.x (xx.x to xx.x)	(n=xxx)<br>xx.x (xx.x to xx.x)	
Cumulative IMP dose, mean(sd)	(n=xxx)<br>xx.x (xx.x)	(n=xxx)<br>xx.x (xx.x)	
Number of ambulance clinicans, mean(sd)	(n=xxx)<br>xx.x (xx.x)	(n=xxx)<br>xx.x (xx.x)	
Number of paramedics, mean (sd)	(n=xxx)<br>xx.x (xx.x)	(n=xxx)<br>xx.x (xx.x)	

Number of ambulance technicians/students, mean (sd)	(n=xxx)<br>xx.x (xx.x)	(n=xxx)<br>xx.x (xx.x)	
Number of doctors , mean (sd)	(n=xxx)<br>xx.x (xx.x)	(n=xxx)<br>xx.x (xx.x)	
Number of others attending, mean (sd)	(n=xxx)<br>xx.x (xx.x)	(n=xxx)<br>xx.x (xx.x)	

Percentages are based within each column

Table 27: Hospital stay and procedures of randomised participants summarised by compliance status (compliers and non-compliers)

	Complier	Non-complier	Unadjusted estimate (95% CI); p-value
Length of stay in ED, median (IQR)	(n=xxx)<br>xx.x (xx.x to xx.x)	(n=xxx)<br>xx.x (xx.x to xx.x)	
Patient admitted to hospital, N (%)	(n=xxx) xxx (xx.x%)	(n=xxx) xxx (xx.x%)	
Admitted to critical care, N (%)	(n=xxx) xxx (xx.x%)	(n=xxx) xxx (xx.x%)	
Days in receiving level 1/2 critical care, mean (sd)	(n=xxx)<br>xx.x (xx.x)	(n=xxx)<br>xx.x (xx.x)	
Days in receiving level 3 critical care, mean (sd)	(n=xxx)<br>xx.x (xx.x)	(n=xxx)<br>xx.x (xx.x)	
Days in receiving unknown level of critical care, mean (sd)	(n=xxx)<br>xx.x (xx.x)	(n=xxx)<br>xx.x (xx.x)	
Length of stay hospital, mean (sd)	(n=xxx)<br>xx.x (xx.x)	(n=xxx)<br>xx.x (xx.x)	
Participant entered to TARN, N (%)	(n=xxx) xxx (xx.x%)	(n=xxx) xxx (xx.x%)	
CT Scan use, N (%)	(n=xxx) xxx (xx.x%)	(n=xxx) xxx (xx.x%)	
Number of CT scans per patient, mean (sd)	(n=xxx)<br>xx.x (xx.x)	(n=xxx)<br>xx.x (xx.x)	

Percentages are based within each column

Table 28: Long term outcomes of randomised participants summarised by compliance status (compliers and non-compliers)

		Complier	Non-complier	Unadjusted estimate (95% CI); p-value
Pain severity	Overall	(n=xxx)<br>xx.x (xx.x)	(n=xxx)<br>xx.x (xx.x)	
	Worst pain	(n=xxx)<br>xx.x (xx.x)	(n=xxx)<br>xx.x (xx.x)	
	Least pain	(n=xxx)<br>xx.x (xx.x)	(n=xxx)<br>xx.x (xx.x)	
	Average pain	(n=xxx)<br>xx.x (xx.x)	(n=xxx)<br>xx.x (xx.x)	
	Pain now	(n=xxx)<br>xx.x (xx.x)	(n=xxx)<br>xx.x (xx.x)	
Pain interference		(n=xxx)<br>xx.x (xx.x)	(n=xxx)<br>xx.x (xx.x)	

Percentages are based within each column

Table 29: Secondary outcomes and tertiary variables summary statistics of all randomised participants by treatment arm

		Ketamine	Morphine	Total
Total pain relief (TOTPAR)	Mean (sd)	xx.x (xx.x)	xx.x (xx.x)	xx.x (xx.x)
	Median (IQR)	xx.x (xx.x – xx.x)	xx.x (xx.x – xx.x)	xx.x (xx.x – xx.x)
	Missing (%)	N (xx%)	N (xx%)	N (xx%)
Time to perceptible analgesia	Mean (sd)	xx.x (xx.x)	xx.x (xx.x)	xx.x (xx.x)
	Median (IQR)	xx.x (xx.x – xx.x)	xx.x (xx.x – xx.x)	xx.x (xx.x – xx.x)
	Missing (%)	N (xx%)	N (xx%)	N (xx%)
Time to meaningful analgesia	Mean (sd)	xx.x (xx.x)	xx.x (xx.x)	xx.x (xx.x)
	Median (IQR)	xx.x (xx.x – xx.x)	xx.x (xx.x – xx.x)	xx.x (xx.x – xx.x)
	Missing (%)	N (xx%)	N (xx%)	N (xx%)
Time to peak analgesia	Mean (sd)	xx.x (xx.x)	xx.x (xx.x)	xx.x (xx.x)
	Median (IQR)	xx.x (xx.x – xx.x)	xx.x (xx.x – xx.x)	xx.x (xx.x – xx.x)
	Missing (%)	N (xx%)	N (xx%)	N (xx%)
Duration of analgesia	Mean (sd)	xx.x (xx.x)	xx.x (xx.x)	xx.x (xx.x)
	Median (IQR)	xx.x (xx.x – xx.x)	xx.x (xx.x – xx.x)	xx.x (xx.x – xx.x)
	Missing (%)	N (xx%)	N (xx%)	N (xx%)
Time to first noticeable pain relief (mins)*	Mean (sd)	xx.x (xx.x)	xx.x (xx.x)	xx.x (xx.x)
	Median (IQR)	xx.x (xx.x – xx.x)	xx.x (xx.x – xx.x)	xx.x (xx.x – xx.x)
	Missing (%)	N (xx%)	N (xx%)	N (xx%)
Time to first adequate pain relief (mins)*	Mean (sd)	xx.x (xx.x)	xx.x (xx.x)	xx.x (xx.x)
	Median (IQR)	xx.x (xx.x – xx.x)	xx.x (xx.x – xx.x)	xx.x (xx.x – xx.x)
	Missing (%)	N (xx%)	N (xx%)	N (xx%)

Time taken to arrive to hospital*	Mean (sd)	xx.x (xx.x)	xx.x (xx.x)	xx.x (xx.x)
	Median (IQR)	xx.x (xx.x – xx.x)	xx.x (xx.x – xx.x)	xx.x (xx.x – xx.x)
	Missing (%)	N (xx%)	N (xx%)	N (xx%)
Patient global impression of change	Very much improved	N (xx%)	N (xx%)	N (xx%)
	Much improved	N (xx%)	N (xx%)	N (xx%)
	Minimally improved	N (xx%)	N (xx%)	N (xx%)
	No change	N (xx%)	N (xx%)	N (xx%)
	Minimally worse	N (xx%)	N (xx%)	N (xx%)
	Much worse	N (xx%)	N (xx%)	N (xx%)
	Very much worse	N (xx%)	N (xx%)	N (xx%)
	Missing	N (xx%)	N (xx%)	N (xx%)

*time taken from point of randomisation

Percentages are based within each column

Table 30: Secondary outcome study outcome at baseline

	Ketamine	Morphine	Unadjusted estimate (95% CI); p-value	Adjusted estimate (95% CI)*; p-value	Unadjusted Difference (95% CI)	Adjusted Difference (95% CI)
Total pain relief (TOTPAR), mean(sd)	(n=xxx) xx.x (xx.x)	(n=xxx) xx.x (xx.x)	MD, xx.x (xx.x to xx.x) ; 0.xx	MD, xx.x (xx.x to xx.x) ; 0.xx	N/a	N/a
Time to perceptible analgesia, median (IQR)	(n=xxx) xx.x (xx.x)	(n=xxx) xx.x (xx.x)	MDND, xx.x (xx.x to xx.x); 0.xx	MD, xx.x (xx.x to xx.x) ; 0.xx		
Time to meaningful analgesia, median (IQR)	(n=xxx) xx.x (xx.x)	(n=xxx) xx.x (xx.x)	HR, x.xx (x.xx to x.xx) ; 0.xx	HR, x.xx (x.xx to x.xx) ; 0.xx		
Time to peak analgesia, median (IQR)	(n=xxx) xx.x (xx.x)	(n=xxx) xx.x (xx.x)	HR, x.xx (x.xx to x.xx) ; 0.xx	HR, x.xx (x.xx to x.xx) ; 0.xx		
Duration of analgesia, median (IQR)	(n=xxx) xx.x (xx.x)	(n=xxx) xx.x (xx.x)	HR, x.xx (x.xx to x.xx) ; 0.xx	HR, x.xx (x.xx to x.xx) ; 0.xx		
Required rescue analgesia, N (%)	(n=xxx) xxx (xx.x%)	(n=xxx) xxx (xx.x%)	OR, x.xx (x.xx to x.xx) ; 0.xx	OR, x.xx (x.xx to x.xx) ; 0.xx	Xx (xx to xx)	Xx (xx to xx)

Final pain score below 4/10, N (%)	(n=xxx) xxx (xx.x%)	(n=xxx) xxx (xx.x%)	OR, x.xx (x.xx to x.xx) ; 0.xx	OR, x.xx (x.xx to x.xx) ; 0.xx	Xx (xx to xx)	Xx (xx to xx)
Patient global impression of change, N (%)			OR, x.xx (x.xx to x.xx) ; 0.xx	OR, x.xx (x.xx to x.xx) ; 0.xx	Xx (xx to xx)	Xx (xx to xx)
Very much improved	(n=xxx) xxx (xx.x%)	(n=xxx) xxx (xx.x%)				
Much improved	(n=xxx) xxx (xx.x%)	(n=xxx) xxx (xx.x%)				
Minimally improved	(n=xxx) xxx (xx.x%)	(n=xxx) xxx (xx.x%)				
No change	(n=xxx) xxx (xx.x%)	(n=xxx) xxx (xx.x%)				
Minimally worse	(n=xxx) xxx (xx.x%)	(n=xxx) xxx (xx.x%)				
Much worse	(n=xxx) xxx (xx.x%)	(n=xxx) xxx (xx.x%)				
Very much worse	(n=xxx) xxx (xx.x%)	(n=xxx) xxx (xx.x%)				

Percentages are based within each column

Table 31: Rescue analgesia and other treatments characteristics

		Ketamine	Morphine	Total
Required rescue analgesia	Yes	N (xx%)	N (xx%)	N (xx%)
	No	N (xx%)	N (xx%)	N (xx%)
	Missing	N (xx%)	N (xx%)	N (xx%)
Required adjunctive analgesia	Yes	N (xx%)	N (xx%)	N (xx%)
	No	N (xx%)	N (xx%)	N (xx%)
	Missing	N (xx%)	N (xx%)	N (xx%)
Type of analgesia	Entonox	N (xx%)	N (xx%)	N (xx%)
	Paracetamol	N (xx%)	N (xx%)	N (xx%)
	Ibuprofen	N (xx%)	N (xx%)	N (xx%)
	Other	N (xx%)	N (xx%)	N (xx%)
	Missing	N (xx%)	N (xx%)	N (xx%)
Midazolam administered	Yes	N (xx%)	N (xx%)	N (xx%)
	No	N (xx%)	N (xx%)	N (xx%)
	Missing	N (xx%)	N (xx%)	N (xx%)
Midazolam dose	Mean (sd)	xx.x (xx.x)	xx.x (xx.x)	xx.x (xx.x)
	Median (IQR)	xx.x (xx.x – xx.x)	xx.x (xx.x – xx.x)	xx.x (xx.x – xx.x)
	Missing	N (xx%)	N (xx%)	N (xx%)

Naloxone administered	Yes	N (xx%)	N (xx%)	N (xx%)
	No	N (xx%)	N (xx%)	N (xx%)
	Missing	N (xx%)	N (xx%)	N (xx%)
Naloxone dose	Mean (sd)	xx.x (xx.x)	xx.x (xx.x)	xx.x (xx.x)
	Median (IQR)	xx.x (xx.x – xx.x)	xx.x (xx.x – xx.x)	xx.x (xx.x – xx.x)
	Missing	N (xx%)	N (xx%)	N (xx%)
Final pain score below 4/10	Yes	N (xx%)	N (xx%)	N (xx%)
	No	N (xx%)	N (xx%)	N (xx%)
	Missing	N (xx%)	N (xx%)	N (xx%)
Participant sustained IMP overdose	Yes	N (xx%)	N (xx%)	N (xx%)
	No	N (xx%)	N (xx%)	N (xx%)
	Missing	N (xx%)	N (xx%)	N (xx%)
Participant sustained IMP underdose	Yes	N (xx%)	N (xx%)	N (xx%)
	No	N (xx%)	N (xx%)	N (xx%)
	Missing	N (xx%)	N (xx%)	N (xx%)

Percentages are based within each column

Table 32: Ambulance vital signs characteristics of all randomised participants by treatment arm

Vital Signs		Ketamine	Morphine	Total
Blood pressure Systolic (mmHg)	Mean (sd)	xx.x (xx.x)	xx.x (xx.x)	xx.x (xx.x)
	Median (IQR)	xx.x (xx.x – xx.x)	xx.x (xx.x – xx.x)	xx.x (xx.x – xx.x)
	Missing (%)	N (xx%)	N (xx%)	N (xx%)
Blood pressure Diastolic (mmHg)	Mean (sd)	xx.x (xx.x)	xx.x (xx.x)	xx.x (xx.x)
	Median (IQR)	xx.x (xx.x – xx.x)	xx.x (xx.x – xx.x)	xx.x (xx.x – xx.x)
	Missing (%)	N (xx%)	N (xx%)	N (xx%)
Pulse rate (bpm)	Mean (sd)	xx.x (xx.x)	xx.x (xx.x)	xx.x (xx.x)
	Median (IQR)	xx.x (xx.x – xx.x)	xx.x (xx.x – xx.x)	xx.x (xx.x – xx.x)
	Missing (%)	N (xx%)	N (xx%)	N (xx%)
Respiration rate (bpm)	Mean (sd)	xx.x (xx.x)	xx.x (xx.x)	xx.x (xx.x)
	Median (IQR)	xx.x (xx.x – xx.x)	xx.x (xx.x – xx.x)	xx.x (xx.x – xx.x)
	Missing (%)	N (xx%)	N (xx%)	N (xx%)
Oxygen sats (%)	Mean (sd)	xx.x (xx.x)	xx.x (xx.x)	xx.x (xx.x)
	Median (IQR)	xx.x (xx.x – xx.x)	xx.x (xx.x – xx.x)	xx.x (xx.x – xx.x)
	Missing (%)	N (xx%)	N (xx%)	N (xx%)
Glasgow coma scale (GCS) eye	1	N (xx%)	N (xx%)	N (xx%)
	2	N (xx%)	N (xx%)	N (xx%)
	3	N (xx%)	N (xx%)	N (xx%)
	4	N (xx%)	N (xx%)	N (xx%)
	Missing (%)	N (xx%)	N (xx%)	N (xx%)
Glasgow coma scale (GCS) motor	1	N (xx%)	N (xx%)	N (xx%)
	2	N (xx%)	N (xx%)	N (xx%)
	3	N (xx%)	N (xx%)	N (xx%)
	4	N (xx%)	N (xx%)	N (xx%)
	5	N (xx%)	N (xx%)	N (xx%)
	6	N (xx%)	N (xx%)	N (xx%)
	Missing (%)	N (xx%)	N (xx%)	N (xx%)
	1	N (xx%)	N (xx%)	N (xx%)

Glasgow coma scale (GCS) verbal	2	N (xx%)	N (xx%)	N (xx%)
	3	N (xx%)	N (xx%)	N (xx%)
	4	N (xx%)	N (xx%)	N (xx%)
	5	N (xx%)	N (xx%)	N (xx%)
	Missing (%)	N (xx%)	N (xx%)	N (xx%)
Glasgow coma scalw (GCS) total	3	N (xx%)	N (xx%)	N (xx%)
	4	N (xx%)	N (xx%)	N (xx%)
	5	N (xx%)	N (xx%)	N (xx%)
	6	N (xx%)	N (xx%)	N (xx%)
	7	N (xx%)	N (xx%)	N (xx%)
	8	N (xx%)	N (xx%)	N (xx%)
	9	N (xx%)	N (xx%)	N (xx%)
	10	N (xx%)	N (xx%)	N (xx%)
	11	N (xx%)	N (xx%)	N (xx%)
	12	N (xx%)	N (xx%)	N (xx%)
	13	N (xx%)	N (xx%)	N (xx%)
	14	N (xx%)	N (xx%)	N (xx%)
	15	N (xx%)	N (xx%)	N (xx%)
	Missing (%)	N (xx%)	N (xx%)	N (xx%)

Percentages are based within each column

Table 33: Ambulance vital signs study outcomes, [mean(sd)]

	Ketamine	Morphine	Unadjusted estimate (95% CI); p-value	Adjusted estimate (95% CI)*; p-value
Blood pressure Systolic (mmHg)	(n=xxx) xx.x (xx.x)	(n=xxx) xx.x (xx.x)	MD, xx.x (xx.x to xx.x)	MD, xx.x (xx.x to xx.x) ; 0.xx
Blood pressure Diastolic (mmHg)	(n=xxx) xx.x (xx.x)	(n=xxx) xx.x (xx.x)	MD, xx.x (xx.x to xx.x)	MD, xx.x (xx.x to xx.x) ; 0.xx
Pulse rate (bpm)	(n=xxx) xx.x (xx.x)	(n=xxx) xx.x (xx.x)	MD, xx.x (xx.x to xx.x)	MD, xx.x (xx.x to xx.x) ; 0.xx
Respiration rate (bpm)	(n=xxx) xx.x (xx.x)	(n=xxx) xx.x (xx.x)	MD, xx.x (xx.x to xx.x)	MD, xx.x (xx.x to xx.x) ; 0.xx
Oxygen sats (%)	(n=xxx) xx.x (xx.x)	(n=xxx) xx.x (xx.x)	MD, xx.x (xx.x to xx.x)	MD, xx.x (xx.x to xx.x) ; 0.xx
Glasgow coma scale (GCS)	(n=xxx) xx.x (xx.x)	(n=xxx) xx.x (xx.x)	OR, x.xx (x.xx to x.xx) ; 0.xx	OR, x.xx (x.xx to x.xx) ; 0.xx

Percentages are based within each column

Table 34: Expected adverse event of all randomised participants by treatment arm

	Ketamine	Morphine	Total

Experienced adverse event			N (xx%)	N (xx%)	N (xx%)
Airway	Vomiting	Yes	N (xx%)	N (xx%)	N (xx%)
		No	N (xx%)	N (xx%)	N (xx%)
		Missing	N (xx%)	N (xx%)	N (xx%)
	Aspiration	Yes	N (xx%)	N (xx%)	N (xx%)
		No	N (xx%)	N (xx%)	N (xx%)
		Missing	N (xx%)	N (xx%)	N (xx%)
	Advanced airway management	Yes	N (xx%)	N (xx%)	N (xx%)
		No	N (xx%)	N (xx%)	N (xx%)
		Missing	N (xx%)	N (xx%)	N (xx%)
Respiratory	Desaturation	Yes	N (xx%)	N (xx%)	N (xx%)
		No	N (xx%)	N (xx%)	N (xx%)
		Missing	N (xx%)	N (xx%)	N (xx%)
	Need for ventilatory support	Yes	N (xx%)	N (xx%)	N (xx%)
		No	N (xx%)	N (xx%)	N (xx%)
		Missing	N (xx%)	N (xx%)	N (xx%)
Cardiovascular	Arrhythmia	Yes	N (xx%)	N (xx%)	N (xx%)
		No	N (xx%)	N (xx%)	N (xx%)
		Missing	N (xx%)	N (xx%)	N (xx%)
	Hypotension	Yes	N (xx%)	N (xx%)	N (xx%)
		No	N (xx%)	N (xx%)	N (xx%)
		Missing	N (xx%)	N (xx%)	N (xx%)
	Hypertension	Yes	N (xx%)	N (xx%)	N (xx%)
		No	N (xx%)	N (xx%)	N (xx%)
		Missing	N (xx%)	N (xx%)	N (xx%)
Neurologic	Sedation	Yes	N (xx%)	N (xx%)	N (xx%)
		No	N (xx%)	N (xx%)	N (xx%)
		Missing	N (xx%)	N (xx%)	N (xx%)
	Excitatory movements	Yes	N (xx%)	N (xx%)	N (xx%)
		No	N (xx%)	N (xx%)	N (xx%)
		Missing	N (xx%)	N (xx%)	N (xx%)
	Adverse behavioural reactions	Yes	N (xx%)	N (xx%)	N (xx%)
		No	N (xx%)	N (xx%)	N (xx%)
		Missing	N (xx%)	N (xx%)	N (xx%)
Other	Allergic reaction	Yes	N (xx%)	N (xx%)	N (xx%)
		No	N (xx%)	N (xx%)	N (xx%)
		Missing	N (xx%)	N (xx%)	N (xx%)
	Nausea	Yes	N (xx%)	N (xx%)	N (xx%)
		No	N (xx%)	N (xx%)	N (xx%)
		Missing	N (xx%)	N (xx%)	N (xx%)

Percentages are based within each column

Table 35: Incidence of side effects and adverse events study outcomes, [N/total (%)]

	Ketamine	Morphine	Unadjusted estimate (95% CI); p-value	Adjusted estimate (95% CI)*; p-value	Unadjusted Differenc	Adjusted Difference (95% CI)

						e (95% CI)	
Experienced adverse event		(n=xxx) xxx (xx.x%)	(n=xxx) xxx (xx.x%)	OR, x.xx (x.xx to x.xx) ; 0.xx	OR, x.xx (x.xx to x.xx) ; 0.xx	Xx (xx to xx)	Xx (xx to xx)
Airway	Vomiting	(n=xxx) xxx (xx.x%)	(n=xxx) xxx (xx.x%)	OR, x.xx (x.xx to x.xx) ; 0.xx	OR, x.xx (x.xx to x.xx) ; 0.xx	Xx (xx to xx)	Xx (xx to xx)
	Aspiration	(n=xxx) xxx (xx.x%)	(n=xxx) xxx (xx.x%)	OR, x.xx (x.xx to x.xx) ; 0.xx	OR, x.xx (x.xx to x.xx) ; 0.xx	Xx (xx to xx)	Xx (xx to xx)
	Advanced airway managemen t	(n=xxx) xxx (xx.x%)	(n=xxx) xxx (xx.x%)	OR, x.xx (x.xx to x.xx) ; 0.xx	OR, x.xx (x.xx to x.xx) ; 0.xx	Xx (xx to xx)	Xx (xx to xx)
Respirato ry	Desaturation	(n=xxx) xxx (xx.x%)	(n=xxx) xxx (xx.x%)	OR, x.xx (x.xx to x.xx) ; 0.xx	OR, x.xx (x.xx to x.xx) ; 0.xx	Xx (xx to xx)	Xx (xx to xx)
	Need for ventilatory support	(n=xxx) xxx (xx.x%)	(n=xxx) xxx (xx.x%)	OR, x.xx (x.xx to x.xx) ; 0.xx	OR, x.xx (x.xx to x.xx) ; 0.xx	Xx (xx to xx)	Xx (xx to xx)
Cardiova scular	Arrhythmia	(n=xxx) xxx (xx.x%)	(n=xxx) xxx (xx.x%)	OR, x.xx (x.xx to x.xx) ; 0.xx	OR, x.xx (x.xx to x.xx) ; 0.xx	Xx (xx to xx)	Xx (xx to xx)
	Hypotension	(n=xxx) xxx (xx.x%)	(n=xxx) xxx (xx.x%)	OR, x.xx (x.xx to x.xx) ; 0.xx	OR, x.xx (x.xx to x.xx) ; 0.xx	Xx (xx to xx)	Xx (xx to xx)
	Hypertensio n	(n=xxx) xxx (xx.x%)	(n=xxx) xxx (xx.x%)	OR, x.xx (x.xx to x.xx) ; 0.xx	OR, x.xx (x.xx to x.xx) ; 0.xx	Xx (xx to xx)	Xx (xx to xx)
Neurolog ic	Sedation	(n=xxx) xxx (xx.x%)	(n=xxx) xxx (xx.x%)	OR, x.xx (x.xx to x.xx) ; 0.xx	OR, x.xx (x.xx to x.xx) ; 0.xx	Xx (xx to xx)	Xx (xx to xx)
	Excitatory movements	(n=xxx) xxx (xx.x%)	(n=xxx) xxx (xx.x%)	OR, x.xx (x.xx to x.xx) ; 0.xx	OR, x.xx (x.xx to x.xx) ; 0.xx	Xx (xx to xx)	Xx (xx to xx)
	Adverse behavioural reactions	(n=xxx) xxx (xx.x%)	(n=xxx) xxx (xx.x%)	OR, x.xx (x.xx to x.xx) ; 0.xx	OR, x.xx (x.xx to x.xx) ; 0.xx	Xx (xx to xx)	Xx (xx to xx)
Other	Allergic reaction	(n=xxx) xxx (xx.x%)	(n=xxx) xxx (xx.x%)	OR, x.xx (x.xx to x.xx) ; 0.xx	OR, x.xx (x.xx to x.xx) ; 0.xx	Xx (xx to xx)	Xx (xx to xx)
	Nausea	(n=xxx) xxx (xx.x%)	(n=xxx) xxx (xx.x%)	OR, x.xx (x.xx to x.xx) ; 0.xx	OR, x.xx (x.xx to x.xx) ; 0.xx	Xx (xx to xx)	Xx (xx to xx)

Percentages are based within each column

Table 36: Ambulance resource use for all randomised participants by treatment arm

		Ketamine	Morphine	Total
Ambulance job cycle time	Mean (sd)	xx.x (xx.x)	xx.x (xx.x)	xx.x (xx.x)
	Median (IQR)	xx.x (xx.x – xx.x)	xx.x (xx.x – xx.x)	xx.x (xx.x – xx.x)
	Missing	N (xx%)	N (xx%)	N (xx%)
Cumulative IMP dose	Mean (sd)	xx.x (xx.x)	xx.x (xx.x)	xx.x (xx.x)
	Median (IQR)	xx.x (xx.x – xx.x)	xx.x (xx.x – xx.x)	xx.x (xx.x – xx.x)
	Missing	N (xx%)	N (xx%)	N (xx%)
	Total	xx.x (xx.x)	xx.x (xx.x)	xx.x (xx.x)

Number of paramedics	Mean (sd)	xx.x (xx.x)	xx.x (xx.x)	xx.x (xx.x)
	Median (IQR)	xx.x (xx.x – xx.x)	xx.x (xx.x – xx.x)	xx.x (xx.x – xx.x)
	Missing	N (xx%)	N (xx%)	N (xx%)
Number of ambulance clinicians	Total	xx.x (xx.x)	xx.x (xx.x)	xx.x (xx.x)
	Mean (sd)	xx.x (xx.x)	xx.x (xx.x)	xx.x (xx.x)
	Median (IQR)	xx.x (xx.x – xx.x)	xx.x (xx.x – xx.x)	xx.x (xx.x – xx.x)
	Missing	N (xx%)	N (xx%)	N (xx%)
Number of ambulance technicians/students	Total	xx.x (xx.x)	xx.x (xx.x)	xx.x (xx.x)
	Mean (sd)	xx.x (xx.x)	xx.x (xx.x)	xx.x (xx.x)
	Median (IQR)	xx.x (xx.x – xx.x)	xx.x (xx.x – xx.x)	xx.x (xx.x – xx.x)
	Missing	N (xx%)	N (xx%)	N (xx%)
Number of doctors	Total	xx.x (xx.x)	xx.x (xx.x)	xx.x (xx.x)
	Mean (sd)	xx.x (xx.x)	xx.x (xx.x)	xx.x (xx.x)
	Median (IQR)	xx.x (xx.x – xx.x)	xx.x (xx.x – xx.x)	xx.x (xx.x – xx.x)
	Missing	N (xx%)	N (xx%)	N (xx%)
Number of others attending	Total	xx.x (xx.x)	xx.x (xx.x)	xx.x (xx.x)
	Mean (sd)	xx.x (xx.x)	xx.x (xx.x)	xx.x (xx.x)
	Median (IQR)	xx.x (xx.x – xx.x)	xx.x (xx.x – xx.x)	xx.x (xx.x – xx.x)
	Missing	N (xx%)	N (xx%)	N (xx%)

Percentages are based within each column

Table 37: Resource use during ambulance journey study outcomes

	Ketamine	Morphine	Unadjusted estimate (95% CI); p-value	Adjusted estimate (95% CI)*; p-value
Ambulance job cycle time, median (IQR)	(n=xxx) xx.x (xx.x)	(n=xxx) xx.x (xx.x)	HR, x.xx (x.xx to x.xx) ; 0.xx	HR, x.xx (x.xx to x.xx) ; 0.xx
Cumulative IMP dose, mean(sd)	(n=xxx) xx.x (xx.x)	(n=xxx) xx.x (xx.x)	MD, xx.x (xx.x to xx.x); 0.xx	MD, xx.x (xx.x to xx.x) ; 0.xx
Number of paramedics, mean (sd)	(n=xxx) xx.x (xx.x)	(n=xxx) xx.x (xx.x)	MD, xx.x (xx.x to xx.x); 0.xx	MD, xx.x (xx.x to xx.x) ; 0.xx
Number of ambulance clinicians, mean (sd)	(n=xxx) xx.x (xx.x)	(n=xxx) xx.x (xx.x)	MD, xx.x (xx.x to xx.x); 0.xx	MD, xx.x (xx.x to xx.x) ; 0.xx
Number of ambulance technicians/students, mean (sd)	(n=xxx) xx.x (xx.x)	(n=xxx) xx.x (xx.x)	MD, xx.x (xx.x to xx.x); 0.xx	MD, xx.x (xx.x to xx.x) ; 0.xx
Number of doctors , mean (sd)	(n=xxx) xx.x (xx.x)	(n=xxx) xx.x (xx.x)	MD, xx.x (xx.x to xx.x); 0.xx	MD, xx.x (xx.x to xx.x) ; 0.xx

Number of others attending, mean (sd)	(n=xxx)<br>xx.x (xx.x)	(n=xxx)<br>xx.x (xx.x)	MD, xx.x (xx.x to xx.x); 0.xx	MD, xx.x (xx.x to xx.x) ; 0.xx

Percentages are based within each column

Table 38: Hospital stay and procedures for all randomised participants by treatment arm

		Ketamine	Morphine	Total
Length of stay in ED	Mean (sd)	xx.x (xx.x)	xx.x (xx.x)	xx.x (xx.x)
	Median (IQR)	xx.x (xx.x – xx.x)	xx.x (xx.x – xx.x)	xx.x (xx.x – xx.x)
	Missing	N (xx%)	N (xx%)	N (xx%)
Patient admitted to hospital	Yes	N (xx%)	N (xx%)	N (xx%)
	No	N (xx%)	N (xx%)	N (xx%)
	Missing	N (xx%)	N (xx%)	N (xx%)
Admitted to critical care	Yes	N (xx%)	N (xx%)	N (xx%)
	No	N (xx%)	N (xx%)	N (xx%)
	Missing	N (xx%)	N (xx%)	N (xx%)
Days in receiving level 1/2 critical care	Mean (sd)	xx.x (xx.x)	xx.x (xx.x)	xx.x (xx.x)
	Median (IQR)	xx.x (xx.x – xx.x)	xx.x (xx.x – xx.x)	xx.x (xx.x – xx.x)
	Missing	N (xx%)	N (xx%)	N (xx%)
Days in receiving level 3 critical care	Mean (sd)	xx.x (xx.x)	xx.x (xx.x)	xx.x (xx.x)
	Median (IQR)	xx.x (xx.x – xx.x)	xx.x (xx.x – xx.x)	xx.x (xx.x – xx.x)
	Missing	N (xx%)	N (xx%)	N (xx%)
Days in receiving unknown level of critical care	Mean (sd)	xx.x (xx.x)	xx.x (xx.x)	xx.x (xx.x)
	Median (IQR)	xx.x (xx.x – xx.x)	xx.x (xx.x – xx.x)	xx.x (xx.x – xx.x)
	Missing	N (xx%)	N (xx%)	N (xx%)
Discharge location	Normal residence	N (xx%)	N (xx%)	N (xx%)
	Rehabilitation service	N (xx%)	N (xx%)	N (xx%)
	Another acute hospital	N (xx%)	N (xx%)	N (xx%)
	Death in hospital	N (xx%)	N (xx%)	N (xx%)
	Missing	N (xx%)	N (xx%)	N (xx%)
Participant entered to TARN	Yes	N (xx%)	N (xx%)	N (xx%)
	No	N (xx%)	N (xx%)	N (xx%)
	Missing	N (xx%)	N (xx%)	N (xx%)
CT Scan	Yes	N (xx%)	N (xx%)	N (xx%)
	No	N (xx%)	N (xx%)	N (xx%)
	Missing	N (xx%)	N (xx%)	N (xx%)
Number of CT scans per patient	Mean (sd)	xx.x (xx.x)	xx.x (xx.x)	xx.x (xx.x)
	Median (IQR)	xx.x (xx.x – xx.x)	xx.x (xx.x – xx.x)	xx.x (xx.x – xx.x)
	Missing	N (xx%)	N (xx%)	N (xx%)

Percentages are based within each column

Table 39: Resource use at hospital study outcomes

	Ketamine	Morphine	Unadjusted estimate (95% CI); p-value	Adjusted estimate (95% CI)*; p-value	Unadjusted Difference (95% CI)	Adjusted Difference (95% CI)

Length of stay in ED, median (IQR)	(n=xxx) xx.x (xx.x)	(n=xxx) xx.x (xx.x)	HR, x.xx (x.xx to x.xx) ; 0.xx	HR, x.xx (x.xx to x.xx) ; 0.xx		
Patient admitted to hospital, N (%)	(n=xxx) xxx (xx.x%)	(n=xxx) xxx (xx.x%)	OR, x.xx (x.xx to x.xx) ; 0.xx	OR, x.xx (x.xx to x.xx) ; 0.xx	Xx (xx to xx)	Xx (xx to xx)
Admitted to critical care, N (%)	(n=xxx) xxx (xx.x%)	(n=xxx) xxx (xx.x%)	OR, x.xx (x.xx to x.xx) ; 0.xx	OR, x.xx (x.xx to x.xx) ; 0.xx	Xx (xx to xx)	Xx (xx to xx)
Days in receiving level 1/2 critical care, mean (sd)	(n=xxx) xx.x (xx.x)	(n=xxx) xx.x (xx.x)	MD, xx.x (xx.x to xx.x); 0.xx	MD, xx.x (xx.x to xx.x) ; 0.xx		
Days in receiving level 3 critical care, mean (sd)	(n=xxx) xx.x (xx.x)	(n=xxx) xx.x (xx.x)	MD, xx.x (xx.x to xx.x); 0.xx	MD, xx.x (xx.x to xx.x) ; 0.xx		
Days in receiving unknown level of critical care, mean (sd)	(n=xxx) xx.x (xx.x)	(n=xxx) xx.x (xx.x)	MD, xx.x (xx.x to xx.x); 0.xx	MD, xx.x (xx.x to xx.x) ; 0.xx		
Length of stay hospital, mean (sd)	(n=xxx) xx.x (xx.x)	(n=xxx) xx.x (xx.x)	MD, xx.x (xx.x to xx.x); 0.xx	MD, xx.x (xx.x to xx.x) ; 0.xx		
Participant entered to TARN, N (%)	(n=xxx) xxx (xx.x%)	(n=xxx) xxx (xx.x%)	OR, x.xx (x.xx to x.xx) ; 0.xx	OR, x.xx (x.xx to x.xx) ; 0.xx	Xx (xx to xx)	Xx (xx to xx)
CT Scan use, N (%)	(n=xxx) xxx (xx.x%)	(n=xxx) xxx (xx.x%)	OR, x.xx (x.xx to x.xx) ; 0.xx	OR, x.xx (x.xx to x.xx) ; 0.xx	Xx (xx to xx)	Xx (xx to xx)
Number of CT scans per patient, mean (sd)	(n=xxx) xx.x (xx.x)	(n=xxx) xx.x (xx.x)	MD, xx.x (xx.x to xx.x); 0.xx	MD, xx.x (xx.x to xx.x) ; 0.xx		

Percentages are based within each column

Table 40: Longer term outcomes for all randomised participants by treatment arm at 3 month follow up

	Ketamine	Morphine	Total

Pain severity	Overall	Mean (sd)	xx.x (xx.x)	xx.x (xx.x)	xx.x (xx.x)
		Median (IQR)	xx.x (xx.x – xx.x)	xx.x (xx.x – xx.x)	xx.x (xx.x – xx.x)
		Missing	N (xx%)	N (xx%)	N (xx%)
	Worst pain	Mean (sd)	xx.x (xx.x)	xx.x (xx.x)	xx.x (xx.x)
		Median (IQR)	xx.x (xx.x – xx.x)	xx.x (xx.x – xx.x)	xx.x (xx.x – xx.x)
		Missing	N (xx%)	N (xx%)	N (xx%)
	Least pain	Mean (sd)	xx.x (xx.x)	xx.x (xx.x)	xx.x (xx.x)
		Median (IQR)	xx.x (xx.x – xx.x)	xx.x (xx.x – xx.x)	xx.x (xx.x – xx.x)
		Missing	N (xx%)	N (xx%)	N (xx%)
	Average pain	Mean (sd)	xx.x (xx.x)	xx.x (xx.x)	xx.x (xx.x)
		Median (IQR)	xx.x (xx.x – xx.x)	xx.x (xx.x – xx.x)	xx.x (xx.x – xx.x)
		Missing	N (xx%)	N (xx%)	N (xx%)
	Pain now	Mean (sd)	xx.x (xx.x)	xx.x (xx.x)	xx.x (xx.x)
		Median (IQR)	xx.x (xx.x – xx.x)	xx.x (xx.x – xx.x)	xx.x (xx.x – xx.x)
		Missing	N (xx%)	N (xx%)	N (xx%)
Pain interference		Mean (sd)	xx.x (xx.x)	xx.x (xx.x)	xx.x (xx.x)
		Median (IQR)	xx.x (xx.x – xx.x)	xx.x (xx.x – xx.x)	xx.x (xx.x – xx.x)
		Missing	N (xx%)	N (xx%)	N (xx%)

Percentages are based within each column

Table 41: Study outcomes at 3 month follow up, [mean(sd)]

		Ketamine	Morphine	Unadjusted estimate (95% CI); p-value	Adjusted estimate (95% CI)*; p-value
Pain severity	Overall	(n=xxx) xx.x (xx.x)	(n=xxx) xx.x (xx.x)	MD, xx.x (xx.x to xx.x); 0.xx	MD, xx.x (xx.x to xx.x) ; 0.xx
	Worst pain	(n=xxx) xx.x (xx.x)	(n=xxx) xx.x (xx.x)	MD, xx.x (xx.x to xx.x); 0.xx	MD, xx.x (xx.x to xx.x) ; 0.xx
	Least pain	(n=xxx) xx.x (xx.x)	(n=xxx) xx.x (xx.x)	MD, xx.x (xx.x to xx.x); 0.xx	MD, xx.x (xx.x to xx.x) ; 0.xx
	Average pain	(n=xxx) xx.x (xx.x)	(n=xxx) xx.x (xx.x)	MD, xx.x (xx.x to xx.x); 0.xx	MD, xx.x (xx.x to xx.x) ; 0.xx
	Pain now	(n=xxx) xx.x (xx.x)	(n=xxx) xx.x (xx.x)	MD, xx.x (xx.x to xx.x); 0.xx	MD, xx.x (xx.x to xx.x) ; 0.xx
Pain interference		(n=xxx) xx.x (xx.x)	(n=xxx) xx.x (xx.x)	MD, xx.x (xx.x to xx.x); 0.xx	MD, xx.x (xx.x to xx.x); 0.xx

Percentages are based within each column

Table 42: Longer term outcomes for all randomised participants by treatment arm at 6 month follow up

	Ketamine	Morphine	Total

Pain severity	Overall	Mean (sd)	xx.x (xx.x)	xx.x (xx.x)	xx.x (xx.x)
		Median (IQR)	xx.x (xx.x – xx.x)	xx.x (xx.x – xx.x)	xx.x (xx.x – xx.x)
		Missing	N (xx%)	N (xx%)	N (xx%)
	Worst pain	Mean (sd)	xx.x (xx.x)	xx.x (xx.x)	xx.x (xx.x)
		Median (IQR)	xx.x (xx.x – xx.x)	xx.x (xx.x – xx.x)	xx.x (xx.x – xx.x)
		Missing	N (xx%)	N (xx%)	N (xx%)
	Least pain	Mean (sd)	xx.x (xx.x)	xx.x (xx.x)	xx.x (xx.x)
		Median (IQR)	xx.x (xx.x – xx.x)	xx.x (xx.x – xx.x)	xx.x (xx.x – xx.x)
		Missing	N (xx%)	N (xx%)	N (xx%)
	Average pain	Mean (sd)	xx.x (xx.x)	xx.x (xx.x)	xx.x (xx.x)
		Median (IQR)	xx.x (xx.x – xx.x)	xx.x (xx.x – xx.x)	xx.x (xx.x – xx.x)
		Missing	N (xx%)	N (xx%)	N (xx%)
	Pain now	Mean (sd)	xx.x (xx.x)	xx.x (xx.x)	xx.x (xx.x)
		Median (IQR)	xx.x (xx.x – xx.x)	xx.x (xx.x – xx.x)	xx.x (xx.x – xx.x)
		Missing	N (xx%)	N (xx%)	N (xx%)
Pain interference		Mean (sd)	xx.x (xx.x)	xx.x (xx.x)	xx.x (xx.x)
		Median (IQR)	xx.x (xx.x – xx.x)	xx.x (xx.x – xx.x)	xx.x (xx.x – xx.x)
		Missing	N (xx%)	N (xx%)	N (xx%)

Percentages are based within each column

Table 43: Study outcomes at 6 month follow up, [mean(sd)]

		Ketamine	Morphine	Unadjusted estimate (95% CI); p-value	Adjusted estimate (95% CI)*; p-value
Pain severity	Overall	(n=xxx)<br>xx.x (xx.x)	(n=xxx)<br>xx.x (xx.x)	MD, xx.x (xx.x to xx.x); 0.xx	MD, xx.x (xx.x to xx.x) ; 0.xx
	Worst pain	(n=xxx)<br>xx.x (xx.x)	(n=xxx)<br>xx.x (xx.x)	MD, xx.x (xx.x to xx.x); 0.xx	MD, xx.x (xx.x to xx.x) ; 0.xx
	Least pain	(n=xxx)<br>xx.x (xx.x)	(n=xxx)<br>xx.x (xx.x)	MD, xx.x (xx.x to xx.x); 0.xx	MD, xx.x (xx.x to xx.x) ; 0.xx
	Average pain	(n=xxx)<br>xx.x (xx.x)	(n=xxx)<br>xx.x (xx.x)	MD, xx.x (xx.x to xx.x); 0.xx	MD, xx.x (xx.x to xx.x) ; 0.xx
	Pain now	(n=xxx)<br>xx.x (xx.x)	(n=xxx)<br>xx.x (xx.x)	MD, xx.x (xx.x to xx.x); 0.xx	MD, xx.x (xx.x to xx.x) ; 0.xx
Pain interference		(n=xxx)<br>xx.x (xx.x)	(n=xxx)<br>xx.x (xx.x)	MD, xx.x (xx.x to xx.x); 0.xx	MD, xx.x (xx.x to xx.x) ; 0.xx

Percentages are based within each column

Table 44: Baseline demographic characteristics of all randomised participants by treatment arm

Baseline demographics		Ketamine	Morphine	Total
Age	Mean (sd)	xx.x (xx.x)	xx.x (xx.x)	xx.x (xx.x)
	Median (IQR)	xx.x (xx.x – xx.x)	xx.x (xx.x – xx.x)	xx.x (xx.x – xx.x)

	Missing (%)	N (xx%)	N (xx%)	N (xx%)
Gender	Male	N (xx%)	N (xx%)	N (xx%)
	Female	N (xx%)	N (xx%)	N (xx%)
	Transgender	N (xx%)	N (xx%)	N (xx%)
	Other	N (xx%)	N (xx%)	N (xx%)
	Not disclosed	N (xx%)	N (xx%)	N (xx%)
	Missing	N (xx%)	N (xx%)	N (xx%)
Ethnicity	White	N (xx%)	N (xx%)	N (xx%)
	Black	N (xx%)	N (xx%)	N (xx%)
	Mixed	N (xx%)	N (xx%)	N (xx%)
	Any other ethnic group	N (xx%)	N (xx%)	N (xx%)
	Asian	N (xx%)	N (xx%)	N (xx%)
	Ethnicity not given	N (xx%)	N (xx%)	N (xx%)
	Missing	N (xx%)	N (xx%)	N (xx%)
Weight	Mean (sd)	xx.x (xx.x)	xx.x (xx.x)	xx.x (xx.x)
	Median (IQR)	xx.x (xx.x – xx.x)	xx.x (xx.x – xx.x)	xx.x (xx.x – xx.x)
	Missing (%)	N (xx%)	N (xx%)	N (xx%)

Percentages are based within each column

Table 45: Injury characteristics of all randomised participants by treatment arm

			Ketamine	Morphine	Total
Mechanism of Injury:	Blunt Trauma		N (xx%)	N (xx%)	N (xx%)
	Penetrating Trauma		N (xx%)	N (xx%)	N (xx%)
	Burn		N (xx%)	N (xx%)	N (xx%)
	Missing		N (xx%)	N (xx%)	N (xx%)
Injuries sustained:	Fracture/ dislocation	Yes	N (xx%)	N (xx%)	N (xx%)
		No	N (xx%)	N (xx%)	N (xx%)
		Missing	N (xx%)	N (xx%)	N (xx%)
	Soft tissue injury	Yes	N (xx%)	N (xx%)	N (xx%)
		No	N (xx%)	N (xx%)	N (xx%)
		Missing	N (xx%)	N (xx%)	N (xx%)
	Wound/ laceration	Yes	N (xx%)	N (xx%)	N (xx%)
		No	N (xx%)	N (xx%)	N (xx%)
		Missing	N (xx%)	N (xx%)	N (xx%)
Body part/region injured:	Head	Yes	N (xx%)	N (xx%)	N (xx%)
		No	N (xx%)	N (xx%)	N (xx%)
		Missing	N (xx%)	N (xx%)	N (xx%)
	Neck	Yes	N (xx%)	N (xx%)	N (xx%)
		No	N (xx%)	N (xx%)	N (xx%)
		Missing	N (xx%)	N (xx%)	N (xx%)
	Chest & back	Yes	N (xx%)	N (xx%)	N (xx%)
		No	N (xx%)	N (xx%)	N (xx%)
		Missing	N (xx%)	N (xx%)	N (xx%)
	Abdomen	Yes	N (xx%)	N (xx%)	N (xx%)
		No	N (xx%)	N (xx%)	N (xx%)
		Missing	N (xx%)	N (xx%)	N (xx%)
	Pelvis	Yes	N (xx%)	N (xx%)	N (xx%)

		No	N (xx%)	N (xx%)	N (xx%)
		Missing	N (xx%)	N (xx%)	N (xx%)
	Upper limbs	Yes	N (xx%)	N (xx%)	N (xx%)
		No	N (xx%)	N (xx%)	N (xx%)
		Missing	N (xx%)	N (xx%)	N (xx%)
	Lower limbs	Yes	N (xx%)	N (xx%)	N (xx%)
		No	N (xx%)	N (xx%)	N (xx%)
		Missing	N (xx%)	N (xx%)	N (xx%)

Percentages are based within each column

Table 46: Initial vital signs of all randomised participants by treatment arm

		Ketamine	Morphine
Blood pressure Systolic (mmHg)	Mean (sd)	xx.x (xx.x)	xx.x (xx.x)
	Median (IQR)	xx.x (xx.x – xx.x)	xx.x (xx.x – xx.x)
	Missing (%)	N (xx%)	N (xx%)
Blood pressure Diastolic (mmHg)	Mean (sd)	xx.x (xx.x)	xx.x (xx.x)
	Median (IQR)	xx.x (xx.x – xx.x)	xx.x (xx.x – xx.x)
	Missing (%)	N (xx%)	N (xx%)
Heart rate (bpm)	Mean (sd)	xx.x (xx.x)	xx.x (xx.x)
	Median (IQR)	xx.x (xx.x – xx.x)	xx.x (xx.x – xx.x)
	Missing (%)	N (xx%)	N (xx%)
Respiration rate (bpm)	Mean (sd)	xx.x (xx.x)	xx.x (xx.x)
	Median (IQR)	xx.x (xx.x – xx.x)	xx.x (xx.x – xx.x)
	Missing (%)	N (xx%)	N (xx%)
Oxygen sats (%)	Mean (sd)	xx.x (xx.x)	xx.x (xx.x)
	Median (IQR)	xx.x (xx.x – xx.x)	xx.x (xx.x – xx.x)
	Missing (%)	N (xx%)	N (xx%)
Glasgow coma scale (GCS) eye	1	N (xx%)	N (xx%)
	2	N (xx%)	N (xx%)
	3	N (xx%)	N (xx%)
	4	N (xx%)	N (xx%)
	Missing (%)	N (xx%)	N (xx%)
Glasgow coma scale (GCS) motor	1	N (xx%)	N (xx%)
	2	N (xx%)	N (xx%)
	3	N (xx%)	N (xx%)
	4	N (xx%)	N (xx%)
	5	N (xx%)	N (xx%)
	6	N (xx%)	N (xx%)
	Missing (%)	N (xx%)	N (xx%)
Glasgow coma scale (GCS) verbal	1	N (xx%)	N (xx%)
	2	N (xx%)	N (xx%)
	3	N (xx%)	N (xx%)
	4	N (xx%)	N (xx%)
	5	N (xx%)	N (xx%)
	Missing (%)	N (xx%)	N (xx%)
	3	N (xx%)	N (xx%)

Glasgow coma scalw (GCS) total	4	N (xx%)	N (xx%)
	5	N (xx%)	N (xx%)
	6	N (xx%)	N (xx%)
	7	N (xx%)	N (xx%)
	8	N (xx%)	N (xx%)
	9	N (xx%)	N (xx%)
	10	N (xx%)	N (xx%)
	11	N (xx%)	N (xx%)
	12	N (xx%)	N (xx%)
	13	N (xx%)	N (xx%)
	14	N (xx%)	N (xx%)
	15	N (xx%)	N (xx%)
	Missing (%)	N (xx%)	N (xx%)

Table 47: Analysis model estimates of treatment difference of primary outcome

	**Ketamine (N=xxx)**	**Morphine (N=xxx)**	Unadjusted estimate (95% CI); p-value	Adjusted estimate (95% CI)*; p-value
**Sum of pain intensity difference, mean (sd)**				
**ITT model**	xx (xx)	xx (xx)	MD, x.xx (x.xx to x.xx); 0.xxx	MD, x.xx (x.xx to x.xx); 0.xx
**CACE model**	xx (xx)	xx (xx)	MD, x.xx (x.xx to x.xx); 0.xxx	MD, x.xx (x.xx to x.xx); 0.xx
**Pocock's Win Ratio using composite of SPID and death**	xx (xx)	xx (xx)	MD, x.xx (x.xx to x.xx); 0.xxx	MD, x.xx (x.xx to x.xx); 0.xx

*Adjusted for ambulance service, age, gender, alternative parent analgesia prior to randomisation

Table 48: Sub-group analyses of the Sum of pain intensity difference outcome

**Subgroups**	**Ketamine**<br>**N; mean(sd)**	**Morphine**<br>**N; mean(sd)**	**Effect estimate (95% CI)**	**Interaction effect (95% CI); p-value**
**Age**				
1				
2				
**Gender**				
1				
2				

**Alternative parenteral analgesia**				
1				
2				

Table 49: Completeness of health economic data by follow-up visit for all randomised participants by treatment arm

	Ketamine		Morphine		Total	
	**n**	**(%, N)**	**n**	**(%, N)**	**n**	**(%, N)**
**Health status[1]**						
EQ-5D Baseline (derived)	xxx	(xx.x%, xxx)	xxx	(xx.x%, xxx)	xxx	(xx.x%, xxx)
EQ-5D 3 months	xxx	(xx.x%, xxx)	xxx	(xx.x%, xxx)	xxx	(xx.x%, xxx)
EQ-5D 6 months	xxx	(xx.x%, xxx)	xxx	(xx.x%, xxx)	xxx	(xx.x%, xxx)
EQ-5D All visits	xxx	(xx.x%, xxx)	xxx	(xx.x%, xxx)	xxx	(xx.x%, xxx)
**Resource use[2]**						
Inpatient	xxx	(xx.x%, xxx)	xxx	(xx.x%, xxx)	xxx	(xx.x%, xxx)
Outpatient	xxx	(xx.x%, xxx)	xxx	(xx.x%, xxx)	xxx	(xx.x%, xxx)
Community	xxx	(xx.x%, xxx)	xxx	(xx.x%, xxx)	xxx	(xx.x%, xxx)
Personal social services	xxx	(xx.x%, xxx)	xxx	(xx.x%, xxx)	xxx	(xx.x%, xxx)
Wider costs	xxx	(xx.x%, xxx)	xxx	(xx.x%, xxx)	xxx	(xx.x%, xxx)

1.EQ-5D-5L index score
2. Range shown (3M,6M)

Table 50: Health status, resource use and cost (complete cases) for all randomised participants by treatment arm

	Ketamine		Morphine		Total	
	**mean**	**(SD)**	**mean**	**(SD)**	**mean**	**(SD)**
**Health status[1]**						
EQ-5D Baseline	xx.x	xx.x	xx.x	xx.x	xx.x	xx.x
EQ-5D 3 months	xx.x	xx.x	xx.x	xx.x	xx.x	xx.x
EQ-5D 6 months	xx.x	xx.x	xx.x	xx.x	xx.x	xx.x

QALYs	xx.x	xx.x	xx.x	xx.x	xx.x	xx.x
**Resource use** (all visits)						
Inpatient nights	xx.x	xx.x	xx.x	xx.x	xx.x	xx.x
Outpatient visits	xx.x	xx.x	xx.x	xx.x	xx.x	xx.x
A&E Visits	xx.x	xx.x	xx.x	xx.x	xx.x	xx.x
Community	xx.x	xx.x	xx.x	xx.x	xx.x	xx.x
GP surgery visits	xx.x	xx.x	xx.x	xx.x	xx.x	xx.x
GP home visits	xx.x	xx.x	xx.x	xx.x	xx.x	xx.x
GP telephone contacts	xx.x	xx.x	xx.x	xx.x	xx.x	xx.x
GP video/online contacts	xx.x	xx.x	xx.x	xx.x	xx.x	xx.x
District nurse contacts	xx.x	xx.x	xx.x	xx.x	xx.x	xx.x
Social worker contacts	xx.x	xx.x	xx.x	xx.x	xx.x	xx.x
Physiotherapy contacts	xx.x	xx.x	xx.x	xx.x	xx.x	xx.x
Occupational therapy contacts	xx.x	xx.x	xx.x	xx.x	xx.x	xx.x
Counsellor	xx.x	xx.x	xx.x	xx.x	xx.x	xx.x
Psychologist	xx.x	xx.x	xx.x	xx.x	xx.x	xx.x
Home help/carer	xx.x	xx.x	xx.x	xx.x	xx.x	xx.x
Other community contacts	xx.x	xx.x	xx.x	xx.x	xx.x	xx.x
Medication<br>Special Equipment<br>Wider costs	xx.x	xx.x	xx.x	xx.x	xx.x	xx.x
**Cost**[2]						
A: Cost (study procedures)	xx.x	xx.x	xx.x	xx.x	xx.x	xx.x
B: Cost (NHS contacts)	xx.x	xx.x	xx.x	xx.x	xx.x	xx.x
C: Cost (Personal social services)	xx.x	xx.x	xx.x	xx.x	xx.x	xx.x
Cost (Total, A+B+C)	xx.x	xx.x	xx.x	xx.x	xx.x	xx.x

1 EQ-5D-5L index score

2 Wider costs are not included in the analytic perspective, which includes only health service and personal social services costs

Table 51: Cost-effectiveness results

		**Incremental cost (95%CI)**	**Incremental QALYs (95%CI)**	**ICER (95%CI)**	**p[1]**	**p[2]**	**NMB[1]**	**NMB[2]**
Base case								
	Imputed costs and QALYs, baseline EQ-5D adjusted							
Sensitivity analyses								
1	Inclusion of societal costs							

2	Complete case analysis							
3	Base case: sub-group analyses specified in the SAP							
4	Baseline utility assumptions changes							
5	Cost per SPID point reduction							
[1] probability cost-effective or net monetary benefit if willing to pay £20,000/QALY. [2] probability cost-effective or net monetary benefit if willing to pay £30,000/QALY								

Figures

Figure 3: Total participants recruited by paramedic experience level by ambulance service

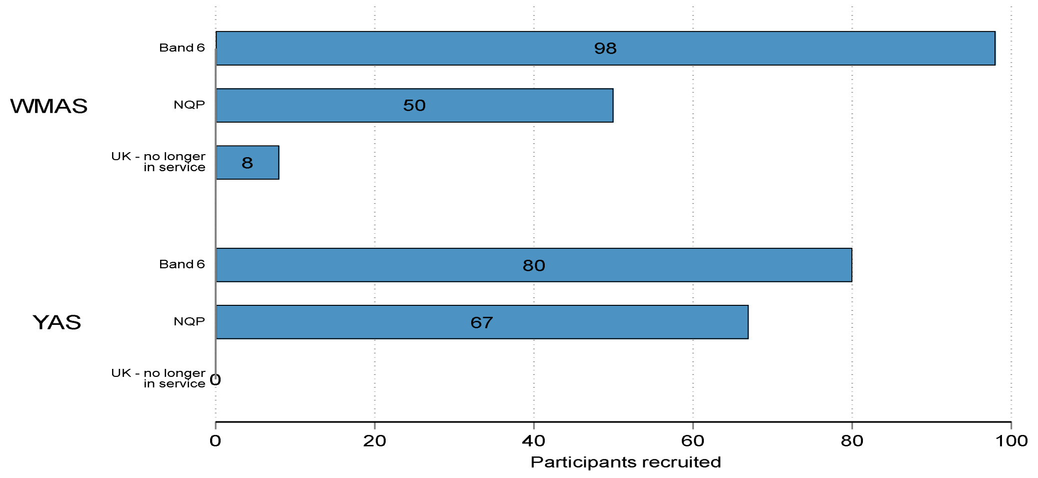

Figure 4: Trial trained paramedics by experience level by ambulance service

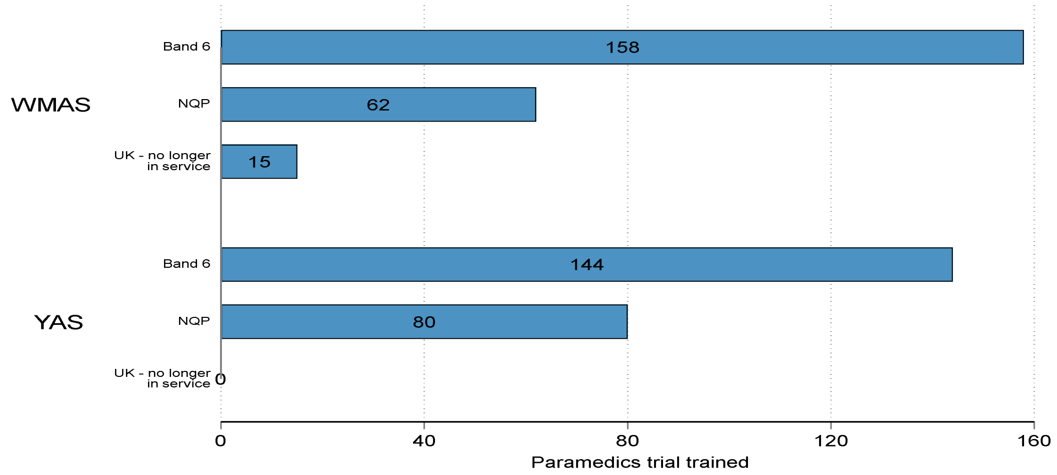

Figure 5: Percentage of participants recruited by paramedic experience for each ambulance service

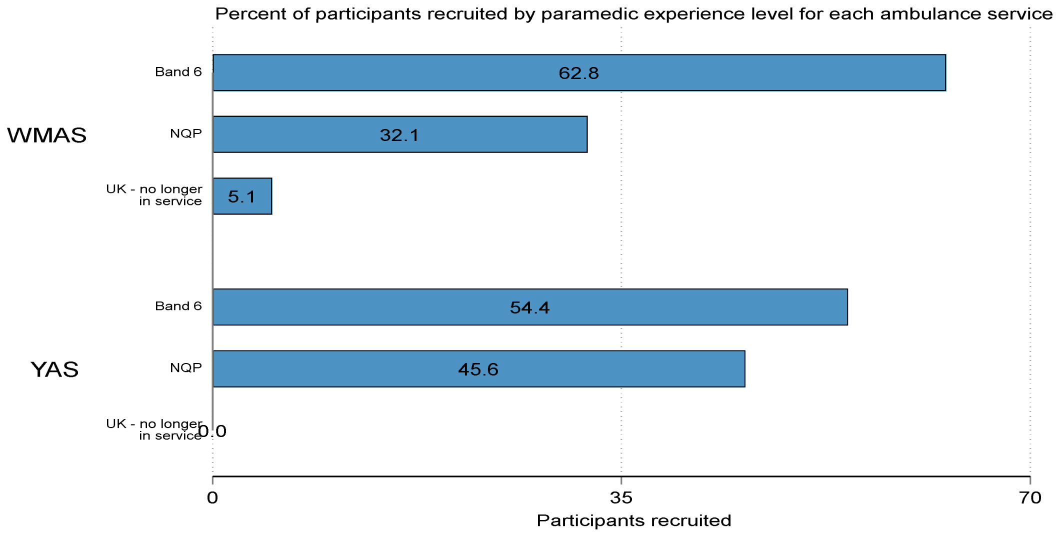

Figure 6: Percent of paramedics trial trained by experience level for each ambulance service.

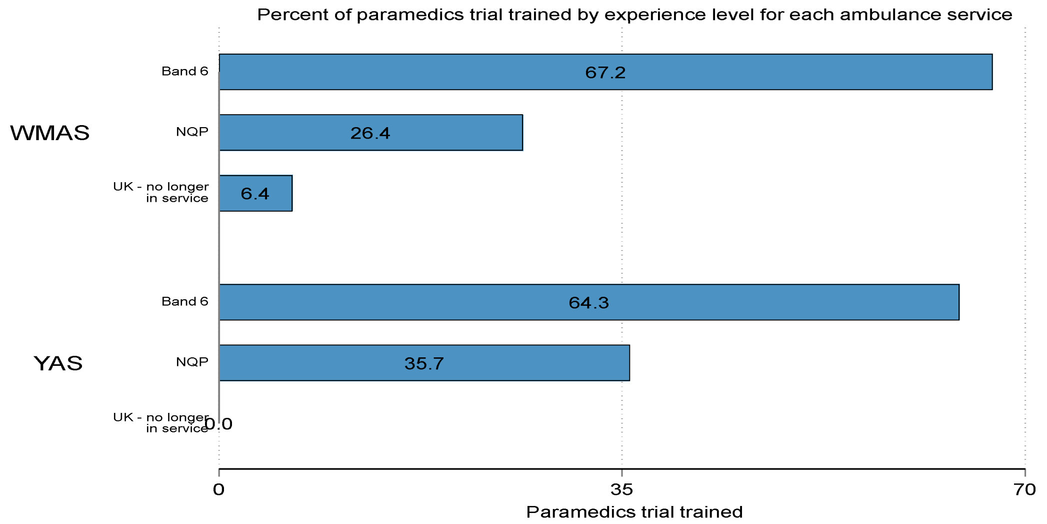

Figure 7a: Paramedic experience level percentages by ambulance service

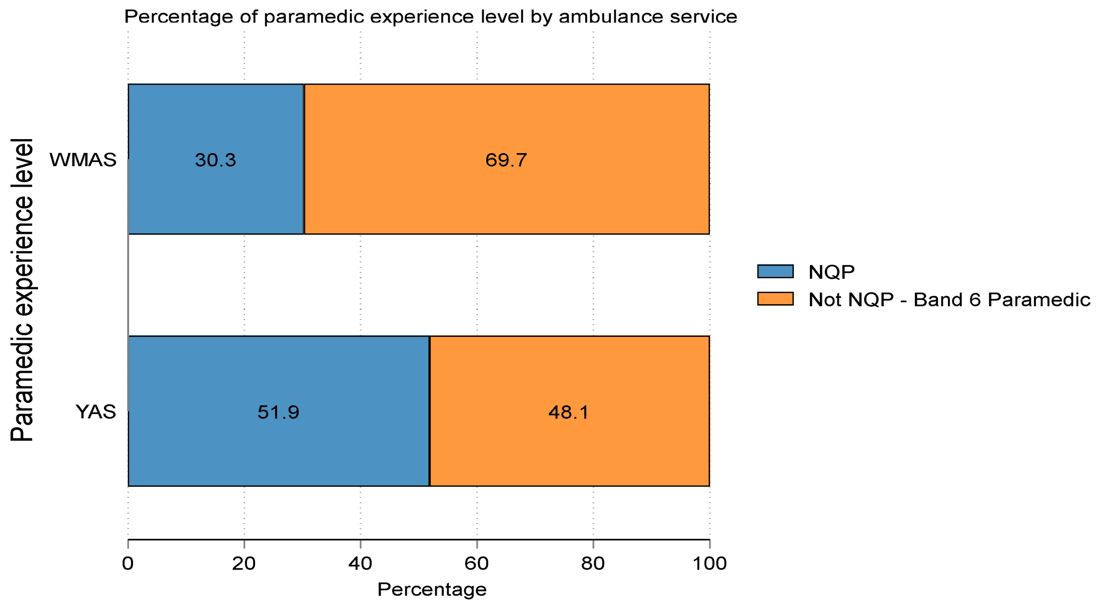

Figure 7b: Paramedic experience level percentages for all paramedics.

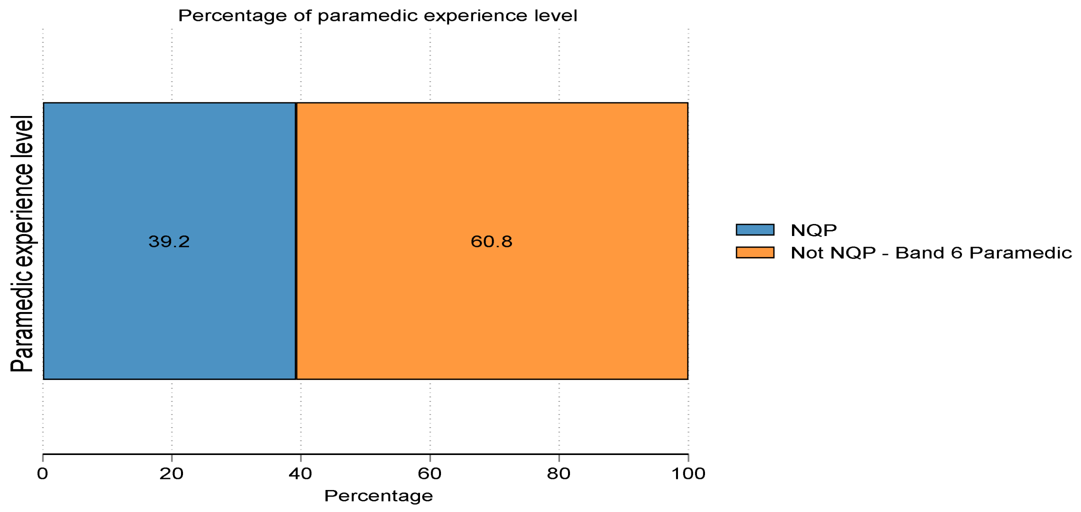

Figure 8: Adverse events by paramedic experience level

Figure 9: Non compliances by paramedic experience level

Figure 10: Proportion of participants experiencing adverse events by treatment arm and relative treatment arm difference as relative risk with 95% CI.

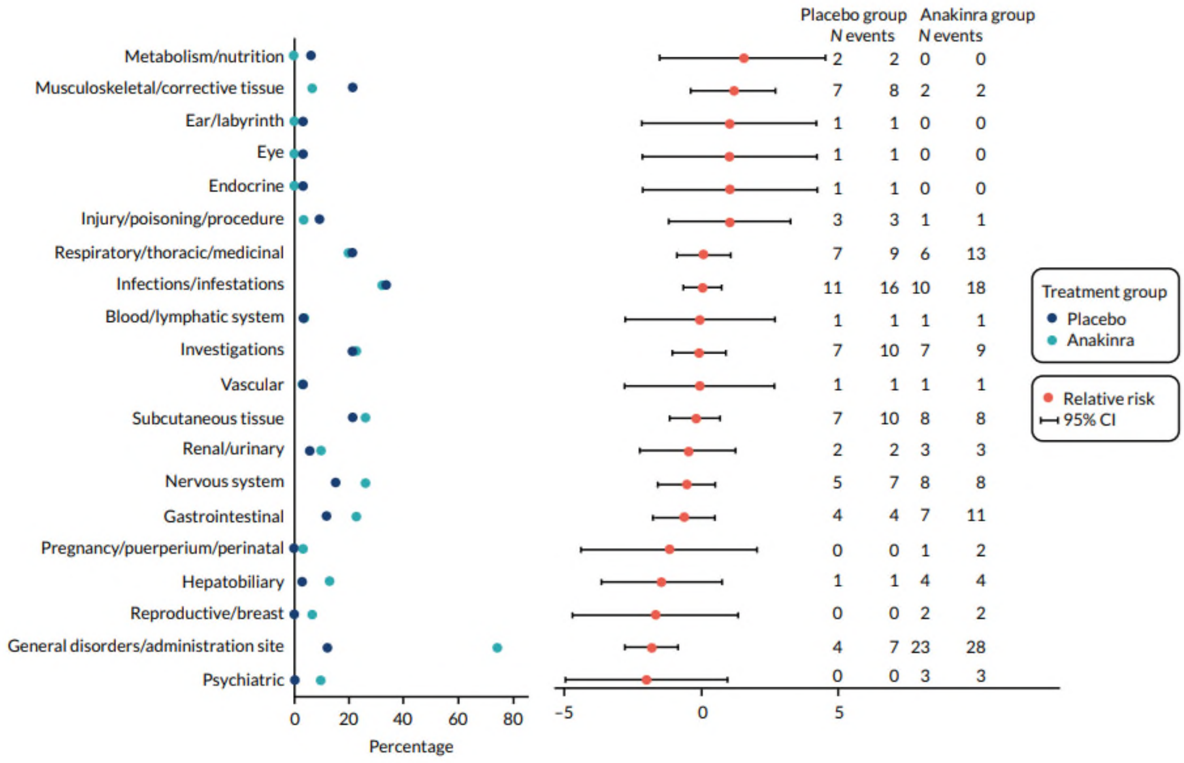

Figure 11: Graphical display of pain score over time summarised by treatment arm

Figure 12: Kaplan Meier plot for time to event outcomes

Figure 13: Dose response curve

Figure 14: Cost effectiveness Acceptability Curve (CEAC) showing the probability that the intervention is cost-effective across a range of cost-effectiveness thresholds.

Appendix 1: Mixed effect model outputs of vital signs and Glasgow coma scale

Variables used for adjusted model are defined as such
Agecat = 1 if age is <60, agecat =2 if age >=60
Asd_gender=1 if female, asd_gender=2 if male
Paracetamol = 1 if no paracetamol given as analgesia prior to randomisation, paracetamol = 2 if paracetamol given prior to randomisation

Appendix 1a: Unadjusted model output for respiration rate

```
Mixed-effects REML regression                   Number of obs     =      1,907
Group variable: tno                             Number of groups  =        394
                                                Obs per group:
                                                              min =          1
                                                              avg =        4.8
                                                              max =         13
                                                Wald chi2(1)      =       0.00
Log restricted-likelihood = -4114.6048          Prob > chi2       =     0.9522

------------------------------------------------------------------------------------
pvs_respiratoryrate | Coefficient  Std. err.      z    P>|z|     [95% conf. interval]
--------------------+---------------------------------------------------------------
           trialarm |
             Arm B  |  -.0190908   .3183001    -0.06   0.952    -.6429476    .6047659
              _cons |   18.60484    .222637    83.57   0.000     18.16848     19.0412
------------------------------------------------------------------------------------

-----------------------------------------------------------------------------
  Random-effects parameters  |   Estimate   Std. err.     [95% conf. interval]
-----------------------------+-----------------------------------------------
tno:                 (empty) |
-----------------------------+-----------------------------------------------
Residual: AR(1)              |
                         rho |   .8613154   .0090987       .842386    .8781218
                      var(e) |   12.82894   .7455218      11.44788     14.3766
-----------------------------------------------------------------------------
LR test vs. linear model: chi2(1) = 1905.00               Prob > chi2 = 0.0000
```

Appendix 1b: Adjusted model outputs for respiration rate

```
Mixed-effects REML regression                   Number of obs     =      1,907
Group variable: tno                             Number of groups  =        394
                                                Obs per group:
                                                              min =          1
                                                              avg =        4.8
                                                              max =         13
                                                Wald chi2(4)      =       2.34
```

```
Log restricted-likelihood = -4113.6781         Prob > chi2       =     0.6729

------------------------------------------------------------------------------------
pvs_respiratoryrate | Coefficient  Std. err.      z    P>|z|     [95% conf. interval]
--------------------+---------------------------------------------------------------
          trialarm |
            Arm B  |  -.0417404   .3192342    -0.13   0.896    -.6674279    .5839471
          2.agecat |  -.0830416   .3354906    -0.25   0.805     -.740591    .5745078
      2.asd_gender |   .4122983   .3288085     1.25   0.210    -.2321545    1.056751
     2.paracetamol |   .3125993   .4659268     0.67   0.502    -.6006005    1.225799
             _cons |   18.42936   .3631547    50.75   0.000     17.71759    19.14113
------------------------------------------------------------------------------------

------------------------------------------------------------------------------
  Random-effects parameters  |   Estimate   Std. err.     [95% conf. interval]
-----------------------------+------------------------------------------------
tno:                 (empty) |
-----------------------------+------------------------------------------------
Residual: AR(1)              |
                         rho |   .8614311   .0091246      .8424436    .8782816
                      var(e) |   12.84127   .7494194      11.45332     14.3974
------------------------------------------------------------------------------
LR test vs. linear model: chi2(1) = 1897.91              Prob > chi2 = 0.0000
```

Appendix 1c: Unadjusted model outputs for oxygen rate

```
Mixed-effects REML regression                   Number of obs     =      1,914
Group variable: tno                             Number of groups  =        395
                                                Obs per group:
                                                              min =          1
                                                              avg =        4.8
                                                              max =         13
                                                Wald chi2(1)      =       4.40
Log restricted-likelihood = -4319.5418          Prob > chi2       =     0.0359

--------------------------------------------------------------------------------
pvs_oxygenrate | Coefficient  Std. err.      z    P>|z|     [95% conf. interval]
---------------+----------------------------------------------------------------
      trialarm |
        Arm B  |   .4854634   .2314357     2.10   0.036     .0318577    .9390691
         _cons |   96.59618   .1624591   594.59   0.000     96.27777     96.9146
--------------------------------------------------------------------------------

------------------------------------------------------------------------------
  Random-effects parameters  |   Estimate   Std. err.     [95% conf. interval]
-----------------------------+------------------------------------------------
tno:                 (empty) |
```

```
------------------------------+------------------------------------------------
Residual: AR(1)               |
                          rho |   .6930673   .0150728      .6623478    .7214602
                       var(e) |   8.974873   .4081214       8.20958    9.811505
-------------------------------------------------------------------------------
LR test vs. linear model: chi2(1) = 1082.89               Prob > chi2 = 0.0000
```

Appendix 1d: Adjusted model outputs for oxygen rate

```
Mixed-effects REML regression                   Number of obs     =      1,914
Group variable: tno                             Number of groups  =        395
                                                Obs per group:
                                                              min =          1
                                                              avg =        4.8
                                                              max =         13
                                                Wald chi2(4)      =      95.24
Log restricted-likelihood = -4279.7556          Prob > chi2       =     0.0000

--------------------------------------------------------------------------------
pvs_oxygenrate | Coefficient  Std. err.      z    P>|z|     [95% conf. interval]
---------------+----------------------------------------------------------------
      trialarm |
        Arm B  |    .508935   .2129455     2.39   0.017     .0915696    .9263004
      2.agecat |  -1.832109   .2246709    -8.15   0.000    -2.272456   -1.391763
  2.asd_gender |   .5650903   .2192831     2.58   0.010     .1353033    .9948773
 2.paracetamol |   .2303191   .3219975     0.72   0.474    -.4007845    .8614226
         _cons |   97.38945     .24296   400.85   0.000     96.91325    97.86564
--------------------------------------------------------------------------------

-------------------------------------------------------------------------------
  Random-effects parameters   |   Estimate   Std. err.     [95% conf. interval]
------------------------------+------------------------------------------------
tno:                  (empty) |
------------------------------+------------------------------------------------
Residual: AR(1)               |
                          rho |   .6568152   .0162042      .6238814    .6874202
                       var(e) |   8.015194   .3529304      7.352472    8.737651
-------------------------------------------------------------------------------
LR test vs. linear model: chi2(1) = 931.70                Prob > chi2 = 0.0000
```

Appendix 1e: Unadjusted model output for heart rate

```
Mixed-effects REML regression                   Number of obs     =      1,961
Group variable: tno                             Number of groups  =        396
                                                Obs per group:
                                                              min =          1
                                                              avg =        5.0
```

```
                                                     max =         13
                                           Wald chi2(1)      =       0.30
Log restricted-likelihood = -7414.4142     Prob > chi2       =     0.5849

------------------------------------------------------------------------------
pvs_heartrate | Coefficient  Std. err.      z    P>|z|     [95% conf. interval]
--------------+----------------------------------------------------------------
     trialarm |
       Arm B  |  -.7248242   1.327059    -0.55   0.585    -3.325812    1.876164
        _cons |   81.58641   .9316761    87.57   0.000     79.76036    83.41246
------------------------------------------------------------------------------

------------------------------------------------------------------------------
  Random-effects parameters  |   Estimate   Std. err.     [95% conf. interval]
-----------------------------+------------------------------------------------
tno:                 (empty) |
-----------------------------+------------------------------------------------
Residual: AR(1)              |
                         rho |   .7966924   .0115647      .7728794    .8182646
                      var(e) |   252.2641   12.97784      228.0684    279.0268
------------------------------------------------------------------------------
LR test vs. linear model: chi2(1) = 1622.35           Prob > chi2 = 0.0000
```

Appendix 1f: Adjusted model output for heart rate

```
Mixed-effects REML regression              Number of obs     =      1,961
Group variable: tno                        Number of groups  =        396
                                           Obs per group:
                                                        min =          1
                                                        avg =        5.0
                                                        max =         13
                                           Wald chi2(4)      =       8.28
Log restricted-likelihood = -7406.3986     Prob > chi2       =     0.0818

------------------------------------------------------------------------------
pvs_heartrate | Coefficient  Std. err.      z    P>|z|     [95% conf. interval]
--------------+----------------------------------------------------------------
     trialarm |
       Arm B  |  -.6438985   1.322797    -0.49   0.626    -3.236533    1.948736
     2.agecat |  -3.018463   1.394566    -2.16   0.030    -5.751763   -.2851632
 2.asd_gender |   -3.02761   1.363022    -2.22   0.026    -5.699085   -.3561355
2.paracetamol |    .632973   1.955536     0.32   0.746    -3.199807    4.465753
        _cons |   84.71732   1.512028    56.03   0.000      81.7538    87.68084
------------------------------------------------------------------------------

------------------------------------------------------------------------------
  Random-effects parameters  |   Estimate   Std. err.     [95% conf. interval]
```

```
------------------------------+------------------------------------------------
tno:                 (empty) |
------------------------------+------------------------------------------------
Residual: AR(1)               |
                          rho |   .7951445   .0116504      .7711567    .8168779
                       var(e) |   250.2783   12.88711      226.2528    276.8551
-------------------------------------------------------------------------------
LR test vs. linear model: chi2(1) = 1610.34               Prob > chi2 = 0.0000
```

Appendix 1g: Unadjusted model output for blood pressure systolic

```
Mixed-effects REML regression                   Number of obs     =      1,963
Group variable: tno                             Number of groups  =        396
                                                Obs per group:
                                                              min =          1
                                                              avg =        5.0
                                                              max =         13
                                                Wald chi2(1)      =       5.45
Log restricted-likelihood = -8420.6019          Prob > chi2       =     0.0195

------------------------------------------------------------------------------------
-----
pvs_bloodpressuresystolic | Coefficient  Std. err.   z    P>|z|   [95% conf. interval]
--------------------------+---------------------------------------------------------
-----
                 trialarm |
                   Arm B  |   4.718615   2.020337   2.34  0.020   .7588267    8.678403
                    _cons |   142.3992   1.417528  100.46 0.000  139.6209    145.1775
------------------------------------------------------------------------------------
-----


------------------------------------------------------------------------------
  Random-effects parameters  |   Estimate   Std. err.     [95% conf. interval]
-----------------------------+------------------------------------------------
tno:                 (empty) |
-----------------------------+------------------------------------------------
Residual: AR(1)              |
                         rho |   .7599832   .0130589      .7331872    .7844228
                      var(e) |   622.0132   30.67074      564.7131    685.1275
------------------------------------------------------------------------------
LR test vs. linear model: chi2(1) = 1374.40              Prob > chi2 = 0.0000
```

Appendix 1h: Adjusted model output for blood pressure systolic

```
Mixed-effects REML regression                   Number of obs     =      1,963
Group variable: tno                             Number of groups  =        396
                                                Obs per group:
                                                              min =          1
                                                              avg =        5.0
                                                              max =         13
                                                Wald chi2(4)      =      65.46
Log restricted-likelihood =  -8387.478          Prob > chi2       =     0.0000

------------------------------------------------------------------------------------
pvs_bloodpressuresystolic | Coefficient  Std. err.   z   P>|z|  [95% conf. interval]
--------------------------+---------------------------------------------------------
                trialarm |
                  Arm B  |     4.6648   1.911931   2.44  0.015  .9174836   8.412116
                2.agecat |   14.76394   2.020733   7.31  0.000  10.80337    18.7245
            2.asd_gender |  -.4444625   1.971826   -0.23 0.822  -4.30917   3.420245
           2.paracetamol |  -1.838434   2.852469   -0.64 0.519  -7.429171  3.752304
                   _cons |   133.8585   2.190186   61.12 0.000  129.5658   138.1512
------------------------------------------------------------------------------------

------------------------------------------------------------------------------
  Random-effects parameters  |   Estimate   Std. err.     [95% conf. interval]
-----------------------------+------------------------------------------------
tno:                 (empty) |
-----------------------------+------------------------------------------------
Residual: AR(1)              |
                         rho |   .7401387   .0137664      .7119455    .7659498
                      var(e) |   573.3604   27.65366      521.6432    630.2051
------------------------------------------------------------------------------
LR test vs. linear model: chi2(1) = 1273.99             Prob > chi2 = 0.0000
```

Appendix 1i: Unadjusted model output for blood pressure diastolic

```
Mixed-effects REML regression                   Number of obs     =      1,955
Group variable: tno                             Number of groups  =        395
                                                Obs per group:
                                                              min =          1
                                                              avg =        4.9
                                                              max =         13
                                                Wald chi2(1)      =       2.20
Log restricted-likelihood = -7548.0427          Prob > chi2       =     0.1381

-------------------------------------------------------------------------------------
```

```
pvs_bloodpressurediastolic | Coefficient  Std. err.  z   P>|z|  [95% conf. interval]
---------------------------+--------------------------------------------------------
--------
                  trialarm |
                    Arm B  |    1.41135   .9518446  1.48   0.138  -.4542315 3.276931
                     _cons |   79.58655   .6680861  119.13 0.000  78.27713  80.89598
--------------------------------------------------------------------------------------
--------

------------------------------------------------------------------------------
  Random-effects parameters  |   Estimate   Std. err.     [95% conf. interval]
-----------------------------+------------------------------------------------
tno:                 (empty) |
-----------------------------+------------------------------------------------
Residual: AR(1)              |
                         rho |   .5793889   .0190089      .5409224    .6154366
                      var(e) |   183.5283   7.627245      169.1718    199.1031
------------------------------------------------------------------------------
LR test vs. linear model: chi2(1) = 624.73               Prob > chi2 = 0.0000
```

Appendix 1j: Adjusted model output for blood pressure diastolic

```
Mixed-effects REML regression                   Number of obs     =      1,955
Group variable: tno                             Number of groups  =        395
                                                Obs per group:
                                                              min =          1
                                                              avg =        4.9
                                                              max =         13
                                                Wald chi2(4)      =      11.75
Log restricted-likelihood = -7540.2694          Prob > chi2       =     0.0193

--------------------------------------------------------------------------------------
--------
pvs_bloodpressurediastolic | Coefficient  Std. err.  z   P>|z|  [95% conf. interval]
---------------------------+--------------------------------------------------------
--------
                  trialarm |
                    Arm B  |   1.466914   .9459209  1.55  0.121   -.387057   .320885
                  2.agecat |  -2.539433   1.003141  -2.53 0.011   -4.505553 -.5733123
              2.asd_gender |   1.035142   .9765654  1.06  0.289   -.8788909  2.949175
             2.paracetamol |  -.4914691   1.431659  -0.34 0.731   -3.297469  2.314531
                     _cons |   80.68343   1.088141  74.15 0.000   78.55071   82.81615
--------------------------------------------------------------------------------------
--------

------------------------------------------------------------------------------
  Random-effects parameters  |   Estimate   Std. err.     [95% conf. interval]
-----------------------------+------------------------------------------------
tno:                 (empty) |
```

```
------------------------------+------------------------------------------------
Residual: AR(1)               |
                          rho |   .5748942   .0192176      .5360119    .6113427
                       var(e) |   181.7909   7.554034      167.5722    197.2162
-------------------------------------------------------------------------------
LR test vs. linear model: chi2(1) = 607.48              Prob > chi2 = 0.0000
```

Appendix 1k: Unadjusted model output for Glasgow coma scale

```
Mixed-effects REML regression                   Number of obs     =      1,904
Group variable: tno                             Number of groups  =        392
                                                Obs per group:
                                                              min =          1
                                                              avg =        4.9
                                                              max =         13
                                                Wald chi2(1)      =       0.00
Log restricted-likelihood =  277.00011          Prob > chi2       =     0.9521

------------------------------------------------------------------------------
     pvs_gcs | Coefficient  Std. err.      z    P>|z|     [95% conf. interval]
-------------+----------------------------------------------------------------
    trialarm |
      Arm B  |  -.0007553   .0125708    -0.06   0.952    -.0253935    .0238829
       _cons |   14.97031   .0086623  1728.21   0.000     14.95334    14.98729
------------------------------------------------------------------------------

------------------------------------------------------------------------------
  Random-effects parameters  |   Estimate   Std. err.     [95% conf. interval]
-----------------------------+------------------------------------------------
tno:                 (empty) |
-----------------------------+------------------------------------------------
Residual: AR(1)              |
                         rho |   .3071933   .0215648      .2643438    .3488323
                      var(e) |   .0470252   .0016205      .0439539    .0503111
------------------------------------------------------------------------------
LR test vs. linear model: chi2(1) = 184.61              Prob > chi2 = 0.0000
```

Appendix 1l: Adjusted model output for Glasgow coma scale

```
Mixed-effects REML regression                   Number of obs     =      1,904
Group variable: tno                             Number of groups  =        392
                                                Obs per group:
                                                              min =          1
                                                              avg =        4.9
                                                              max =         13
                                                Wald chi2(4)      =       9.46
Log restricted-likelihood =  271.78157          Prob > chi2       =     0.0505
```

```
------------------------------------------------------------------------------
    pvs_gcs | Coefficient  Std. err.      z    P>|z|     [95% conf. interval]
-------------+----------------------------------------------------------------
    trialarm |
      Arm B  |  -.0001736   .0125264    -0.01   0.989    -.0247249    .0243777
    2.agecat |   -.032118   .0132914    -2.42   0.016    -.0581687   -.0060673
2.asd_gender |   .0127708   .0129694     0.98   0.325    -.0126487    .0381902
2.paracetamol |  -.0172589   .0187587    -0.92   0.358    -.0540252    .0195074
       _cons |   14.98584   .0143251  1046.13   0.000     14.95776    15.01392
------------------------------------------------------------------------------
```

PACKMaN
Paramedic Analgesia Comparing Ketamine and MorphiNe in trauma

ISRCTN:	ISRCTN14124474
Funding Body:	National Institute for Health Research
Ethics Approval:	West of Scotland REC 1, approved 01/09/2020
HEAP version number:	1.0
Date:	29/09/2021
Stage:	Final
Protocol version:	V3.0

Author signature: ________________________ date: ________________________

Senior statistician signature: ________________________ date: ________________________

Chief Investigator signature: ________________________ date: ________________________

WARWICK
CLINICAL TRIALS UNIT

Paramedic Analgesia Comparing Ketamine and MorphiNe in trauma (PACKMaN) trial - health economics Analysis Plan

Purpose of health economics analysis plan

The objective of the health economics analysis is to inform decision makers regarding the cost-effectiveness of paramedic administered ketamine compared to morphine for the management of acute severe pain from traumatic injury. The purpose of the health economics analysis plan (HEAP) is to outline the framework of methods that will be used to analyse the health economic components of the trial to ensure the integrity of the cost-effectiveness analysis.

1. Introduction

The PACKMaN trial is a multi-centre, double blinded randomised controlled trial (RCT) comparing the clinical and cost-effectiveness of ketamine compared to morphine for severe pain in acute traumatic injury. The trial is pragmatic and involves working with two large NHS ambulance trusts. Eligible patients (see trial protocol for eligibility criteria) reporting severe pain due to an acute injury will be randomised to either morphine or ketamine by trial paramedics. The trial will follow up participants up over a six-month period.

The two arms of the trial can be characterised as follows:

Intervention arm: The intervention arm will be administered ketamine hydrochloride. It will be supplied in glass ampoules containing 15mg in 1ml and supplied in numbered treatment packs containing 3 ampoules. The manufacture, clinical trials packaging, labelling and QP release will be arranged by MODEPHARMA. The ampoules will be labelled as trial related investigational medical product (IMP) and as such will be identical in appearance to the control. Further details about dosing schedule and methods of administration can be found within the trial protocol [1].

Control arm: The control arm will be administered morphine sulphate. Morphine sulphate will be supplied in glass ampoules containing 10mg in 1ml and supplied in numbered treatment packs containing 3 ampoules. The manufacture and clinical trials packaging, labelling and qualified person (QP) release will be arranged by MODEPHARMA. The ampoules will be labelled as trial related IMP and as such will be identical in appearance to the intervention. Further details about dosage schedule and methods of administration can be found in the protocol [1].

1.1 General principles for the primary health economic analysis

Given the trial is funded by the National Institute for Health Research, we will adopt principles that best meet the requirements of United Kingdom decision makers. The methods of economic evaluation will therefore be guided by the National Institute for Health and Care Excellence (NICE) guide to the methods of technology appraisal [2].

1.1.1 Type of economic evaluation

As recommended, the primary health economic analysis will be a cost-utility analysis with incremental quality adjusted life years (QALYs) as the primary health economic outcome [2]. Following NICE guidance, the EQ-5D-5L will be used for the construction of QALYs (see section 2.1.1) [2].

1.1.2 Perspective

A healthcare and personal social services (PSS) will be adopted in the primary analysis as recommended by NICE [2].

1.1.3 Time Horizon

The primary health economic analysis will run concurrently to the effectiveness analysis. The EQ-5D-5L will be collected at three and six-months post-randomisation. The time horizon will therefore be the 6-month period post-randomisation. Should outcomes not have converged after 6 months, we will consider the development of a decision analytic model to extrapolate the cost-effectiveness results over a lifetime horizon (see section 5.5).

1.1.4 Discounting

Given the trial-based analysis has a time-horizon of 6 months, costs and QALYs will not be discounted. Should longer-term decision modelling be conducted, we will use the 3.5% annual discount rate as recommended by NICE to discount future costs and QALYs [2].

1.1.5 Intention to treat

The health economic analysis will adopt the principle of 'intention to treat' [3]. This means that the health economic analysis will analyse individuals according to the trial arms to which they were randomised.

1.2 Missing data

Missing data is a common occurrence within randomised clinical trials and needs to be considered within the health economic analysis [4]. Missing data will be explored, and if non-trivial (5% or more in either costs or QALYs) [5], the base case analysis will use multiple imputation (MI) [6] as the preferred method for estimating results in the presence of missing data. MI uses the observed data and samples from the predictive distribution to create multiple datasets [7]. Under the assumption of missing at random, this provides unbiased estimates; this allows uncertainty surrounding estimates to be maintained whilst allowing full use of the available data (see section 5.2).

2 Outcomes

2.1 Primary health economic outcome

As recommended by NICE, incremental quality adjusted life years (QALYs) will be used as the primary measure of health consequence for the health economic analysis [2].

2.1.1 Quality adjusted life years (QALYs)

Estimating QALYs

QALYs combine both mortality and morbidity into a single measure that can be compared across contexts within the healthcare service. To calculate QALYs it is necessary to combine a preference-based health-related quality of life outcomes with survival benefits. In this study, we are using the EQ-5D-5L [8] at two time points (3 months, 6 months). The EQ-5D-5L is a preference-based measure of health-related quality of life and is recommended by NICE for use in economic evaluation [2]. The measure contains a descriptive system with five dimensions of health, each containing five levels. There exist value-sets [9], [10] that allow the calculation of *utility* scores for any given set of responses to the EQ-5D-5L descriptive system. A utility score is a score on a cardinal scale indexed at zero and one, where zero represents death, and one represents full health (negative states are possible). These utility values can be combined with survival benefits to derive QALYs. Although a new UK specific EQ-5D-5L value set exists [9], it has been a subject of controversy [11]. Currently NICE instead recommends [12] the use of the Van Hout et al [10] 'cross-walk' algorithm. This however may change in the interim period between the period of writing and the date of analysis. The choice of value set will therefore be made closer to the date of analysis and will be chosen in accordance with NICE guidelines at the point of analysis.

A challenge to the analysis is the lack of baseline EQ-5D-5L measurement which is required for the calculation of QALYs for the duration of follow-up from randomisation. For ethical, logistical and pragmatic reasons, it is not possible to capture baseline EQ-5D-5L measurements in patients suffering acute pain following trauma within this trial. This is not uncommon within trials involving emergency and critical care settings [13]. Ideally, the EQ-5D-5L would be completed at the time of randomisation or as soon as possible afterwards. This however is not possible in this trial. A systematic review of emergency and critical care studies [13] identified four strategies that have been used in such situations. First, the most common approach (57% of all studies identified by the systematic review) is to assign a fixed health utility to all participants at baseline. Second, some studies (29%) estimated QALYs using only the available data and implicitly ignored any benefits that occurred before the first follow up data collection point. One study (7%) asked patients to retrospectively recall their health state at randomisation. Finally, one study (7%) mapped health states onto EQ-5D-3L using expert evidence to derive baseline health states. The primary analysis will use a fixed baseline approach for all participants. This fixed value will be derived by mapping the 'typical' acute pain trauma case to the EQ-5D-5L using expert opinion. The sensitivity of this assumption will be tested within sensitivity analyses (see 5.4.2). Sensitivity analyses will include assigning different values to patients according to severity as determined by registration to the Trauma Audit and Research Network (TARN). TARN can be used as proxy for severity as the most serious trauma patients will be registered onto TARN whilst less severe cases will not (non-TARN). We will then use expert opinion to estimate a baseline EQ-5D profile for both TARN and non-TARN patients.

QALYs for each patient will be calculated by using the utility values at baseline, 3 months and 6 months. QALYs will be calculated by linearly interpolating utility values at the three time points and calculating the area under the curve using the trapezium rule [14]. QALYs will be calculated for each patient in the trial.

3 Resource use and costing

To calculate costs for use in cost-utility analysis it is necessary to capture information on resources used for both the control and intervention arm. Costs within this trial can be split into the following broad components:

- Direct intervention costs (medication costs)
- Direct healthcare and PSS costs (e.g. medication for side-effects, outpatient appointments, community care)
- Other societal costs (e.g. value of lost productivity, out of pocket expenses)

NICE's guide to methods of technology appraisal recommend costing from an NHS and personal social services (PSS) perspective [2]. The primary analysis will only consider the first two categories of costs; broader societal costs will be included within a sensitivity analysis. To calculate costs, it is first necessary to capture resource use, and then apply unit costs to each resource input. The price year for the analysis will be informed by the latest available base year for common costing resources at time of analysis.

3.1 Direct intervention resource use and costs

The PACKMaN trial focuses on administration of two alternative medications for pain relief in patients with severe pain. The intervention arm will receive ketamine hydrochloride whilst the control arm will receive morphine sulphate. The control arm however receives just one 30-minute practitioner appointment in conjunction with direction to freely available on-line resources. The intervention components and associated resource use are summarised within Table 1 below. This table shows what the components are, how they will be collected and where unit cost sources may be sourced from.

Table 1: Direct intervention resource use and cost sources

Intervention arm			
**Resource type**	**Resource use**	**How collected**	**Unit costs source**
Ketamine hydrochloride	Number and ml of doses administered.	Recorded within ambulance service data	NHSBSA [15]
Control arm			
Morphine sulphate	Number and ml of doses administered.	Recorded within ambulance service data	NHSBSA [15]

3.2 Healthcare and social care resource use

In according with NICE guidance, we will capture healthcare and PSS costs for both arms of the trial [2]. This will include, within-ambulance costs, inpatient care, outpatient care, community care, accident and emergency admission, medication, and personal social services. The methods for capturing the resource use and the sources for unit costs are outlined in Table 2 below. Within ambulance costs will be captured through the ambulance service data form, index admission costs will be collected via the hospital data collection form, whilst the remaining costs will be collected through the case report forms at 3 and 6 months.

Table 2: Health and social care costs for both arms

**Resource type**	**Resource use**	**How collected**	**Unit cost sources**

Within ambulance – rescue analgesia medication	Entonox, paracetamol, ibuprofen, other painkiller	Ambulance service data form	NHSBSA [15]
Within ambulance – side-effects medication	Midazolam or naloxone	Ambulance service data form	NHSBSA [15]
Inpatient care – index admission	Length of stay and number of days at each level of care	Hospital data collection form	NHS Reference Costs [16]
Index admission	CT scans	Hospital data collection form	NHS Reference Costs [16]
Inpatient care – follow up	Specified within CRFs	CRFs at 3m and 6m	NHS Reference Costs [16] and PSSRU [17]. HRG4+ 'Code to Group' [18] used to allocate inpatient care to HRG groups for costing.
Outpatient care	Specified within CRFs	CRFs at 3m and 6m	NHS Reference Costs [16] and PSSRU [17].
Community care	Specified within CRFs	CRFs at 3m and 6m	NHS Reference Costs [16] and PSSRU [17].
Medication	Specified within CRFs	CRFs at 3m and 6m	NHSBSA [15]
Personal social services	Specified within CRFs	CRFs at 3m and 6m	PSSRU [17] unit costs

3.3 Wider costs

Within an addition sensitivity analysis, we will also be collecting information related to days lost from work and out of pocket expenses.

Table 3: Wider costs

Resource type	Resource use	How collected	Unit cost sources
Absence from work and out of pocket expenses	Specified within CRFs	CRFs at 3m and 6m	Stated in CRFs

4 Data integrity

On arrival in the trial office, data will be examined by trial staff to ensure its integrity. Blinded descriptive data will be routinely reported and presented to the data monitoring committee (DMC), this includes proportion of missingness of the health economic variables. Any questionable data (e.g. outliers/high missingness) will be queried and followed up if necessary. The DMCs will provide an opportunity to reflect whether the processes being used to collect data need refining or whether current methods are performing as desired. All data will be stored on secure University of Warwick servers in encrypted folders and access will be limited to only those approved to use it. Subsequently at the health economic analysis stage, variables will be range-checked and implausible values queried.

5 Statistical analysis

5.1 Descriptive analysis

Resource use, costs and EQ-5D utility scores will first be presented descriptively to inform parameters for future health economic studies (this includes means and standard deviations). Costs will be calculated for all perspectives outlined previously.

5.2 Addressing missing data with multiple imputation

If the proportion of missing data for either costs or QALYs is more than 5% we will use multiple imputation to impute data within the base-case analysis. This data will then be used in the incremental analysis of costs, QALYs and the joint cost-effectiveness analysis. A complete case analysis will be included as a sensitivity analysis. Stata [20] will be used to conduct both the multiple imputation and the analysis of imputed data. The 'mi impute chained' command which uses chained equations to generate imputed datasets will be used for each treatment group. Within the imputation regression framework, we will include both costs and EQ-5D-5L at each timepoint as both imputed and predictor variables. We will also include any auxiliary variables that are found to be highly correlated (r>0.4) [21] with costs or EQ-5D-5L, or are believed to be associated with missingness. We will use predictive mean matching drawing from the 5 nearest 'neighbours', this is important for the avoidance of drawing implausible values, e.g., utility values over 1, and 'negative costs'. The number of iterations will be guided by the fraction of missing information [5]. We will then use the 'mi estimate' functionality within Stata to run the analyses (specified in subsequent sections) within each dataset and to combine results using Rubin's combination rule to allow inferential statistics. Imputations will be added until estimates stabilise. We will examine the validity of the imputed data by comparing the distribution of the imputations and observed data both visually and statistically.

5.3 Single end point analysis: incremental costs and incremental QALYs

Before conducting the joint cost-effectiveness analysis, we will examine the impact of the intervention on incremental costs and incremental QALYs in isolation. Differences between the two arms will be assessed using a regression framework. The exact specification will depend upon the nature of the data. Costs will be estimated by combining resource use data with unit costs. Costs for each patient within the trial will be calculated and incremental costs between the two arms will be estimated. Again, a regression framework will be used, and its exact specification will be informed by the nature and distribution of the data.

5.4 Cost-effectiveness analysis and characterising uncertainty

We will use bivariate regression analysis in the form of seemingly unrelated regressions (with bootstrapping) for the joint analysis of costs and QALYs. This framework offers several benefits: first of all it accounts for the existence of correlation between costs and outcomes for patients; second it allows the inclusion of covariates within the analysis, this is particularly relevant for the adjustment of baseline utility with respect to QALYs accrued; third it is generally robust to non-normal distributions; fourth, it can account for clustering either by including clusters as a fixed effect or by running the seemingly unrelated regressions in a multi-level framework. Non-parametric bootstrapping will be used to examine the level of uncertainty by presenting the bootstrapped results on a cost-effectiveness plane, and by generating cost-effectiveness acceptability curves (CEACs). Should there be distributional or computational concerns (e.g. difficulty in fitting a multi-level seemingly unrelated regression model with imputed data in Stata) then we may consider combining costs and outcomes within a univariate net-benefit regression framework.

5.4.1 Characterizing uncertainty for decision makers

CEACs will be used to characterise uncertainty. CEACs show the probability that the intervention is cost-effective compared to the control at different levels of willingness to pay for QALYs and explicitly highlight the uncertainty within the decision problem. To avoid the issues related to uncertainty around cost-effectiveness ratios we will calculate net-monetary benefit for each of the bootstrapped iterations:

$$\Delta NB = \Delta e\gamma - \Delta c$$

In this instance, ΔNB refers to the incremental net monetary benefit, Δe reflects the incremental outcome of interest, incremental QALYs, whilst Δc refers to the incremental costs. The symbol γ refers to the decision maker's willingness to pay per QALY. For each of the bootstrapped cost-effectiveness samples we will calculate the associated net-monetary benefit across a range of levels of willingness to pay (γ). For each γ the proportion of iterations where net-benefit is greater than zero can be used estimate the probability that the intervention is more cost-effective at that willingness to pay. This will be conducted for a range of γ including £20,000 and £30,000 per QALY as specified by NICE and plotted to derive a CEAC [2].

5.4.2. Sensitivity analyses

In addition to the probabilistic sensitivity analysis outlined above we will also consider sensitivity analyses, these will include:

- Costing from a societal perspective
- Complete case analysis (assuming missing data exceeds 5%)
- Changing baseline utility assumptions
- Subgroup analyses as specified within the statistical analysis plan
- Cost per sum of pain intensity difference (SPID) score point reduction

5.5 Decision modelling

The primary trial-based analysis will focus on the costs and QALYs accrued during the trial period. There however is potential for costs and benefits to accrue beyond the trial period. If outcomes have not converged by the 6m timepoint we will consider extrapolating the results over a longer time horizon using a decision analytic model. This would involve combining the trial data with external sources to estimate the long-term cost-effectiveness of the intervention. Any costs and benefits accruing after the first year would be discounted at a rate of 3.5% per year and full probabilistic sensitivity analysis would be conducted in line with the NICE reference case [2]. A decision as to the necessity of building a decision analytic model and its specification will be made following discussion between the health economists and the trial team following preliminary analysis of the data. This will be informed by considerations such as the conclusiveness and direction of within trial results. For example, if the control dominates the intervention and extrapolation would only increase the strength of this result then there is little need to extrapolate further as the intervention should be rejected.

5.6 Value of information analysis

Should a decision model be developed we will also conduct a value of information (VoI) analysis to examine the expected value of future research. The VoI analysis will entail the calculation of the expected value of perfect information (EVPI) using data from the cost-effectiveness analysis. EVPI can be conceptualised as the expected gain from eliminating uncertainty within the decision problem, or put another way, the expected loss associated with uncertainty. This is essentially the probability of the decision being wrong multiplied by the average consequence of being wrong [22].

This allows us to calculate the estimated value of 'perfect knowledge' which is the maximum value society should be willing to pay for additional evidence to reduce uncertainty around whether the intervention or the control is more cost-effective [23]. Using the trial data, we will calculate the per person EVPI using a willingness to pay threshold of £30,000 per QALY, this representing the threshold NICE uses in practice [24]. This will be multiplied by the number of potential beneficiaries of the intervention within the NHS along with the technological horizon (years) to estimate population EVPI. Discounting of EVPI will be applied at 3.5% beyond the first year.

6 Dummy Tables

Table 1: Completeness of data by follow-up visit

	Control		*Intervention*		*Total*	
	**n**	**(%, N)**	**n**	**(%, N)**	**n**	**(%, N)**
***Health status*[1]**						
EQ-5D Baseline (derived)						
EQ-5D 3 months						
EQ-5D 6 months						
EQ-5D All visits						
***Resource use*[2]**						
Inpatient						
Outpatient						
Community						
Personal social services						
Wider costs						

1.EQ-5D-5L index score
2. Range shown (3M,6M)

Table 2: Health Status, resource use and cost (complete cases)

	Control		*Intervention*		*Total*	
	**mean**	**(SD)**	**mean**	**(SD)**	**mean**	**(SD)**
Health status[1]						
EQ-5D Baseline						
EQ-5D 3 months						
EQ-5D 6 months						
QALYs						
Resource use *(all visits)*						
Inpatient nights						
Outpatient visits						
A&E Visits						
Community						
GP surgery visits						
GP home visits						
GP telephone contacts						
GP video/online contacts						
District nurse contacts						
Social worker contacts						
Physiotherapy contacts						
Occupational therapy contacts						
Counsellor						
Psychologist						
Home help/carer						
Other community contacts						
Medication						
Special Equipment						
Wider costs						
Cost[2]						
A: Cost (study procedures)						
B: Cost (NHS contacts)						
C: Cost (Personal social services)						
Cost (Total, A+B+C)						

1 EQ-5D-5L index score

2 Wider costs are not included in the analytic perspective, which includes only health service and personal social services costs

Table 3: Cost-effectiveness results

		Incremental cost (95%CI)	Incremental QALYs (95%CI)	ICER (95%CI)	p[1]	p[2]	NMB[1]	NMB[2]
Base case								
	Imputed costs and QALYs, baseline EQ-5D adjusted							
Sensitivity analyses								
1	Inclusion of societal costs							
2	Complete case analysis							
3	Base case: sub-group analyses specified in the SAP							
4	Baseline utility assumptions changes							
5	Cost per SPID point reduction							

[1] probability cost-effective or net monetary benefit if willing to pay £20,000/QALY. [2] probability cost-effective or net monetary benefit if willing to pay £30,000/QALY

7 References

[1] 'Paramedic Analgesia Comparing Ketamine and MorphiNe in trauma : PACKMaN'. 2021. [Online]. Available: https://warwick.ac.uk/fac/sci/med/research/ctu/trials/packman/health/packman_protocol_v3.0_09.03.2021_clean.pdf

[2] NICE, *Guide to the methods of technology appraisal 2013*. NICE, 2013. Accessed: Jul. 28, 2014. [Online]. Available: https://www.nice.org.uk/article/PMG9/chapter/5-The-reference-case

[3] S. Ramsey *et al.*, 'Cost-Effectiveness Analysis Alongside Clinical Trials II - An ISPOR Good Research Practives Task Force Report', *Value Health*, vol. 18, pp. 161–172, 2015, doi: 10.1016/j.jval.2015.02.001.

[4] M. Gomes, K. Díaz-Ordaz, R. Grieve, and M. G. Kenward, 'Multiple imputation methods for handling missing data in cost-effectiveness analyses that use data from hierarchical studies: an application to cluster randomized trials.', *Med. Decis. Mak. Int. J. Soc. Med. Decis. Mak.*, vol. 33, no. 8, pp. 1051–63, Nov. 2013, doi: 10.1177/0272989X13492203.

[5] P. Madley-Dowd, R. Hughes, K. Tilling, and J. Heron, 'The proportion of missing data should not be used to guide decisions on multiple imputation', *J. Clin. Epidemiol.*, vol. 110, pp. 63–73, Jun. 2019, doi: 10.1016/j.jclinepi.2019.02.016.

[6] J. L. Schafer, 'Multiple imputation: a primer':, *Stat. Methods Med. Res.*, Jul. 2016, doi: 10.1177/096228029900800102.

[7] J. A. C. Sterne *et al.*, 'Multiple imputation for missing data in epidemiological and clinical research: potential and pitfalls.', *BMJ*, vol. 338, no. jun29_1, p. b2393, Jan. 2009, doi: 10.1136/bmj.b2393.

[8] M. Herdman *et al.*, 'Development and preliminary testing of the new five-level version of EQ-5D (EQ-5D-5L)', *Qual. Life Res.*, vol. 20, no. 10, pp. 1727–1736, Dec. 2011, doi: 10.1007/s11136-011-9903-x.

[9] N. Devlin, K. Shah, Y. Feng, B. Mulhern, and B. van Hout, 'Valuing health-related quality of life : An EQ-5D-5L value set for England', *OHE Res. Pap.*, vol. January, no. January, 2016, [Online]. Available: https://www.ohe.org/publications/valuing-health-related-quality-life-eq-5d-5l-value-set-england

[10] B. van Hout *et al.*, 'Interim scoring for the EQ-5D-5L: mapping the EQ-5D-5L to EQ-5D-3L value sets.', *Value Health*, vol. 15, no. 5, pp. 708–715, 2012.

[11] C. Sampson, 'Bad reasons not to use the EQ-5D-5L', *The Academic Health Economists' Blog*, Mar. 23, 2018. https://aheblog.com/2018/03/23/bad-reasons-not-to-use-the-eq-5d-5l/ (accessed Apr. 29, 2020).

[12] NICE, 'Position statement on use of the EQ-5D-5L value set for England (updated October 2019) | NICE technology appraisal guidance | NICE guidance | Our programmes | What we do | About', *NICE*, 2019. https://www.nice.org.uk/about/what-we-do/our-programmes/nice-guidance/technology-appraisal-guidance/eq-5d-5l (accessed Apr. 29, 2020).

[13] M. Dritsaki, F. Achana, J. Mason, and S. Petrou, 'Methodological Issues Surrounding the Use of Baseline Health-Related Quality of Life Data to Inform Trial-Based Economic Evaluations of Interventions Within Emergency and Critical Care Settings: A Systematic Literature Review', *PharmacoEconomics*, vol. 35, no. 5, pp. 501–515, May 2017, doi: 10.1007/s40273-016-0485-x.

[14] A. Manca, N. Hawkins, and M. J. Sculpher, 'Estimating mean QALYs in trial-based cost-effectiveness analysis: the importance of controlling for baseline utility.', *Health Econ.*, vol. 14, no. 5, pp. 487–96, May 2005, doi: 10.1002/hec.944.

[15] 'Prescription Cost Analysis (PCA) data | NHSBSA'. https://www.nhsbsa.nhs.uk/prescription-data/dispensing-data/prescription-cost-analysis-pca-data (accessed May 24, 2021).

[16] Department of Health, 'NHS reference costs 2014 to 2015', London, 2015.

[17] 'Unit Costs of Health and Social Care 2017 | PSSRU'. https://www.pssru.ac.uk/project-pages/unit-costs/unit-costs-2017/ (accessed Oct. 14, 2019).
[18] 'HRG4+ 2020/21 Local Payment Grouper', *NHS Digital*. https://digital.nhs.uk/services/national-casemix-office/downloads-groupers-and-tools/local-payment-2020-21 (accessed May 14, 2020).
[19] 'Prescription Cost Analysis', *NHS Digital*. https://digital.nhs.uk/data-and-information/publications/statistical/prescription-cost-analysis (accessed May 14, 2020).
[20] StataCorp, *Stata statistical software: Release 16*. College Station, TX: StataCorp LLC, 2019.
[21] UCLA IDRE, 'Multiple Imputation in Stata'. https://stats.idre.ucla.edu/stata/seminars/mi_in_stata_pt1_new/ (accessed May 14, 2020).
[22] E. C. F. Wilson, 'A Practical Guide to Value of Information Analysis', *PharmacoEconomics*, vol. 33, no. 2, pp. 105–121, Feb. 2015, doi: 10.1007/s40273-014-0219-x.
[23] H. V. Eeren, S. J. Schawo, R. H. J. Scholte, J. J. V. Busschbach, and L. Hakkaart, 'Value of Information Analysis Applied to the Economic Evaluation of Interventions Aimed at Reducing Juvenile Delinquency: An Illustration', *PLOS ONE*, vol. 10, no. 7, p. e0131255, Jul. 2015, doi: 10.1371/journal.pone.0131255.
[24] K. Claxton *et al.*, 'Methods for the estimation of the National Institute for Health and Care Excellence cost-effectiveness threshold', *Health Technol. Assess. Winch. Engl.*, vol. 19, no. 14, pp. 1–503, v–vi, Feb. 2015, doi: 10.3310/hta19140.
